# Synthesis of 1,2,3-triazole-piperazin-benzo[*b*][1,4]thiazine 1,1-dioxides: antibacterial, hemolytic and *in silico* TLR4 protein inhibitory activities†

**DOI:** 10.1039/d3ra07509e

**Published:** 2024-03-18

**Authors:** Nagavelli Ramu, Thupurani Murali Krishna, Ravikumar Kapavarapu, Sirassu Narsimha

**Affiliations:** a Department of Chemistry, Chaitanya Deemed to be University Hyderabad Telangana India narsimha.s88@gmail.com narsimha.s88@chaitanya.edu.in; b Department of Biotechnology, Chaitanya Deemed to be University Hyderabad Telangana India; c Department of Pharmaceutical Chemistry and Phytochemistry, Nirmala College of Pharmacy Atmakur Mangalgiri Andhra Pradesh India

## Abstract

In this study, we designed and synthesized a number of novel 1,2,3-triazole-piperazin-benzo[*b*][1,4]thiazine 1,1-dioxide derivatives and investigated their *in vitro* antibacterial and hemolytic activity. When compared to the lead chemical, dicloxacillin, the majority of the compounds demonstrated acceptable activity. Among them, the most promising compounds 6e, 6g, 6i, 8d, and 8e exhibited excellent antibacterial activity against the methicillin-susceptible *S. aureus* (MSSA), methicillin-resistant *S. aureus* (MRSA), and vancomycin-resistant *S. aureus* (VRSA) with MIC values of 1.56 ± 0.22 to 12.5 ± 1.75 μg mL^−1^, respectively, The percentage of hemolysis ranged from 21.3 μg mL^−1^ to 33.8 μg mL^−1^. Out of the six compounds (6i, 6e, 6f, 6g, 8e, 8d) tested compound 8e and 8d displayed minimal or negligible hemolytic activity across all the tested concentrations 29.6% and 30.2% recorded at 100 μg mL^−1^ concentration respectively. *In silico* docking studies were performed to evaluate the molecular interactions of 6e, 6f, 6g, 6i, 8d, and 8e compounds with Human, Mouse and Bovine TLR4 proteins (PDB: 3FXI, 3VQ1, 3RG1) and observed that three of the compounds (6i, 8d, and 8i) had appreciable binding energies ranging from −8.5 to −9.0 Kcal mol^−1^. Finally, the *in silico* pharmacokinetic profile was predicted for potent compounds 8d, 8e and 6i using SWISS/ADME, All compounds investigated in this study adhered to Lipinski's rule of five with slight deviation in molecular weight (8d and 8e).

## Abstract 2

Novel 1,2,3-triazoles (6a–6j & 8a–8g) were synthesized and evaluated for their antibacterial activity against *S. aureus*. The more potent compounds were further evaluated for their *in silico* TLR4 inhibitory activity.

## Graphical abstract

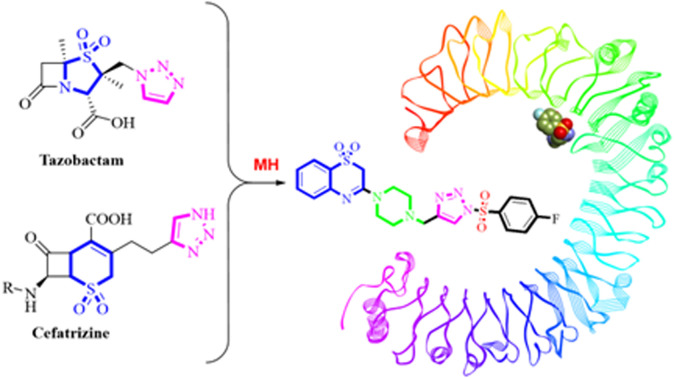

## Introduction

Overuse of currently available antibiotics, efflux mechanisms of bacteria, genetic modifications or mutations leads to the development of Multi-Drug Resistance (MDR) bacterial strains. One of the most important bacteria is *Staphylococcus aureus*, which has evolved and developed resistance to various antibiotics. Bacterial infections, a serious public health concern,^1^ have recently caused a huge increase in fatalities globally. On the other hand, the introduction of new antibiotics has decreased dramatically during the last decade. Thus, the discovery or creation of antimicrobial medicines with diverse chemical structures is critical for the treatment of bacterial infections and the consequent reduction of clinical drug resistance. Indeed, the World Health Organization (WHO) has warned that all known antibiotics may become ineffective against common pathogens in the near future, potentially leading to untreatable infections. As a result, there is an urgent need for global scientists to continue their efforts in the discovery and development of novel antibacterial agents with effective therapeutic activity.^2^

The importance of testing a medicine for its property of hemolysis cannot be understated, as it relates directly to the safety and efficacy of the medication.^3,4^ Hemolysis refers to the rupture or destruction of red blood cells, and this can have serious consequences for a patient's health. There are several reasons why testing for hemolysis is crucial in the development and use of medicines.^5^ The foremost concern in medicine development and administration is patient safety. Hemolysis can lead to severe health complications, including anemia, organ damage, and even death. Testing for hemolysis ensures that a medication does not pose a risk to a patient's blood cells.^6^ Hemolysis can also affect the effectiveness of a medicine. If a medication causes the destruction of red blood cells, it may hinder the body's ability to transport oxygen and nutrients to vital organs and tissues. Therefore, testing for hemolysis helps ensure that the drug remains effective in treating the targeted condition.^7^

Understanding a drug's potential for hemolysis is essential for determining the appropriate dosage. Medications that have a higher risk of causing hemolysis may need to be administered at lower doses or with specific precautions to minimize the risk to patients.^8^ Hemolysis-induced adverse reactions can be life-threatening. By identifying drugs that have the potential to cause hemolysis during the testing phase, healthcare providers can make informed decisions about whether to prescribe them and monitor patients more closely when necessary.^9^ Regulatory agencies, such as the U.S. Food and Drug Administration (FDA) and the European Medicines Agency (EMA), require thorough testing of new medications, including assessments of their potential to cause hemolysis. Compliance with these regulations is crucial for gaining approval to market a drug.^10^ Toll-like receptor 4 (TLR4) is a member of the pattern recognition receptor family, which is important for the human defence system and has a very sensitive and selective response to invasive pathogens.^11^ Numerous medications that target TLR4 are currently undergoing clinical trials, and the two FDA-approved medications, monophosphoryl lipid A (MPLA) for the treatment of bladder and cervical cancer and Bacillus Calmette–Guerin, a vaccine against tuberculosis, are readily available.^12^

**Fig. 1:**
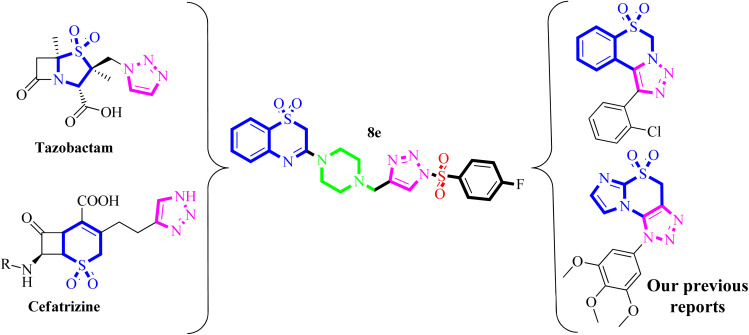
Design strategy for synthesis of 1,2,3-triazole-piperazin-benzo[*b*][1,4]thiazine 1,1-dioxides *via* molecular hybridization approach.

Because of their synthesis and fascinating pharmacological properties, 1,2,3-triazoles and their hybrids have emerged as “lead molecules” in drug discovery in recent years.^13–16^ As antibacterial agents, various 1,2,3-triazole-based compounds have been synthesized.^17^ In contrast, Fig. 1 shows a few commercially available medicines with 1,2,3-triazole containing Tazobactam and Cefatrizine are potent antibiotics.^18,19^ Similarly, sulfones are valuable synthetic intermediates for the construction of chemically and biologically important molecules.^20–22^ Aromatic sulfones have wide application in therapeutic study particularly as antipsychotic agents.^23^ Very recently, we have developed an efficient method for the synthesis of a novel class of 1,2,3-triazole containing sulfonyl derivatives.^24–31^ Based on the previous reports, we envisaged that the synthesized 1,2,3-triazole as well as their sulfone derivatives might show potent antibacterial (Fig. 1) hemolytic and *in silico* TLR4 protein inhibitory activities.

In view of the (i) significant roles of 1,2,3-triazoles, sulfonyl, and benzo[*b*][1,4]thiazine in the development of several antibacterial compounds, (ii) application of molecular hybridization approaches to access potent biologically active compounds, and (iv) in continuation of research work on the synthesis and biological evaluation of 1,2,3-traizole-based compounds. Herein, we planned to synthesize some new 1,2,3-triazole-piperazin-benzo[*b*][1,4]thiazine 1,1-dioxides and evaluated for their antibacterial, hemolytic and *in silico* TLR-4 protein inhibitory activities.

## Results and discussion

**Scheme 1:**
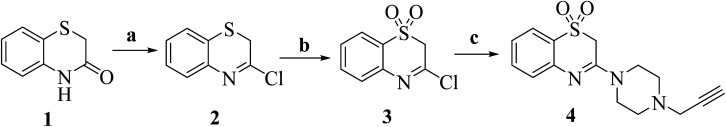
Reagents and conditions: (a) PCl_5_, reflux 180 °C, 1 h; (b) *m*-CPBA, DCM, 0–5 °C, 2 h; (c) 1-(prop-2-yn-1-yl)piperazine, TEA, dioxane, 80 °C, 8 h.

The synthesis of the title compounds is as outlined in Scheme 1. 2*H*-benzo[*b*][1,4]thiazin-3(4*H*)-one (1) in reaction with PCl_5_ in reflux condition yields the corresponding 3-chloro-2*H*-benzo[*b*][1,4]thiazine (2). Oxidation of compound 2 with 3-chlorobenzoperoxoic acid (*m*-CPBA) in dichloromethane at room temperature resulted in the formation of 3-chloro-2*H*-benzo[*b*][1,4]thiazine 1,1-dioxide (3). Compounds 3 further reacted with 1-(prop-2-yn-1-yl)piperazine in dioxane in presence of triethyl amine to obtain key intermediate 3-(4-(prop-2-yn-1-yl)piperazin-1-yl)-2*H*-benzo[*b*][1,4]thiazine 1,1-dioxide (4).^32^

**Scheme 2:**
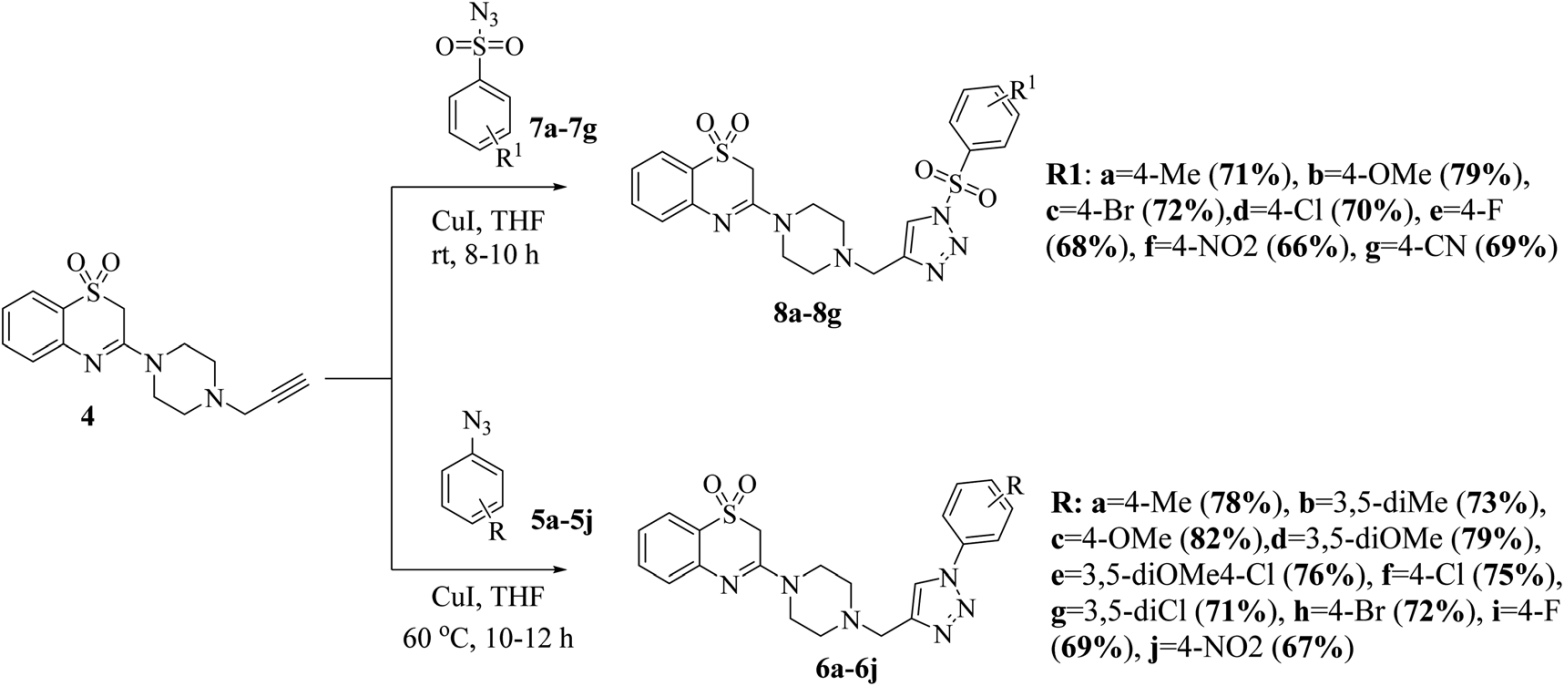
Synthesis of 1,2,3-triazole-piperazin-benzo[*b*][1,4]thiazine 1,1-dioxide (6a–6j & 8a–8g).

The 1,3-dipolar cycloaddition of terminal alkyne 4 with various aryl azides (5a–5j) using a catalytic amount of copper iodide at room temperature provided the corresponding 1,2,3-triazole-piperazin-benzo[*b*][1,4]thiazine 1,1-dioxide (6a–6j) in good to excellent yields.^33^ Similarly, the alkyne 4 with different aryl sulfonyl azides (7a–7g) using catalytic amount of copper iodide at 60 °C temperature for 10–12 h to form corresponding sulfonyl 1,2,3-triazole-piperazin-benzo[*b*][1,4]thiazine 1,1-dioxide (8a–8g) in good to excellent yields (Scheme 2).^27^

### Antibacterial activity

The minimum inhibitory concentration (MIC) of the title compounds (6a–6j & 8a–8g) was determined by using the standard broth microdilution technique against three *Staphylococcus aureus* mutant strains: methicillin-susceptible *S. aureus* (MSSA), methicillin-resistant *S. aureus* (MRSA), and vancomycin-resistant *S. aureus* (VRSA).^24^ The MIC result was displayed as μg mL^−1^ (Table 1). Table 1 shows that among the examined chemicals, 6e, 6f, 6g, 6i, 8d, and 8e had substantial MIC values against all of the mutant strains tested, with MICs ranging from 1.56 to 12.5 μg mL^−1^.

**Table 1:** *In vitro* antibacterial activity data of new compounds (6a–6j & 8a–8g)

Compound	R	Minimum inhibitory concentration^a^ (MIC)		
		MSSA	MRSA	VRSA
6a	4-Me	—	—	—
6b	3,5-diMe	50 ± 1.81	50 ± 1.75	—
6c	4-OMe	25 ± 1.22	25 ± 1.37	—
6d	3,5-diOMe	12.5 ± 1.42	12.5 ± 1.64	25 ± 1.22
6e	3,5-diOMe, 4-Cl	3.12 ± 0.48	6.25 ± 1.88	12.5 ± 1.75
6f	4-Cl	6.25 ± 1.66	12.5 ± 1.86	6.25 ± 1.27
6g	3,5-diCl	3.12 ± 0.52	6.25 ± 1.37	6.25 ± 1.44
6h	4-Br	12.5 ± 1.15	25 ± 1.12	25 ± 1.54
6i	4-F	1.56 ± 0.32	3.12 ± 0.46	6.25 ± 1.12
6j	4-NO_2_	25 ± 1.66	50 ± 1.78	50 ± 1.82
8a	4-Me	25 ± 1.87	25 ± 1.45	—
8b	4-OMe	12.5 ± 1.25	25 ± 1.39	25 ± 1.71
8c	4-Br	6.25 ± 1.24	12.5 ± 1.15	25 ± 1.51
8d	4-Cl	1.56 ± 0.56	3.12 ± 0.32	6.25 ± 0.63
8e	4 F	1.56 ± 0.22	1.56 ± 0.35	3.12 ± 0.51
8f	4-NO_2_	12.5 ± 0.92	25 ± 1.12	25 ± 1.02
8g	4-CN	12.5 ± 0.88	25 ± 1.10	25 ± 1.11
Standard	Dicloxacillin	3.12 ± 0.12	6.25 ± 0.24	6.25 ± 0.41

a MIC: *i.e*., the lowest concentration of the test compound to inhibit the growth of bacteria. Values are expressed as mean ± SD, “—” indicates concentration > 50 μg mL^−1^.

Among the tested series, compound 8e, which contains a 4-fluorophenyl sulfonyl group on the 1,2,3-triazole ring, showed significantly superior inhibiting activity than the standard dicloxacillin against three tested strains, *S. aureus* (MSSA) (1.56 μg mL^−1^) ≈ 2 fold more potent as compared to the standard (3.12 μg mL^−1^), *S. aureus* (MRSA) (1.56 μg mL^−1^) ≈ 4 fold more potent as compared to the standard (6.25 μg mL^−1^), and *S. aureus* (VRSA) (3.12 μg mL^−1^) ≈ 2 fold more potent as compared to the standard (6.25 μg mL^−1^). Also, compounds 8d (4-chlorophenyl sulfonyl group on the 1,2,3-triazole) and 6i (4-fluorophenyl group on the 1,2,3-triazole) showed significantly greater inhibiting activity than the standard against *S. aureus* (MSSA) (1.56 μg mL^−1^) ≈ 2 fold more potent, *S. aureus* (MRSA) (3.12 μg mL^−1^) ≈ 2 fold more potent as compared to the standard (6.25 μg mL^−1^), and equipotent activity against *S. aureus* (VRSA) (6.25 μg mL^−1^) as compared to the standard. Compound 6e (3,5-dichloro phenyl on triazole) have demonstrated equipotent activity against both MSSA (MIC = 3.12 μg mL^−1^) and MRSA (MIC = 6.25 μg mL^−1^) and good activity against VRSA (MIC = 12.5 μg mL^−1^) as compared to the standard dicloxacillin.

Similarly, compound 6f (4-chlorophenyl group on the 1,2,3-triazole) have shown good activity against MSSA (MIC = 6.25 μg mL^−1^) and MRSA (MIC = 12.5 μg mL^−1^), and equipotent activity against VRSA (MIC = 6.25 μg mL^−1^) as compared to the standard. Remaining compounds are moderate to poor activity to poor activity with MIC values ranging from 6.25 to 50 μg mL^−1^.

### Hemolytic activity

Antimicrobial drug hemolytic activity is a crucial factor in determining how safe they are and whether they have the potential to be used in therapeutic settings.^34^ The hemolytic assay was used in the current investigation to evaluate how the synthesized compounds affected red blood cells (RBCs). The hemolytic activities of the different concentations (0.75 μg mL^−1^ to 100 μg mL^−1^) of 6i, 6e, 6f, 6g, 8e, 8d was determined a using human red blood carpuscles (RBCs). The hemolytic percentage of hemolysis was observed and recorded at each concentration of the test compound.

**Fig. 2:**
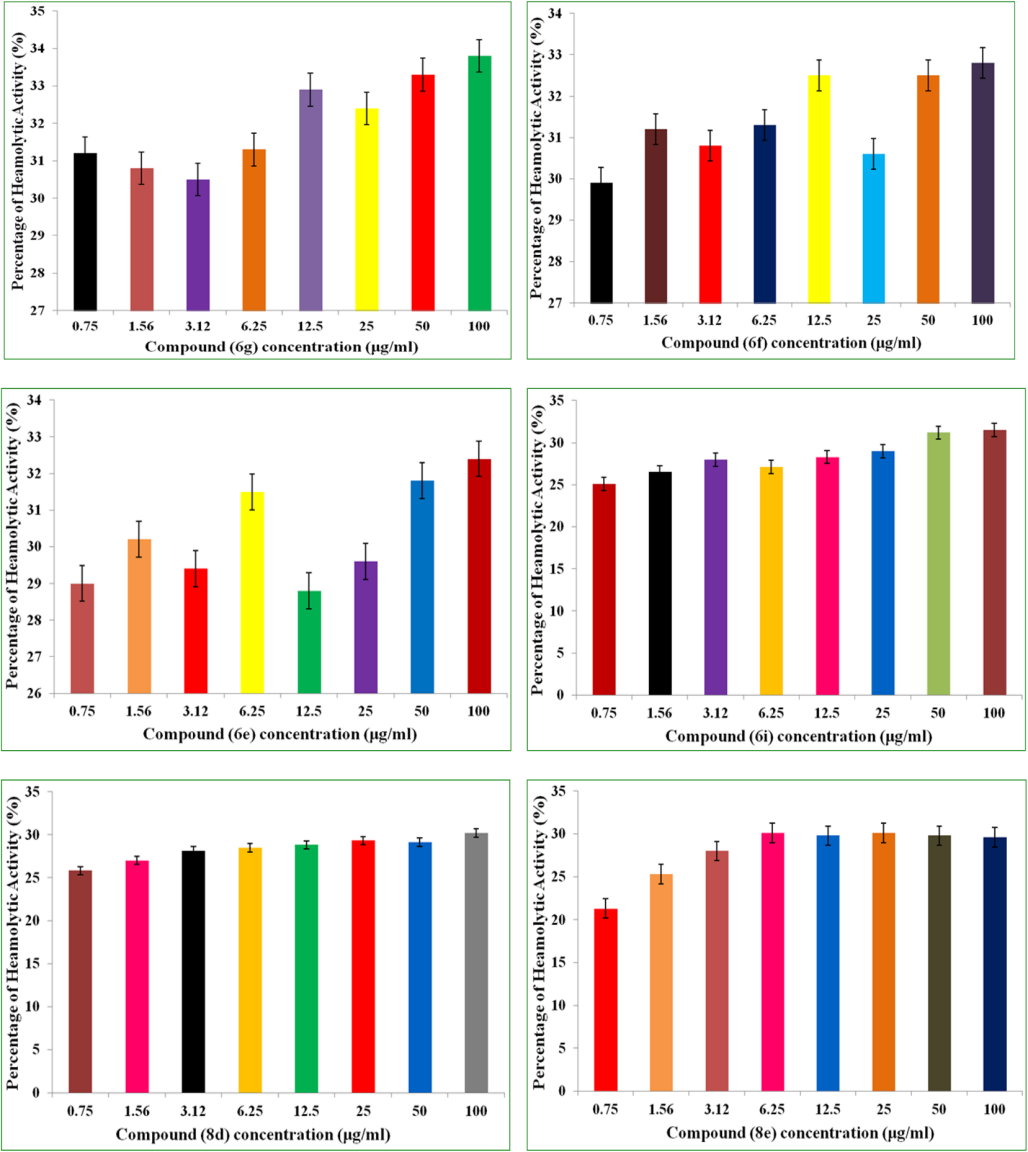
The percentage of hemolysis of the tested compounds (6i, 6e, 6f, 6g, 8e & 8d).

The percentage of hemolysis was ranged grom 21.3 μg mL^−1^ to 33.8 μg mL^−1^. Out of six compounds (6i, 6e, 6f, 6g, 8e, 8d) tested compound 8e and 8d displayed minimal or negligible hemolytic activity across all the tested concentrations 29.6% and 30.2% recorded at 100 μg mL^−1^ concentration respectively. Following, compound 6i and 6e are also exhibited minimal hemolysis percentage 31.5% and 32.4% recorded at 100 μg mL^−1^ concentration respectively. Comparing to hemolytic percentage of compounds 8e, 8d, 6i and 6e, the hemolytic percentage of compounds 6f and 6g were found grater with 32.8% and 33.8% recorded at 100 μg mL^−1^ concentrations respectively. The percentage of hemolysis of the tested compounds was represented in Fig. 2.

### Molecular docking studies

*In silico* docking simulation studies to evaluate the molecular interactions of 6e, 6f, 6g, 6i, 8d, 8e compounds were done with the Human, Mouse and Bovine TLR4 proteins with PDB ID's 3FXI, 3VQ1, 3RG1 respectively. Based on the docking simulations conducted on Human, Mouse and Bovine TLR4 proteins, it is evident that compounds 6i, 8d, 9c, and 8d exhibited superior binding affinity and interaction profiles compared to other compounds analyzed in the current *in silico* study. Compounds 6e, 6f, 6g, 6i, 8d, and 8e displayed affinity with high binding energy towards the human TLR4 protein compared to mouse and bovine TLR4 proteins (Table 2).

**Table 2:** Molecular interaction summary of top compound with different TLR4 proteins

Compounds	Target protein	B.E (kcal mol^−1^)	Interacting amino acids	Nature of interactions
8d	Human TLR4	−9.0	SER183, SER184, LYS230, ASP181, HIS179, LEU212, SER207, PHE263	H-bond, π–π T-shaped, π–alkyl, π–cation, π–anion, π–sulfur, van der Waals
8e	Human TLR4	−8.7	SER183, SER184, LYS230, ASP181, HIS179, LEU212, PHE263, SER207	H-bond, π–π T-shaped, π–alkyl, π–cation, π–anion, π–sulfur, van der Waals
6i	Human TLR4	−8.5	PHE144, ILE146, GLY147, LEU138, LEU117	H-bond, π–sigma, π–alkyl, van der Waals

**Fig. 3:**
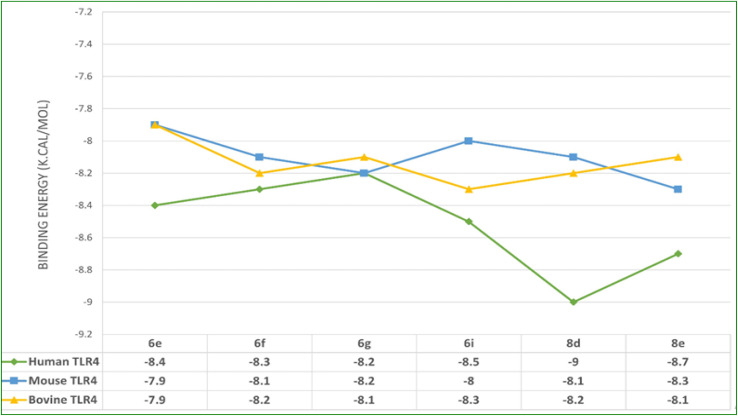
Binding affinity plot of 6e, 6f, 6g, 6i, 8d, and 8e compounds with Human, Mouse and Bovine TLR4 proteins.

**Fig. 4:**
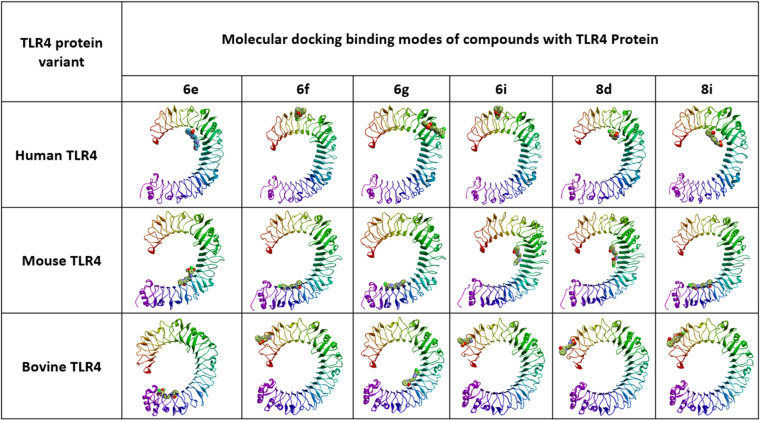
Molecular binding modes of compounds with TLR4 proteins.

**Fig. 5:**
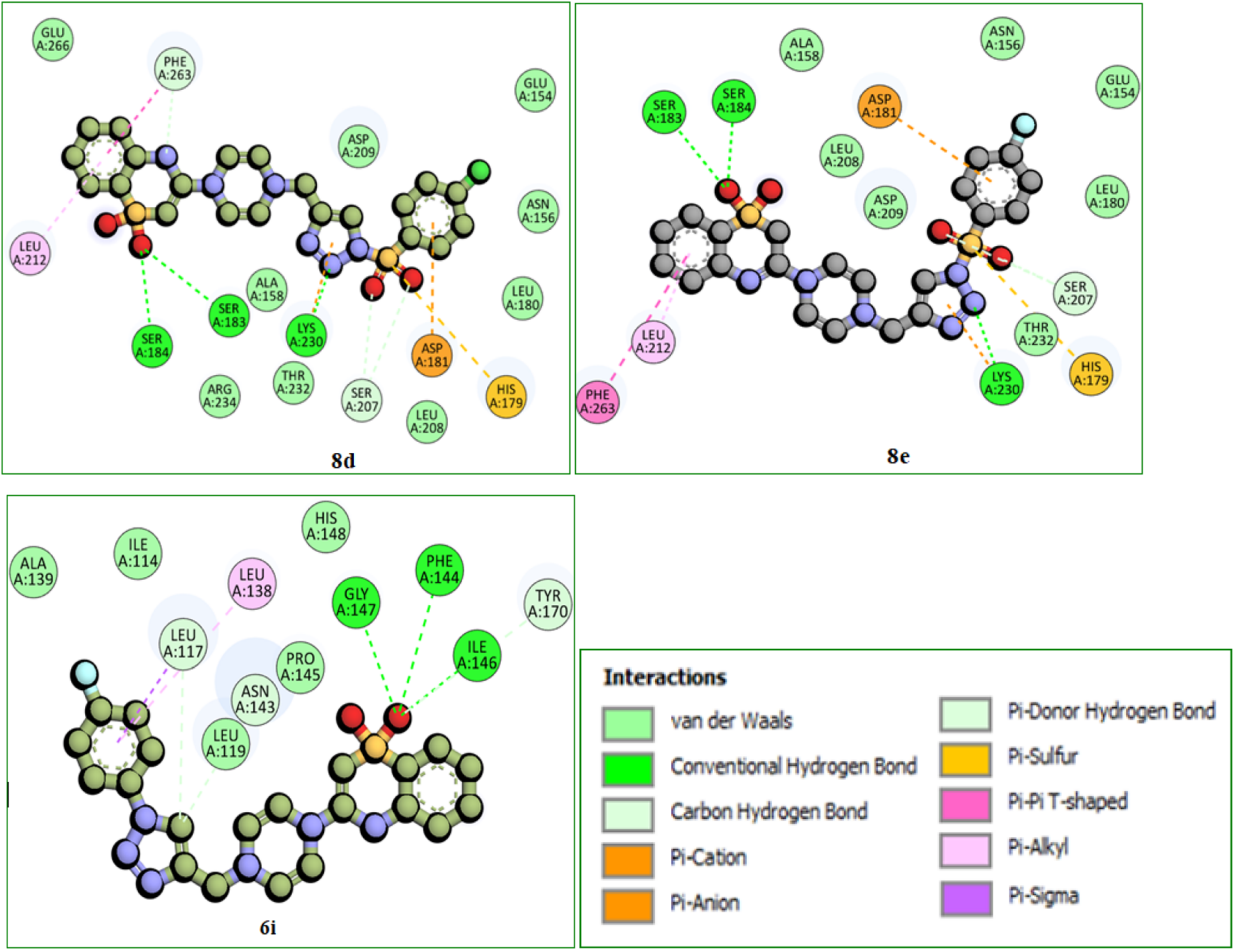
2D molecular interactions of 8d, 8e, and 6i compounds with the amino acid residues of the human TLR4 protein. Interactions were displayed as color coded dashed lines; green lines indicated the H-bonds.

**Fig. 6:**
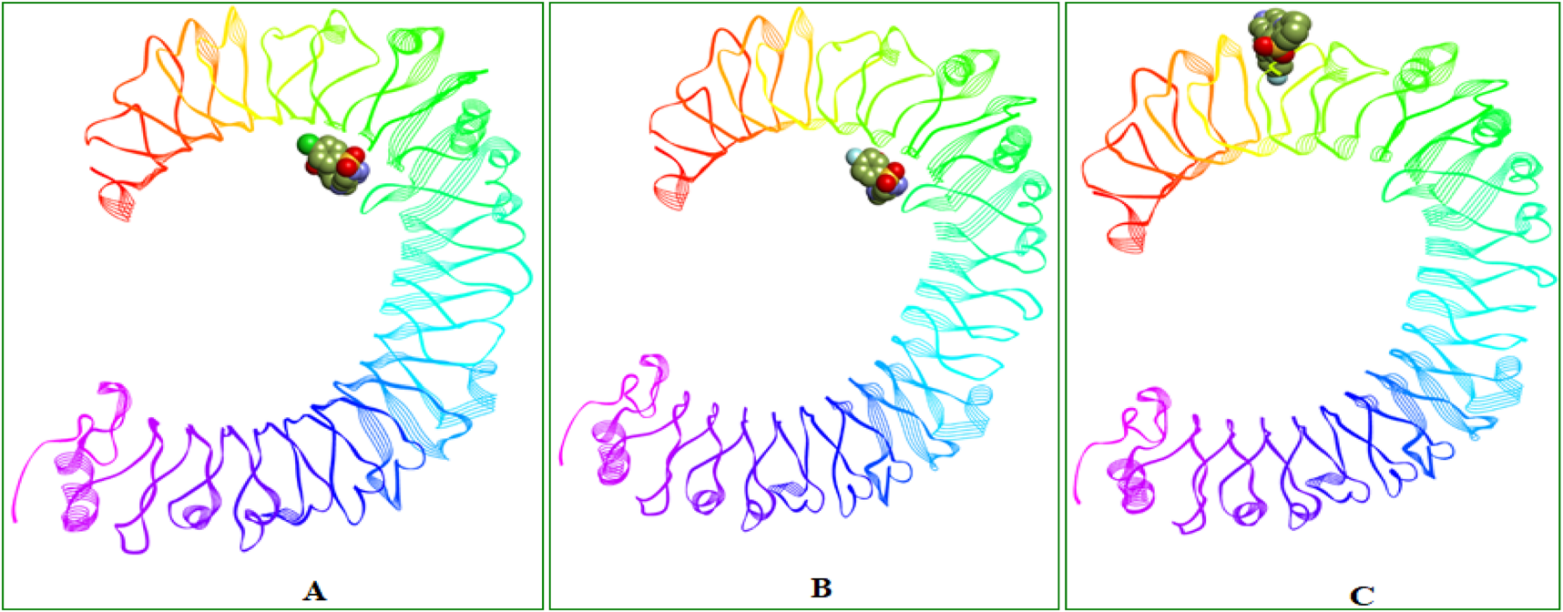
Binding site and 3D representation of the binding orientations of (A) 8d, (B) 8e and (C) 6i compounds in Human TLR4 protein.

Among all, the 8d compound is displaying highest binding energy (−9.0 Kcal mol^−1^) with human TLR4 protein. 8d and 8e compounds which are structurally similar analogs with variation only in the halogen group on the 4th position of the phenyl ring, (–Cl (8d) and –F (8e)) had a very similar interaction profile. They both had three H-bonds with SER183 (8d: 4.29 Å, 8e: 4.04 Å), SER184 (8d: 4.01 Å, 8e: 4.09 Å) and LYS230 (8d: 4.26 Å, 8e: 4.46 Å), residues where the benzothiazine –S=O group is contributing two H-bond interactions with SER183 and SER184 and the triazole nitrogen had another hydrogen bond interaction with LYS230. Pi-cation and pi-anion interactions were exhibited by LYS230 and ASP181 whereas the HIS179 had pi-sulfur interaction with the sulfonyl group which is linking triazole and 4-Chloro phenyl groups. Pi–pi T-shaped interactions were displayed by PHE263 residue with the aromatic ring of benzothiazine 1,1 dioxide ring and the LEU212 had pi-alkyl interactions with the same aromatic ring and the surrounding binding site residues in close proximity had Vander Waals interactions. 6i compound had three H-bonds with PHE144 (6.64 Å), ILE146 (4.97 Å), GLY147 (3.66 Å) residues through the benzothiazole –S=O group and it also displays other non-hydrogen bonded interactions like pi-sigma interactions with LEU117 and pi-alkyl interactions with LEU138 residues with the fluro substituted aromatic phenyl group. 6i compound had a distinct binding site in human TLR4 compared with 8d and 8e compounds. 8d and 8e are buried deep inside the pocket whereas the 6i is binding to the external the surface of human TLR4 protein (Table 2). Molecular interactions, binding site and the binding pose of 8d, 8e and 6i were represented in the Fig. 3–6 respectively.

### ADME properties

SWISS ADME webserver^35^ (http://www.swissadme.ch/) had been used for the evaluation of physico-chemical, metabolism and drug likeliness properties of compounds which are found to be promising in the *in vitro* and *in silico* assessments.

In summary, the overall ADME predictions indicate that the compounds 8d, 8e and 6i a favorable pharmacokinetic profile (Table 3). They will have high gastrointestinal (GI) absorption and none of the top compounds were predicted to permeate the blood–brain barrier (BBB) and might act as substrates for P-glycoprotein (P-gp). All of the top compounds investigated in this study adhered to Lipinski's rule of five with slight deviation in molecular weight (8d and 8e). Furthermore, the compounds were also predicted to have the potential to inhibit cytochrome P450 isoforms of CYP2C9, CYP2C19 and CYP3A4.

**Table 3:** Physico-chemical properties and drug-likeness prediction of compounds with better binding energy and interaction profile using SWISS ADME

Parameters	8d	8e	6i
Molecular weight (g mol^−1^)	521.01	504.56	440.49
log *P* o/w	2.24	2.01	2.30
No. of. H-bond donors	0	0	0
No. of H-bond acceptors	8	9	7
Solubility	Soluble	Soluble	Soluble
TPSA (Å^2^)	134.59	134.59	92.07
GI absorption	High	High	High
BBB permeation	No	No	No
P-gp substrate	Yes	Yes	Yes
Drug likeness (Lipinski)	Yes	Yes	Yes
Bioavailability score	0.55	0.55	0.55
CYP450 isoforms inhibition	CYP2C9, CYP3A4	CYP2C9, CYP3A4	CYP2C19, CYP2C9, CYP3A4

## Conclusion

In the present work, a series of novel 1,2,3-triazole-piperazin-benzo[*b*][1,4]thiazine 1,1-dioxide derivatives (6a–6j and 8a–8g) and investigated them *in vitro* antibacterial against methicillin-susceptible *S. aureus* (MSSA), methicillin-resistant *S. aureus* (MRSA), and vancomycin-resistant *S. aureus* (VRSA). Among them, the compounds 6e, 6g, 6i, 8d, and 8e exhibited excellent antibacterial activity against with MIC values of 1.56 ± 0.22 to 12.5 ± 1.75 μg mL^−1^, the most potent compounds (6i, 6e, 6f, 6g, 8e, 8d) were tested hemolytic activity and the compounds 8e and 8d displayed minimal or negligible hemolytic activity across all the tested concentrations 29.6% and 30.2% recorded at 100 μg mL^−1^ concentration respectively. *In silico* docking studies were performed to evaluate the molecular interactions of most potent compounds against Human, Mouse and Bovine TLR4 proteins and observed that three of the compounds 6i, 8d, and 8i had appreciable binding energies ranging −8.5 to −9.0 Kcal mol^−1^. Remarkably, the *in silico* ADME of more potent compounds 6i, 8d, and 8e were also found to be covenant with the corresponding *in vitro* activity.

## Experimental

All the commercially available chemicals were utilized without of further purification. The purity of compounds was analyzed on Merck 60F254 silica gel TLC plates. Melting points were recorded on a hot stage melting point apparatus in Ernst Leitz Wetzlar, Germany, and were uncorrected. The ^1^H and ^13^C NMR spectra were recorded using the Mercuryplus spectrometer (operating at 500 MHz for ^1^H and 126 MHz for ^13^C), and the chemical shifts were referenced to TMS. The ESI (electrospray ionization) mass spectra at an ionising voltage of 70 eV were obtained with the help of a Shimadzu QP5050A quadrupole-based mass spectrometer. Elemental analyses were obtained with an Elemental Analyser PerkinElmer 240 C apparatus.

### Synthesis of 3-(4-(prop-2-yn-1-yl)piperazin-1-yl)-2*H*-benzo[*b*][1,4]thiazine 1,1-dioxide (4)

To a mixture of 3-chloro-2*H*-benzo[*b*][1,4]thiazine 1,1-dioxide (3) (0.012 mol) with TEA (0.035 mol) in dioxane (30 mL) was added 1-(prop-2-yn-1-yl)piperazine (0.013 mol). The reaction mixture was heated at 80 °C for 8 h. The volatiles were removed under reduced pressure. The residue was diluted with CHCl_3_, washed with water and brine, and dried over Na_2_SO_4_. Upon removing the volatiles under reduced pressure, the crude residue was purified by chromatography on silica gel (cyclohexane–EtOAc = 8 : 2) yielding compound 4 as a pale red solid, yield 77%. mp 112–114 °C. ^1^H-NMR (500 MHz, CDCl_3_) *δ* 7.97 (d, *J* = 8.0 Hz, 1H), 7.69 (d, *J* = 8.0 Hz, 1H), 7.64–7.60 (m, 1H), 7.44–7.40 (m, 1H), 4.25 (s, 2H, SO_2_–CH_2_), 3.89 (d, *J* = 4.0 Hz, 2H, N–CH_2_), 3.76 (t, *J* = 4.0 Hz, 4H, 2N–CH_2_), 3.51 (t, *J* = 4.0 Hz, 4H, 2N–CH_2_), 2.22 (t, *J* = 4.0 Hz, 1H, –CH); ^13^C-NMR (126 MHz, CDCl_3_): *δ* 153.27, 146.52, 133.52, 132.46, 129.25, 124.65, 122.51, 73.30, 72.62, 59.10, 46.33(2C), 44.16(2C), 42.26; HRMS (*m*/*z*): Cal for C_15_H_17_N_3_O_2_S: [M + H]^+^*m*/*z*: 304.1041, found: 304.1043.

### General procedure for the synthesis of 1,2,3-triazole-piperazin-benzo[*b*][1,4]thiazine 1,1-dioxide (6a–6j)

To a stirred solution of alkyne (4) (1.5 mmol) and aryl azide (2.0 mmol) in THF (15 mL) was added CuI (10 mol%) and the reaction mixture was stirred at room temperature for 8–10 h. After completion of the reaction, the reaction mixture was diluted with water (15 mL) and the product was extracted with ethyl acetate (2 × 15 mL). The combined organic layer was washed with brine and dried over anhydrous Na_2_SO_4_. After filtration, the solvent was evaporated under vacuum and the crude product obtained was purified by column chromatography (hexane/ethyl acetate gradient) to afford the compounds 6a–6j.

#### 3-(4-((1-(*p*-Tolyl)-1*H*-1,2,3-triazol-4-yl)methyl)piperazin-1-yl)-2*H*-benzo[*b*][1,4]thiazine 1,1-dioxide (6a)

Pale red solid; M.p: 116–128 °C; ^1^H-NMR (500 MHz, CDCl_3_) *δ* 8.03 (s, 1H, tri-H), 7.91 (d, *J* = 8.0 Hz, 1H), 7.72 (d, *J* = 8.0 Hz, 1H), 7.66 (d, *J* = 8.0 Hz, 2H), 7.61–7.57 (m, 1H), 7.46 (d, *J* = 8.0 Hz, 2H), 7.41–7.37 (m, 1H), 5.25 (s, 2H, N–CH_2_), 4.31 (s, 2H, SO_2_–CH_2_), 3.77 (t, *J* = 4.0 Hz, 4H, 2N–CH_2_), 3.55 (t, *J* = 4.0 Hz, 4H, 2N–CH_2_), 2.31 (s, 3H, –CH_3_). ^13^C-NMR (126 MHz, CDCl_3_): *δ* 153.36, 146.58, 143.09, 137.08, 136.32, 133.30, 132.29, 129.02, 128.50(2C), 124.50(2C), 123.07, 122.29, 121.05, 59.52, 49.75, 46.23(2C), 44.73(2C), 21.66. ESI-MS: 437.16 [M + H]. Anal. calcd for C_22_H_24_N_6_O_2_S: C, 60.53; H, 5.54; N, 19.25. Found: C, 60.56; H, 5.56; N, 19.22.

#### 3-(4-((1-(3,5-Dimethylphenyl)-1*H*-1,2,3-triazol-4-yl)methyl)piperazin-1-yl)-2*H*-benzo[*b*][1,4]thiazine 1,1-dioxide (6b)

Pale red solid; M.p: 127–129 °C; ^1^H-NMR (500 MHz, CDCl_3_) *δ* 8.05 (s, 1H, tri-H), 7.88 (d, *J* = 8.0 Hz, 1H), 7.70 (d, *J* = 8.0 Hz, 1H), 7.62–7.57 (m, 1H), 7.53 (s, 1H), 7.40–7.35 (m, 1H), 7.29 (s, 2H), 5.24 (s, 2H, N–CH_2_), 4.31 (s, 2H, SO_2_–CH_2_), 3.76 (t, *J* = 4.0 Hz, 4H, 2N–CH_2_), 3.57 (t, *J* = 4.0 Hz, 4H, 2N–CH_2_), 2.37 (s, 6H, 2-CH_3_). ^13^C-NMR (126 MHz, CDCl_3_): *δ* 153.20, 146.54, 143.28, 139.84(2C), 137.40, 133.55, 132.91, 129.61, 126.37, 125.14(2C), 124.04, 122.96, 122.51, 59.51, 49.42, 46.76(2C), 44.31(2C), 21.17(2C). ESI-MS: 451.18 [M + H]. Anal. calcd for C_23_H_26_N_6_O_2_S: C, 61.31; H, 5.82; N, 18.65. Found: C, 61.35; H, 5.86; N, 18.61.

#### 3-(4-((1-(4-Methoxyphenyl)-1*H*-1,2,3-triazol-4-yl)methyl)piperazin-1-yl)-2*H*-benzo[*b*][1,4]thiazine 1,1-dioxide (6c)

Red solid; M.p: 123–125 °C; ^1^H-NMR (500 MHz, CDCl_3_) *δ* 8.05 (s, 1H, tri-H), 7.93 (d, *J* = 8.0 Hz, 1H), 7.77 (d, *J* = 8.0 Hz, 2H), 7.70 (d, *J* = 8.0 Hz, 1H), 7.63–7.59 (m, 1H), 7.41–7.36 (m, 1H), 7.00 (d, *J* = 8.0 Hz, 2H), 5.26 (s, 2H, N–CH_2_), 4.29 (s, 2H, SO_2_–CH_2_), 3.85 (s, 3H, OCH_3_), 3.77 (t, *J* = 4.0 Hz, 4H, 2N–CH_2_), 3.56 (t, *J* = 4.0 Hz, 4H, 2N–CH_2_). ^13^C-NMR (126 MHz, CDCl_3_): *δ* 159.67, 153.43, 146.25, 143.12, 133.46, 132.36, 131.55, 129.42, 124.34, 123.53(2C), 122.51, 121.53, 114.81(2C), 59.46, 56.24, 49.46, 46.48(2C), 44.07(2C). ESI-MS: 453.16 [M + H]. Anal. calcd for C_22_H_24_N_6_O_3_S: C, 58.39; H, 5.35; N, 18.57. Found: C, 58.42; H, 5.33; N, 18.55.

#### 3-(4-((1-(3,5-Dimethoxyphenyl)-1*H*-1,2,3-triazol-4-yl)methyl)piperazin-1-yl)-2*H*-benzo[*b*][1,4]thiazine 1,1-dioxide (6d)

Pale red solid; M.p: 134–136 °C; ^1^H-NMR (500 MHz, CDCl_3_) *δ* 8.06 (s, 1H, tri-H), 7.93 (d, *J* = 8.0 Hz, 1H), 7.72 (d, *J* = 8.0 Hz, 1H), 7.63–7.56 (m, 1H), 7.50 (s, 2H), 7.41–7.36 (m, 1H), 6.99 (s, 1H), 5.26 (s, 2H, N–CH_2_), 4.31 (s, 2H, SO_2_–CH_2_), 3.85 (s, 6H, 2OCH_3_), 3.75 (t, *J* = 4.0 Hz, 4H, 2N–CH_2_), 3.52 (t, *J* = 4.0 Hz, 4H, 2N–CH_2_). ^13^C-NMR (126 MHz, CDCl_3_): *δ* 159.06(2C), 153.31, 146.61, 143.10, 140.22, 133.23, 132.56, 129.84, 124.45, 122.82, 122.14, 110.94(2C), 106.05, 59.21, 56.17(2C), 49.13, 46.65(2C), 44.52(2C). ESI-MS: 483.17 [M + H]. Anal. calcd for C_23_H_26_N_6_O_4_S: C, 57.25; H, 5.43; N, 17.42. Found: C, 57.28; H, 5.47; N, 17.39.

#### 3-(4-((1-(4-Chloro-3,5-dimethoxyphenyl)-1*H*-1,2,3-triazol-4-yl)methyl)piperazin-1-yl)-2*H*-benzo[*b*][1,4]thiazine 1,1-dioxide (6e)

Pale yellow solid; M.p: 141–143 °C; ^1^H-NMR (500 MHz, CDCl_3_) *δ* 8.08 (s, 1H, tri-H), 7.91 (d, *J* = 8.0 Hz, 1H), 7.69 (d, *J* = 8.0 Hz, 1H), 7.61–7.55 (m, 1H), 7.45 (s, 2H), 7.40–7.34 (m, 1H), 5.24 (s, 2H, N–CH_2_), 4.31 (s, 2H, SO_2_–CH_2_), 3.86 (s, 6H, 2OCH_3_), 3.75 (t, *J* = 4.0 Hz, 4H, 2N–CH_2_), 3.52 (t, *J* = 4.0 Hz, 4H, 2N–CH_2_). ^13^C-NMR (126 MHz, CDCl_3_): *δ* 159.12(2C), 153.26, 146.30, 143.02, 138.39, 133.50, 132.03, 129.53, 124.87, 122.87, 122.18, 118.02, 112.45(2C), 59.47, 56.49(2C), 49.15, 46.58(2C), 44.36(2C). ESI-MS: 517.13 [M + H]. Anal. calcd for C_23_H_25_ClN_6_O_4_S: C, 53.43; H, 4.87; N, 16.26. Found: C, 53.45; H, 4.90; N, 16.22.

#### 3-(4-((1-(4-Chlorophenyl)-1*H*-1,2,3-triazol-4-yl)methyl)piperazin-1-yl)-2*H*-benzo[*b*][1,4]thiazine 1,1-dioxide (6f)

Pale yellow solid; M.p: 120–122 °C; ^1^H-NMR (500 MHz, CDCl_3_) *δ* 8.06 (s, 1H, tri-H), 7.91 (d, *J* = 8.0 Hz, 1H), 7.77 (d, *J* = 8.0 Hz, 2H), 7.72 (d, *J* = 8.0 Hz, 1H), 7.60–7.55 (m, 1H), 7.48 (d, *J* = 8.0 Hz, 2H), 7.38–7.34 (m, 1H), 5.27 (s, 2H, N–CH_2_), 4.33 (s, 2H, SO_2_–CH_2_), 3.78 (t, *J* = 4.0 Hz, 4H, 2N–CH_2_), 3.56 (t, *J* = 4.0 Hz, 4H, 2N–CH_2_). ^13^C-NMR (126 MHz, CDCl_3_): *δ* 153.35, 146.46, 143.03, 136.38, 133.12, 132.29, 130.86, 129.87, 128.72(2C), 124.14, 122.85, 122.10(2C), 121.00, 59.68, 49.54, 46.39(2C), 44.30(2C). ESI-MS: 457.11 [M + H]. Anal. calcd for C_21_H_21_ClN_6_O_2_S: C, 55.20; H, 4.63; N, 18.39. Found: C, 55.23; H, 4.60; N, 18.36.

#### 3-(4-((1-(3,5-Dichlorophenyl)-1*H*-1,2,3-triazol-4-yl)methyl)piperazin-1-yl)-2*H*-benzo[*b*][1,4]thiazine 1,1-dioxide (6g)

Pale yellow solid; M.p: 130–132 °C; ^1^H-NMR (500 MHz, CDCl_3_) *δ* 8.10 (s, 1H, tri-H), 7.91 (d, *J* = 8.0 Hz, 1H), 7.73 (d, *J* = 8.0 Hz, 1H), 7.69 (s, 2H), 7.61–7.57 (m, 1H), 7.44–7.41 (m, 1H), 7.32 (s, 1H), 5.26 (s, 2H, N–CH_2_), 4.31 (s, 2H, SO_2_–CH_2_), 3.79 (t, *J* = 4.0 Hz, 4H, 2N–CH_2_), 3.56 (t, *J* = 4.0 Hz, 4H, 2N–CH_2_). ^13^C-NMR (126 MHz, CDCl_3_): *δ* 153.54, 146.49, 143.64, 139.63, 135.40(2C), 133.39, 132.49, 129.41, 124.50, 123.90, 122.80, 122.10(2C), 120.14, 59.22, 49.12, 46.55(2C), 44.33(2C). ESI-MS: 491.07 [M + H]. Anal. calcd for C_21_H_20_Cl_2_N_6_O_2_S: C, 51.33; H, 4.10; N, 17.10. Found: C, 51.29; H, 4.13; N, 17.07.

#### 3-(4-((1-(4-Bromophenyl)-1*H*-1,2,3-triazol-4-yl)methyl)piperazin-1-yl)-2*H*-benzo[*b*][1,4]thiazine 1,1-dioxide (6h)

White solid; M.p: 144–146 °C; ^1^H-NMR (500 MHz, CDCl_3_) *δ* 8.03 (s, 1H, tri-H), 7.91 (d, *J* = 8.0 Hz, 1H), 7.70 (d, *J* = 8.0 Hz, 1H), 7.65 (d, *J* = 8.0 Hz, 2H), 7.60–7.54 (m, 1H), 7.45 (d, *J* = 8.0 Hz, 2H), 7.38–7.33 (m, 1H), 5.24 (s, 2H, N–CH_2_), 4.32 (s, 2H, SO_2_–CH_2_), 3.76 (t, *J* = 4.0 Hz, 4H, 2N–CH_2_), 3.56 (t, *J* = 4.0 Hz, 4H, 2N–CH_2_). ^13^C-NMR (126 MHz, CDCl_3_): *δ* 153.25, 146.71, 143.65, 135.46, 133.22, 132.40, 131.50(2C), 129.37, 124.06, 122.81, 122.08, 121.30(2C), 120.25, 59.23, 49.39, 46.73(2C), 44.51(2C). Anal. calcd for C_21_H_21_BrN_6_O_2_S: C, 50.30; H, 4.22; N, 16.76. Found: C, 50.26; H, 4.26; N, 16.72. HRMS (*m*/*z*): Cal for C_21_H_21_BrN_6_O_2_S: [M + H]^+^*m*/*z*: 501.0630, found: 501.0636.

#### 3-(4-((1-(4-Fluorophenyl)-1*H*-1,2,3-triazol-4-yl)methyl)piperazin-1-yl)-2*H*-benzo[*b*][1,4]thiazine 1,1-dioxide (6i)

Red solid; M.p: 129–131 °C; ^1^H-NMR (500 MHz, CDCl_3_) *δ* 8.23 (d, *J* = 8.0 Hz, 2H), 8.10 (s, 1H, tri-H), 8.00 (d, *J* = 8.0 Hz, 2H), 7.91 (d, *J* = 8.0 Hz, 1H), 7.72 (d, *J* = 8.0 Hz, 1H), 7.65–7.61 (m, 1H), 7.44–7.41 (m, 1H), 5.29 (s, 2H, N–CH_2_), 4.35 (s, 2H, SO_2_–CH_2_), 3.78 (t, *J* = 4.0 Hz, 4H, 2N–CH_2_), 3.56 (t, *J* = 4.0 Hz, 4H, 2N–CH_2_). ^13^C-NMR (126 MHz, CDCl_3_): *δ* 160.27, 158.85, 153.35, 146.48, 143.30, 133.59, 133.07, 132.50, 129.67, 125.36, 125.25, 123.86(2C), 122.93, 122.32, 117.93, 117.58, 59.23, 49.50, 45.62(2C), 44.14(2C). Anal. calcd for C_21_H_21_FN_6_O_2_S: C, 57.26; H, 4.81; N, 19.08. Found: C, 57.21; H, 4.84; N, 19.05. HRMS (*m*/*z*): Cal for C_21_H_21_FN_6_O_2_S: [M + H]^+^*m*/*z*: 441.1431, found: 441.1435.

#### 3-(4-((1-(4-Nitrophenyl)-1*H*-1,2,3-triazol-4-yl)methyl)piperazin-1-yl)-2*H*-benzo[*b*][1,4]thiazine 1,1-dioxide (6j)

Yellow solid; M.p: 149–151 °C; ^1^H-NMR (500 MHz, CDCl_3_) *δ* 8.43 (d, *J* = 8.0 Hz, 2H), 8.29 (d, *J* = 8.0 Hz, 2H), 8.11 (s, 1H, tri-H), 7.93 (d, *J* = 8.0 Hz, 1H), 7.75 (d, *J* = 8.0 Hz, 1H), 7.65–7.61 (m, 1H), 7.44–7.40 (m, 1H), 5.28 (s, 2H, N–CH_2_), 4.35 (s, 2H, SO_2_–CH_2_), 3.78 (t, *J* = 4.0 Hz, 4H, 2N–CH_2_), 3.56 (t, *J* = 4.0 Hz, 4H, 2N–CH_2_). ^13^C-NMR (126 MHz, CDCl_3_): *δ* 153.42, 146.73, 145.77, 143.15, 140.30, 133.31, 132.02, 129.50, 126.78(2C), 124.45, 122.81(2C), 122.26, 121.62, 59.39, 49.25, 46.72(2C), 44.28(2C). ESI-MS: 468.13 [M + H]. Anal. calcd for C_21_H_21_N_7_O_4_S: C, 53.95; H, 4.53; N, 20.97. Found: C, 53.91; H, 4.50; N, 20.99.

### General procedure for the synthesis of sulfonyl 1,2,3-triazole-piperazin-benzo[*b*][1,4]thiazine 1,1-dioxide (8a–8g)

To a stirred solution of alkyne (4) (1.5 mmol) and aryl sulfonyl azide (2.0 mmol) in THF (15 mL) was added CuI (10 mol%) and the reaction mixture was stirred at 60 °C temperature for 10–12 h. After completion of the reaction, the reaction mixture was diluted with water (15 mL) and the product was extracted with ethyl acetate (2 × 15 mL). The combined organic layer was washed with brine and dried over anhydrous Na_2_SO_4_. After filtration, the solvent was evaporated under vacuum and the crude product obtained was purified by column chromatography (hexane/ethyl acetate gradient) to afford the compounds 8a–8g.

#### 3-(4-((1-Tosyl-1*H*-1,2,3-triazol-4-yl)methyl)piperazin-1-yl)-2*H*-benzo[*b*][1,4]thiazine 1,1-dioxide (8a)

White solid; M.p: 153–155 °C; ^1^H-NMR (500 MHz, CDCl_3_) *δ* 8.03 (s, 1H, tri-H), 7.91 (d, *J* = 8.0 Hz, 1H), 7.76 (d, *J* = 8.0 Hz, 2H), 7.70 (d, *J* = 8.0 Hz, 1H), 7.62–7.56 (m, 1H), 7.47 (d, *J* = 8.0 Hz, 2H), 7.39–7.35 (m, 1H), 5.25 (s, 2H, N–CH_2_), 4.36 (s, 2H, SO_2_–CH_2_), 3.78 (t, *J* = 4.0 Hz, 4H, 2N–CH_2_), 3.56 (t, *J* = 4.0 Hz, 4H, 2N–CH_2_), 2.35 (s, 3H, –CH_3_). ^13^C-NMR (126 MHz, CDCl_3_): *δ* 153.54, 146.58, 143.34, 137.52, 135.72, 133.88, 132.28, 130.84, 130.08(2C), 129.02, 128.05(2C), 124.35, 122.63, 59.11, 49.57, 46.46(2C), 44.16(2C), 21.26. ESI-MS: 501.13 [M + H]. Anal. calcd for C_22_H_24_N_6_O_4_S_2_: C, 52.78; H, 4.83; N, 16.79. Found: C, 52.81; H, 4.80; N, 16.75.

#### 3-(4-((1-((4-Methoxyphenyl)sulfonyl)-1*H*-1,2,3-triazol-4-yl)methyl)piperazin-1-yl)-2*H*-benzo[*b*][1,4]thiazine 1,1-dioxide (8b)

Dirty white solid; M.p: 158–160 °C; ^1^H-NMR (500 MHz, CDCl_3_) *δ* 8.01 (s, 1H, tri-H), 7.91 (d, *J* = 8.0 Hz, 1H), 7.78 (d, *J* = 8.0 Hz, 2H), 7.69 (d, *J* = 8.0 Hz, 1H), 7.63–7.59 (m, 1H), 7.44–7.40 (m, 1H), 7.00 (d, *J* = 8.0 Hz, 2H), 5.27 (s, 2H, N–CH_2_), 4.38 (s, 2H, SO_2_–CH_2_), 3.84 (s, 3H, OCH_3_), 3.75 (t, *J* = 4.0 Hz, 4H, 2N–CH_2_), 3.55 (t, *J* = 4.0 Hz, 4H, 2N–CH_2_). ^13^C-NMR (126 MHz, CDCl_3_): *δ* 159.27, 153.07, 146.08, 135.39, 133.52, 132.91, 131.37, 130.19(2C), 129.67, 128.24, 124.04, 123.30, 116.04(2C), 59.06, 56.29, 49.25, 46.22(2C), 44.36(2C). ESI-MS: 517.12 [M + H]. Anal. calcd for C_22_H_24_N_6_O_5_S_2_: C, 51.15; H, 4.68; N, 16.27. Found: C, 51.18; H, 4.65; N, 16.25.

#### 3-(4-((1-((4-Bromophenyl)sulfonyl)-1*H*-1,2,3-triazol-4-yl)methyl)piperazin-1-yl)-2*H*-benzo[*b*][1,4]thiazine 1,1-dioxide (8c)

White solid; M.p: 166–168 °C; ^1^H-NMR (500 MHz, CDCl_3_) *δ* 8.04 (s, 1H, tri-H), 7.91 (d, *J* = 8.0 Hz, 1H), 7.71 (d, *J* = 8.0 Hz, 1H), 7.68 (d, *J* = 8.0 Hz, 2H), 7.63–7.59 (m, 1H), 7.47 (d, *J* = 8.0 Hz, 2H), 7.42–7.39 (m, 1H), 5.24 (s, 2H, N–CH_2_), 4.36 (s, 2H, SO_2_–CH_2_), 3.75 (t, *J* = 4.0 Hz, 4H, 2N–CH_2_), 3.55 (t, *J* = 4.0 Hz, 4H, 2N–CH_2_). ^13^C-NMR (126 MHz, CDCl_3_): *δ* 153.33, 146.65, 138.88, 135.39, 133.25, 132.51, 132.04(2C), 131.52, 130.09, 129.48(2C), 128.87, 124.37, 122.36, 59.55, 49.57, 46.35(2C), 44.15(2C). ESI-MS: 565.02 [M + H]. Anal. calcd for C_21_H_21_BrN_6_O_4_S_2_: C, 44.60; H, 3.74; N, 14.86. Found: C, 44.63; H, 3.71; N, 14.83.

#### 3-(4-((1-((4-Chlorophenyl)sulfonyl)-1*H*-1,2,3-triazol-4-yl)methyl)piperazin-1-yl)-2*H*-benzo[*b*][1,4]thiazine 1,1-dioxide (8d)

Pale yellow solid; M.p: 153–155 °C; ^1^H-NMR (500 MHz, CDCl_3_) *δ* 8.05 (s, 1H, tri-H), 7.93 (d, *J* = 8.0 Hz, 1H), 7.80 (d, *J* = 8.0 Hz, 2H), 7.71 (d, *J* = 8.0 Hz, 1H), 7.62–7.57 (m, 1H), 7.47 (d, *J* = 8.0 Hz, 2H), 7.40–7.36 (m, 1H), 5.26 (s, 2H, N–CH_2_), 4.37 (s, 2H, SO_2_–CH_2_), 3.77 (t, *J* = 4.0 Hz, 4H, 2N–CH_2_), 3.57 (t, *J* = 4.0 Hz, 4H, 2N–CH_2_). ^13^C-NMR (126 MHz, CDCl_3_): *δ* 153.25, 146.74, 143.26, 138.48, 135.32, 133.27, 132.39, 130.67(2C), 130.13, 129.79 (2C), 129.02, 124.58, 122.37, 59.31, 49.36, 46.85 (2C), 44.18 (2C). Anal. calcd for C_21_H_21_ClN_6_O_4_S_2_: C, 48.41; H, 4.06; N, 16.13. Found: C, 48.38; H, 4.02; N, 16.15. HRMS (*m*/*z*): Cal for C_21_H_21_ClN_6_O_4_S_2_: [M + H]^+^*m*/*z*: 521.0754, found: 521.0752.

#### 3-(4-((1-((4-Fluorophenyl)sulfonyl)-1*H*-1,2,3-triazol-4-yl)methyl)piperazin-1-yl)-2*H*-benzo[*b*][1,4]thiazine 1,1-dioxide (8e)

Pale red solid; M.p: 150–152 °C; ^1^H-NMR (500 MHz, CDCl_3_) *δ* 8.25 (d, *J* = 8.0 Hz, 2H), 8.15 (d, *J* = 8.0 Hz, 2H), 8.08 (s, 1H, tri-H), 7.92 (d, *J* = 8.0 Hz, 1H), 7.72 (d, *J* = 8.0 Hz, 1H), 7.62–7.58 (m, 1H), 7.41–7.37 (m, 1H), 5.29 (s, 2H, N–CH_2_), 4.37 (s, 2H, SO_2_–CH_2_), 3.78 (t, *J* = 4.0 Hz, 4H, 2N–CH_2_), 3.56 (t, *J* = 4.0 Hz, 4H, 2N–CH_2_). ^13^C-NMR (126 MHz, CDCl_3_): *δ* 166.57, 164.87, 153.69, 146.57, 135.05, 134.33, 133.06, 132.06, 130.18 (2C), 129.36, 128.71, 124.59, 122.27, 119.81, 119.45, 59.24, 49.58, 46.22 (2C), 44.57 (2C). Anal. calcd for C_21_H_21_FN_6_O_4_S_2_: C, 49.99; H, 4.20; N, 16.66. Found: C, 49.95; H, 4.17; N, 16.62. HRMS (*m*/*z*): Cal for C_21_H_21_FN_6_O_4_S_2_: [M + H]^+^*m*/*z*: 505.1050, found: 521.1053.

#### 3-(4-((1-((4-Nitrophenyl)sulfonyl)-1*H*-1,2,3-triazol-4-yl)methyl)piperazin-1-yl)-2*H*-benzo[*b*][1,4]thiazine 1,1-dioxide (8f)

Yellow solid; M.p: 169–171 °C; ^1^H-NMR (500 MHz, CDCl_3_) *δ* 8.48 (d, *J* = 8.0 Hz, 2H), 8.25 (d, *J* = 8.0 Hz, 2H), 8.13 (s, 1H, tri-H), 7.92 (d, *J* = 8.0 Hz, 1H), 7.73 (d, *J* = 8.0 Hz, 1H), 7.64–7.60 (m, 1H), 7.44–7.39 (m, 1H), 5.28 (s, 2H, N–CH_2_), 4.38 (s, 2H, SO_2_–CH_2_), 3.79 (t, *J* = 4.0 Hz, 4H, 2N–CH_2_), 3.56 (t, *J* = 4.0 Hz, 4H, 2N–CH_2_). ^13^C-NMR (126 MHz, CDCl_3_): *δ* 153.22, 147.29, 146.19, 142.33, 135.33, 133.46, 132.17, 130.28, 129.72, 127.67 (2C), 124.34, 122.63 (2C), 122.10, 59.77, 49.74, 46.68 (2C), 44.13 (2C). ESI-MS: 532.09 [M + H]. Anal. calcd for C_21_H_21_N_7_O_6_S_2_: C, 47.45; H, 3.98; N, 18.44. Found: C, 47.41; H, 3.95; N, 18.46.

#### 4-((4-((4-(1,1-Dioxido-2*H*-benzo[*b*][1,4]thiazin-3-yl)piperazin-1-yl)methyl)-1*H*-1,2,3-triazol-1-yl)sulfonyl)benzonitrile (8g)

White solid; M.p: 159–161 °C; ^1^H-NMR (500 MHz, CDCl_3_) *δ* 8.03 (s, 1H, tri-H), 7.91 (d, *J* = 8.0 Hz, 1H), 7.81 (d, *J* = 8.0 Hz, 2H), 7.72 (d, *J* = 8.0 Hz, 1H), 7.63–7.58 (m, 1H), 7.50 (d, *J* = 8.0 Hz, 2H), 7.41–7.36 (m, 1H), 5.26 (s, 2H, N–CH_2_), 4.35 (s, 2H, SO_2_–CH_2_), 3.77 (t, *J* = 4.0 Hz, 4H, 2N–CH_2_), 3.56 (t, *J* = 4.0 Hz, 4H, 2N–CH_2_). ^13^C-NMR (126 MHz, CDCl_3_): *δ* 153.49, 146.35, 145.13, 135.40, 133.43(2C), 132.18, 131.05, 129.93, 129.22, 127.33(2C), 124.42, 122.78, 120.58, 119.18, 59.74, 49.31, 46.78(2C), 44.33(2C). ESI-MS: 512.10 [M + H]. Anal. calcd for C_22_H_21_N_7_O_4_S_2_: C, 51.65; H, 4.14; N, 19.17. Found: C, 51.69; H, 4.11; N, 19.14.

Minimum inhibitory concentration (MIC) of the synthesized compounds was determined by broth dilution method according to the Clinical and Laboratory Standards Institute (CLSI) guidelines. Separately, each stock solution of the compounds synthesized was prepared in 1.5 mL of dimethylsulphoxide (DMSO) to achieve final concentration of 64 μg mL^−1^. The serial dilutions from the stock was made in 96-well microplates using Mueller–Hinton broth (Becton Dickinson, Sparks, MD, USA) to obtain the different concentrations of the test compounds ranging from 32 μg mL^−1^ to 0.25 μg mL^−1^. The Staphylococcus *aureus* mutant strain suspension (Approx. 5 × 10^5^ colony-forming units mL^−1^) was prepared from 24 h fresh culture. Of about 100 μl of this culture was transferred each well of the 96 well plate that contain different concentrations of synthesized compounds and incubated at 37 °C for 24 h. Approximately 40 μl 0.4 μg mL^−1^ solution of resazurin (microbial growth indicator) was added to each well of the plate maintained at 37 °C for 30 min. The MIC values visually evaluated. The lowest concentration of synthesized compounds exhibit no visible growth was noted as the MIC of that compound. The experiment was conducted in triplicate.

### Measurement of hemolytic activity

The hemolytic activity of the 6e, 6f, 6g, 6i, 8d, 8e was assayed against human red blood cells (hRBCs) by measuring the amount of hemoglobin released after treatment. The hRBCs, freshly collected from a healthy volunteer in polycarbonate tubes containing heparin, were washed three times in sterile phosphate-buffered saline (PBS) and centrifuged at 2000×*g* for 5 min or until the supernatant became clear. The hRBCs were diluted to a final concentration of 2% (vol/vol), then 50 μl of the hRBCs suspension was incubated with 50 μl of different concentrations (0.75 to 100 μg mL^−1^) of the tested compounds dissolved in PBS. After 1 h of incubation at 37 °C, intact hRBCs were pelleted by centrifugation at 2000×*g* for 10 min. The supernatant was transferred to a new 96-well plate and the release of hemoglobin was monitored by measurement of absorbance at 405 nm using a Multiskan FC microplate reader. The hRBCs in PBS only (ODBlank) and in 0.1% Triton X-100 (ODTriton X-100) were employed as negative (0% hemolysis) and positive (100% hemolysis) controls, respectively. The percentage of hemolysis was calculated according to the following equation:% Hemolysis = (OD_Sample_ − A_Blank_)/(OD_TritonX-100_ − OD_Blank_) × 100

### Molecular docking studies

Docking simulation were performed by using AutoDock VINA integrated in the PyRx 0.8 virtual screening tool to identify compounds with high binding affinity.^36,37^*In silico* docking studies to evaluate the molecular interaction profile and binding affinity variations of 6e, 6f, 6g, 6i, 8d, 8e compounds were done with the Human, Mouse and Bovine Toll-like receptor (TLR4) proteins with PDB ID's 3FXI, 3VQ1, 3RG1 respectively. Protein structures were processed to ensure an optimized structure for docking studies and it was executed with UCSF Chimera Dock Prep module and that includes the following steps: elimination of water molecules and other ligands, addition of missing atoms and residues, energy minimization and assigning charges and polar hydrogens and then converted to the pdbqt format. The 2D structure of the ligands was drawn with ChemDraw software and the structures were optimized through energy minimization with MMFF94 force field parameters and conjugate gradient algorithm using Open Babel module of PyRx and eventually converted the ligands to the AutoDock compatible pdbqt format to carry out docking exploration. Post docking analysis and visualization of binding poses and molecular interactions were done with BIOVIA Discovery Studio 2021 and Chimera X tools.

Binding energies and molecular interaction profile of the compounds were compared with the Human, Mouse and Bovine TLR4 proteins. Autodock Vina grid box was created around the whole protein in 3FXI, 3VQ1 and 3RG1 proteins with the following details of the vina search space. Binding affinity outcomes of compounds from the docking assessments are as follows.

**Table:** 

	3FXI	3VQ1	3RG1
**Centre (Å)**			
*x*	3.2577	7.6491	−1.8714
*y*	4.7519	18.7324	−8.9592
*z*	21.6834	28.9341	−0.9964

**Dimensions (Å)**			
*x*	74.3523	90.9402	81.4867
*y*	87.0448	106.1912	92.6772
*z*	97.1752	88.0768	95.9081

## Conflicts of interest

The authors declare no conflict of interest.

## Supplementary Material

RA-014-D3RA07509E-s001
