# Supplementary material for: Synthesis of 1,2,3-triazole-piperazin-benzo[b][1,4]thiazine 1,1-dioxides: antibacterial, hemolytic and in silico TLR4 protein inhibitory activities
Source: RSC Adv. 2024 Mar 18;14(13):8921–31. doi: 10.1039/d3ra07509e (PMC10945376; doi:10.1039/d3ra07509e)
Supplement: RA-014-D3RA07509E-s001 [file RA-014-D3RA07509E-s001.pdf]

Electronic Supplementary Material (ESI) for RSC Advances.
This journal is © The Royal Society of Chemistry 2024

Supporting information

Synthesis of 1,2,3-triazole-piperazin-benzo[*b*][1,4]thiazine 1,1-dioxides: Antibacterial, Hemolytic and *In silico* TLR4 protein inhibitory activities

Nagavelli Ramu,[a] Thupurani Murali Krishna,[b] Ravikumar Kapavarapu,[c] Sirassu Narsimha*[a]

[a]*Department of Chemistry, Chaitanya Deemed to be University, Hyderabad, Telangana, India*

[b]*Department of Biotechnology, Chaitanya Deemed to be University, Hyderabad, Telangana, India*

[c]*Department of Pharmaceutical Chemistry and Phytochemistry, Nirmala College of Pharmacy, Atmakur, Mangalgiri, Andhra Pradesh, India*

**E-mail: narsimha.s88@gmail.com & narsimha.s88@chaitanya.edu.in*

Content

**Docking figures**
**Copies of ^{1}H-NMR, ^{13}C-NMR, Mass**

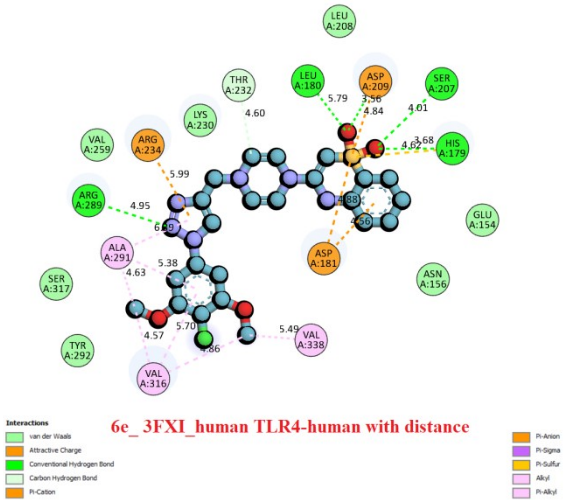

6e_ 3FXI_human TLR4-human with distance

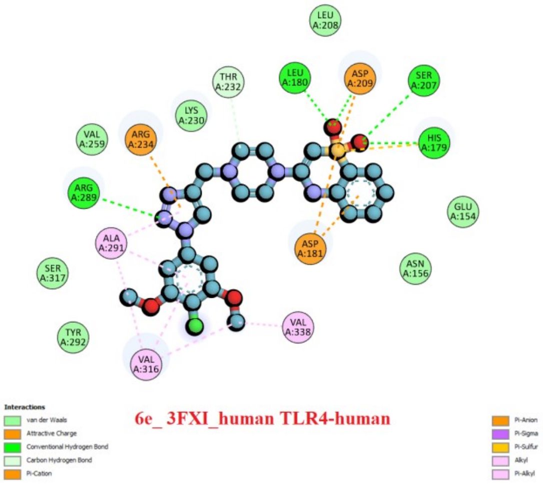

6e_ 3FXI_human TLR4-human

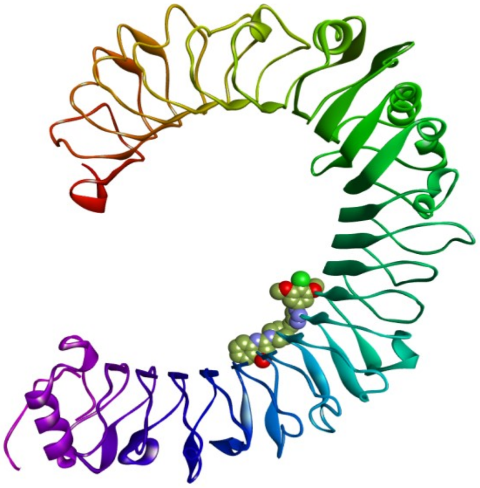

6e_ 3VQ1_mouse TLR4 PL complex in the binding site

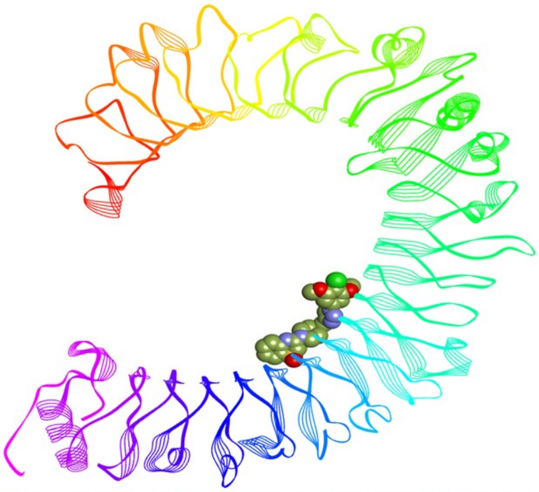

6e_ 3VQ1_mouse TLR4 PL complex in the binding site_1

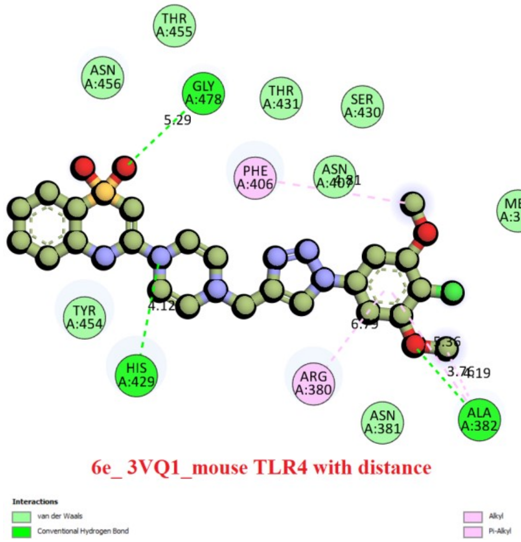

6e_ 3VQ1_mouse TLR4 with distance

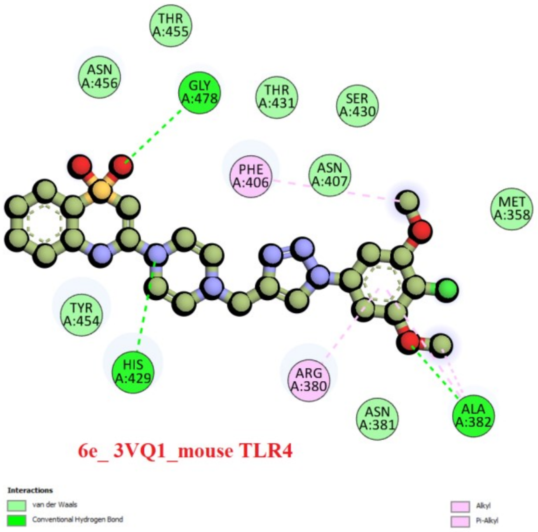

6e_ 3VQ1_mouse TLR4

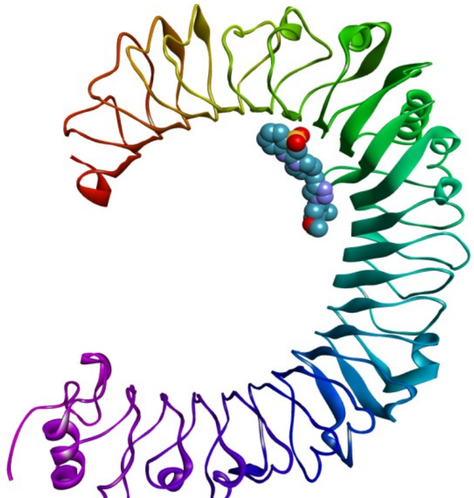

6e_3FXI_human TLR4-human binding site

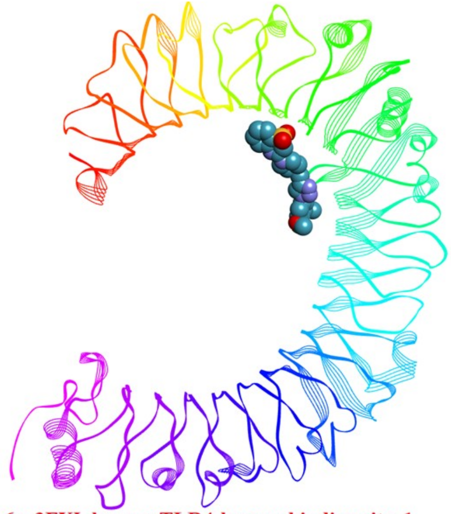

6e_3FXI_human TLR4-human binding site_1

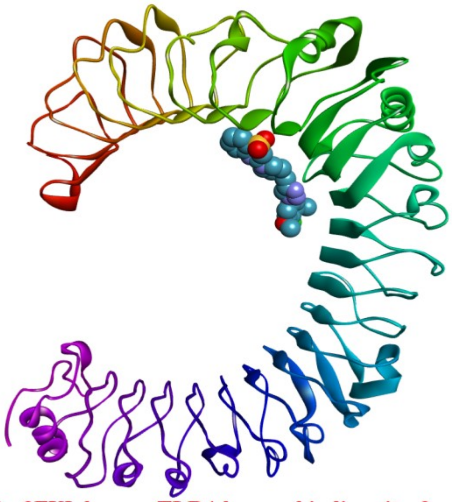

6e_3FXI_human TLR4-human binding site_2

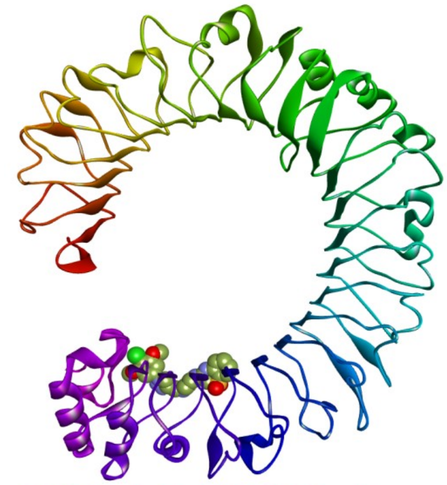

6e_3RG1_ bovine TLR4 binding site

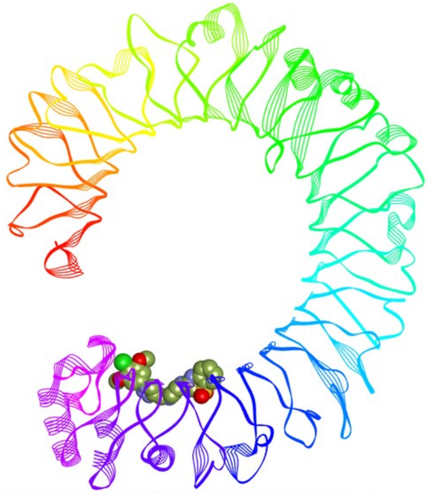

6e_3RG1_ bovine TLR4 binding site_1

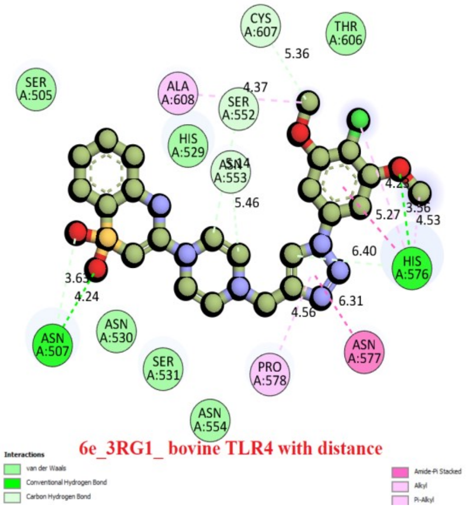

6e_3RG1_ bovine TLR4 with distance

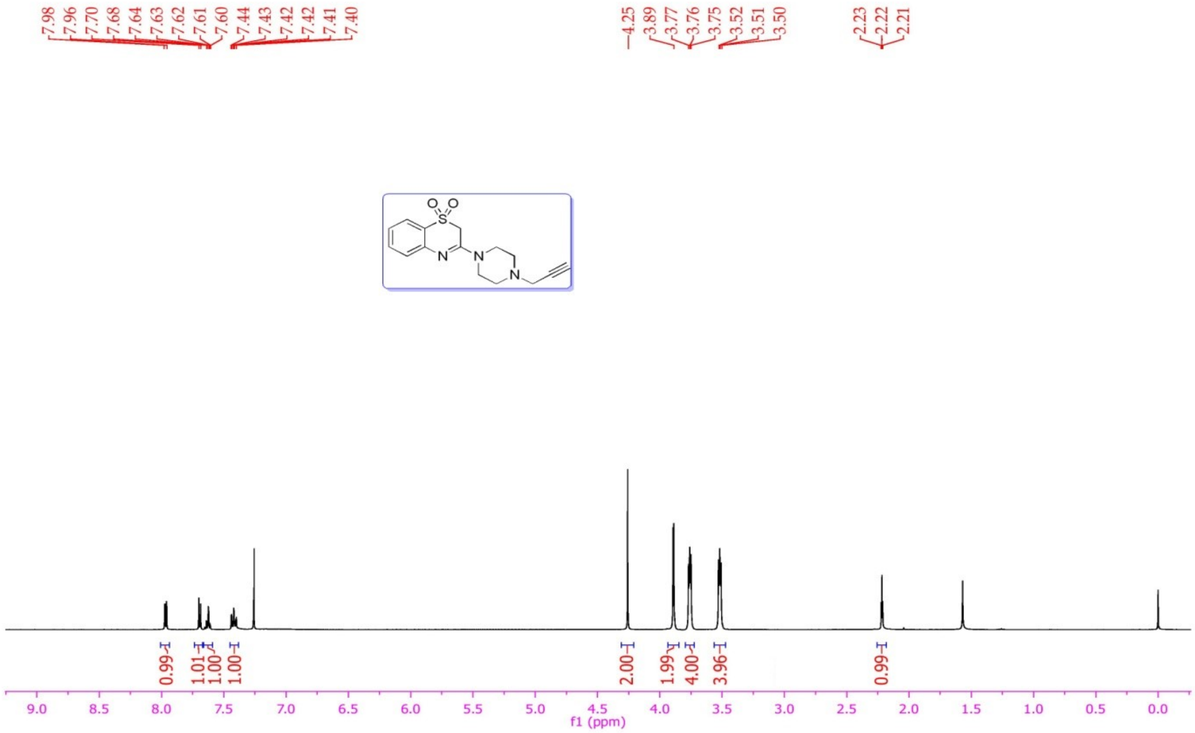
7.98 7.96 7.70 7.68 7.64 7.63 7.62 7.61 7.60 7.44 7.43 7.42 7.42 7.41 7.40
4.25 3.89 3.77 3.76 3.75 3.52 3.51 3.50
2.23 2.22 2.21
0.99 1.01 1.00 1.00 2.00 1.99 4.00 3.96 0.99
9.0 8.5 8.0 7.5 7.0 6.5 6.0 5.5 5.0 4.5 4.0 3.5 3.0 2.5 2.0 1.5 1.0 0.5 0.0
f1 (ppm)

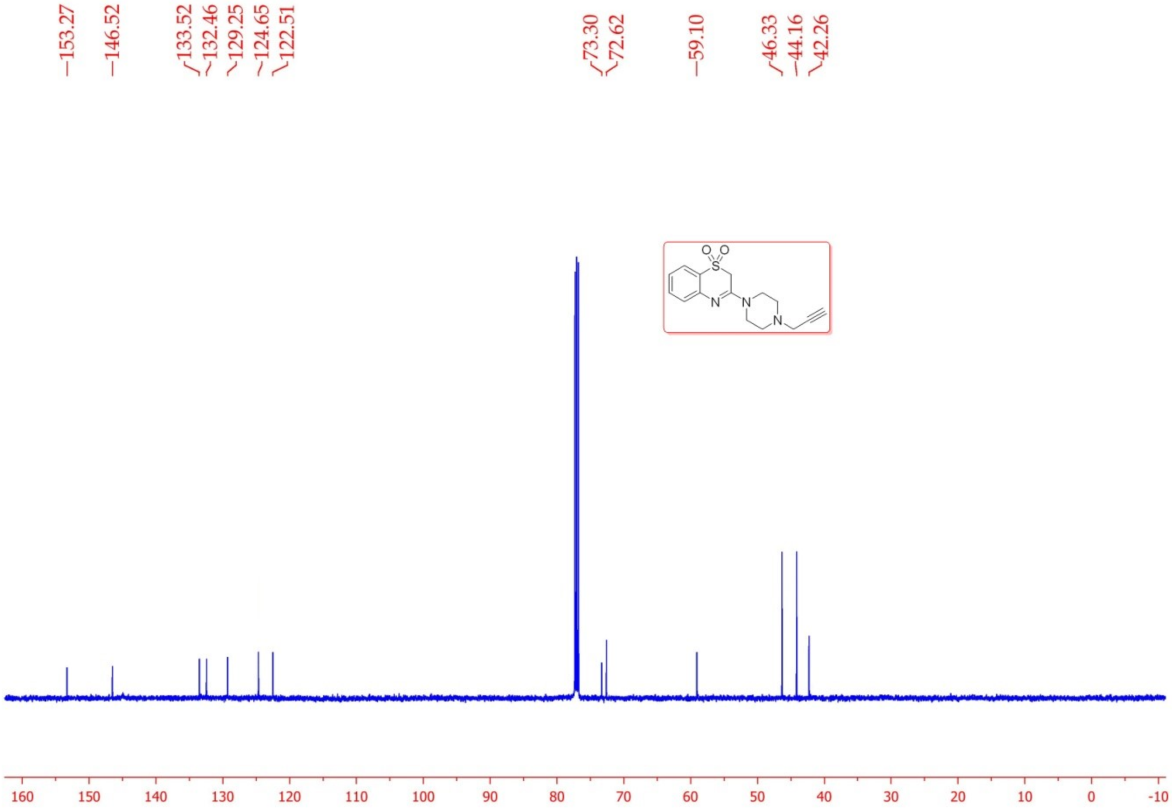
153.27
146.52
133.52 132.46 129.25 124.65 122.51
73.30 72.62
59.10
46.33 44.16 42.26
160 150 140 130 120 110 100 90 80 70 60 50 40 30 20 10 0 -10

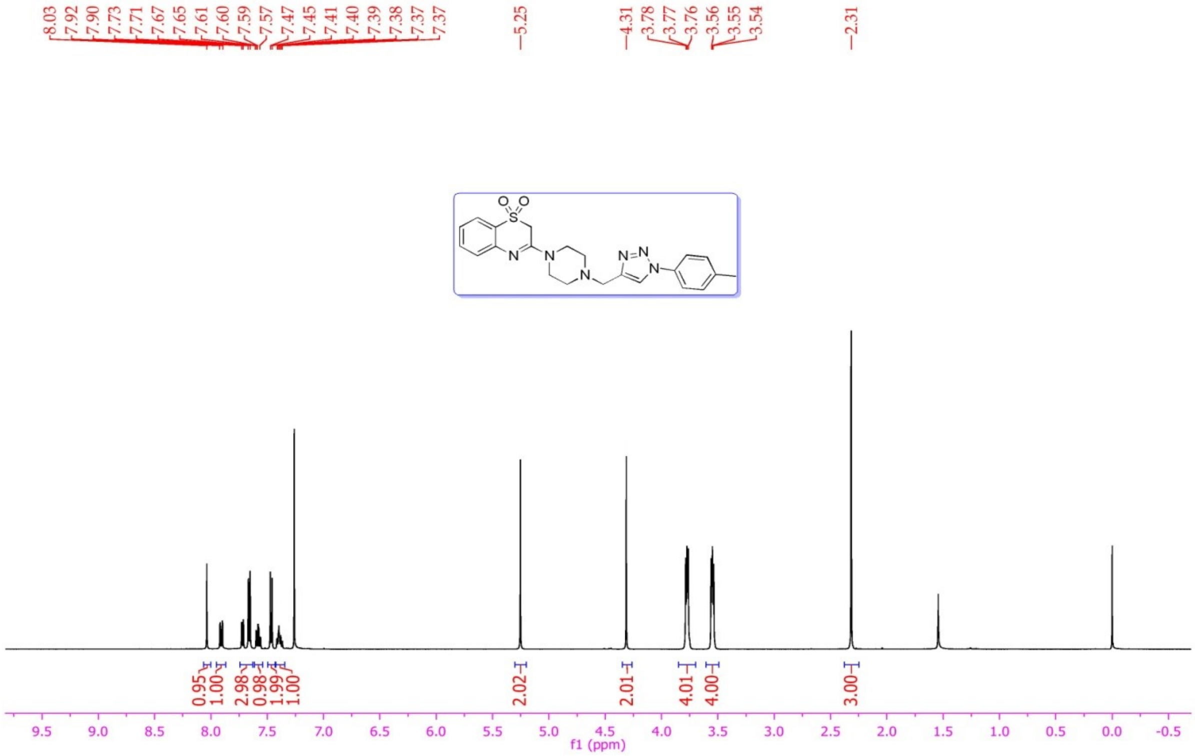
8.03
7.92
7.90
7.73
7.71
7.67
7.65
7.61
7.60
7.59
7.57
7.47
7.45
7.41
7.40
7.39
7.38
7.37
7.37
5.25
4.31
3.78
3.77
3.76
3.56
3.55
3.54
2.31
0.95
1.00
2.98
0.98
1.99
1.00
2.02
2.01
4.01
4.00
3.00
f1 (ppm)

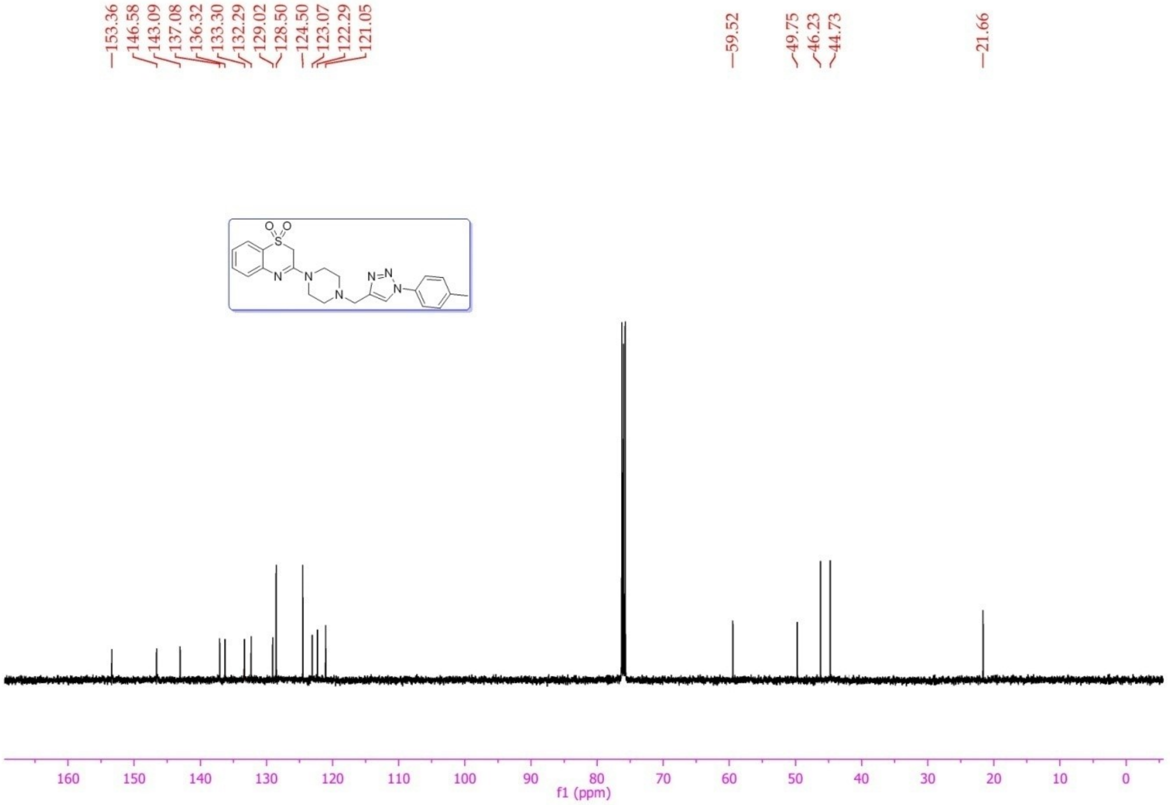
153.36
146.58
143.09
137.08
136.32
133.30
132.29
129.02
128.50
124.50
123.07
122.29
121.05
59.52
49.75
46.23
44.73
21.66
f1 (ppm)

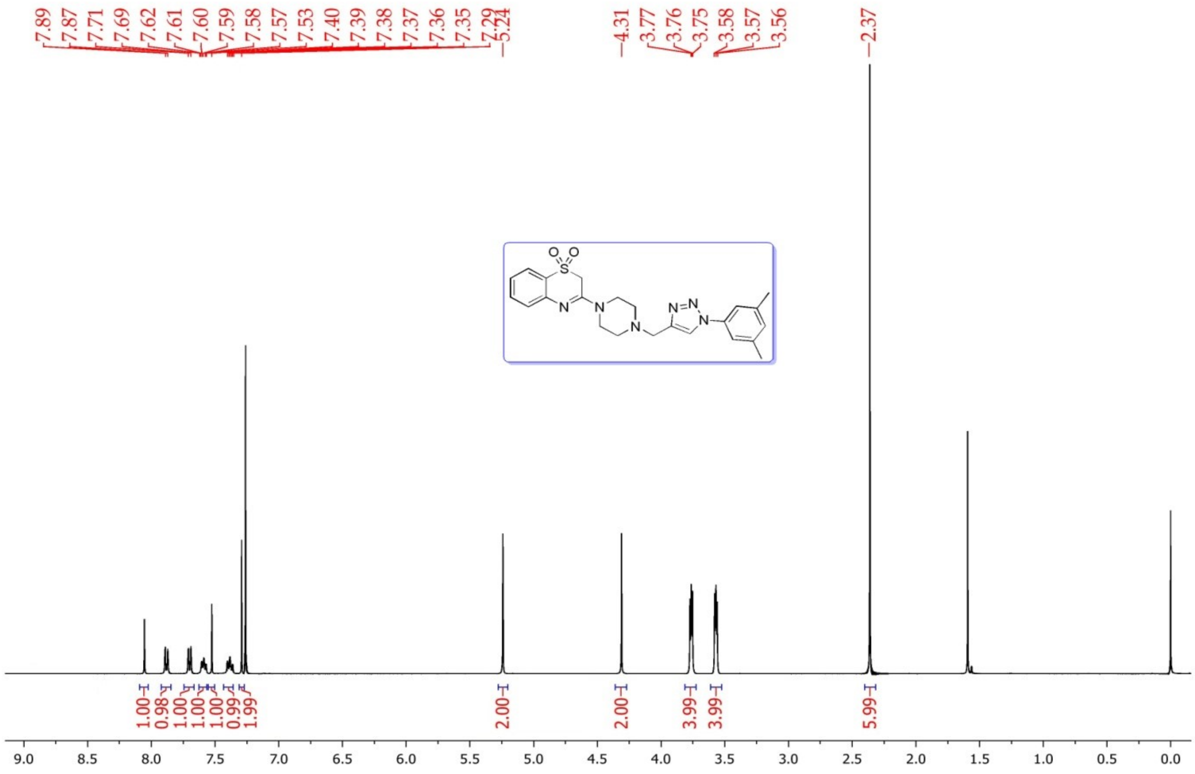

7.89 7.87 7.71 7.69 7.62 7.61 7.60 7.59 7.58 7.57 7.53 7.40 7.39 7.38 7.37 7.36 7.35 7.29 7.24
4.31 3.77 3.76 3.75 3.58 3.57 3.56
2.37
1.00 0.98 1.00 1.00 1.00 0.99 1.99
2.00
2.00
3.99
3.99
5.99
9.0 8.5 8.0 7.5 7.0 6.5 6.0 5.5 5.0 4.5 4.0 3.5 3.0 2.5 2.0 1.5 1.0 0.5 0.0

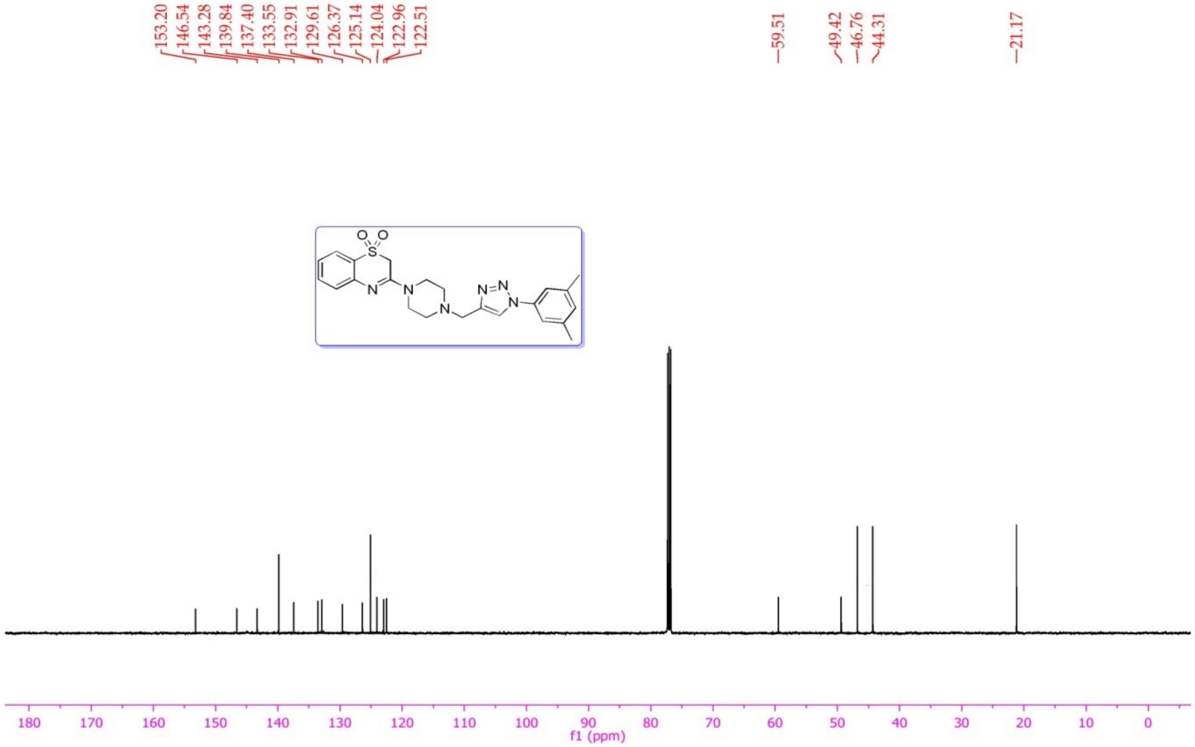

153.20 146.54 143.28 139.84 137.40 133.55 132.91 129.61 126.37 125.14 124.04 122.96 122.51
59.51
49.42 46.76 44.31
21.17
180 170 160 150 140 130 120 110 100 90 80 70 60 50 40 30 20 10 0
f1 (ppm)

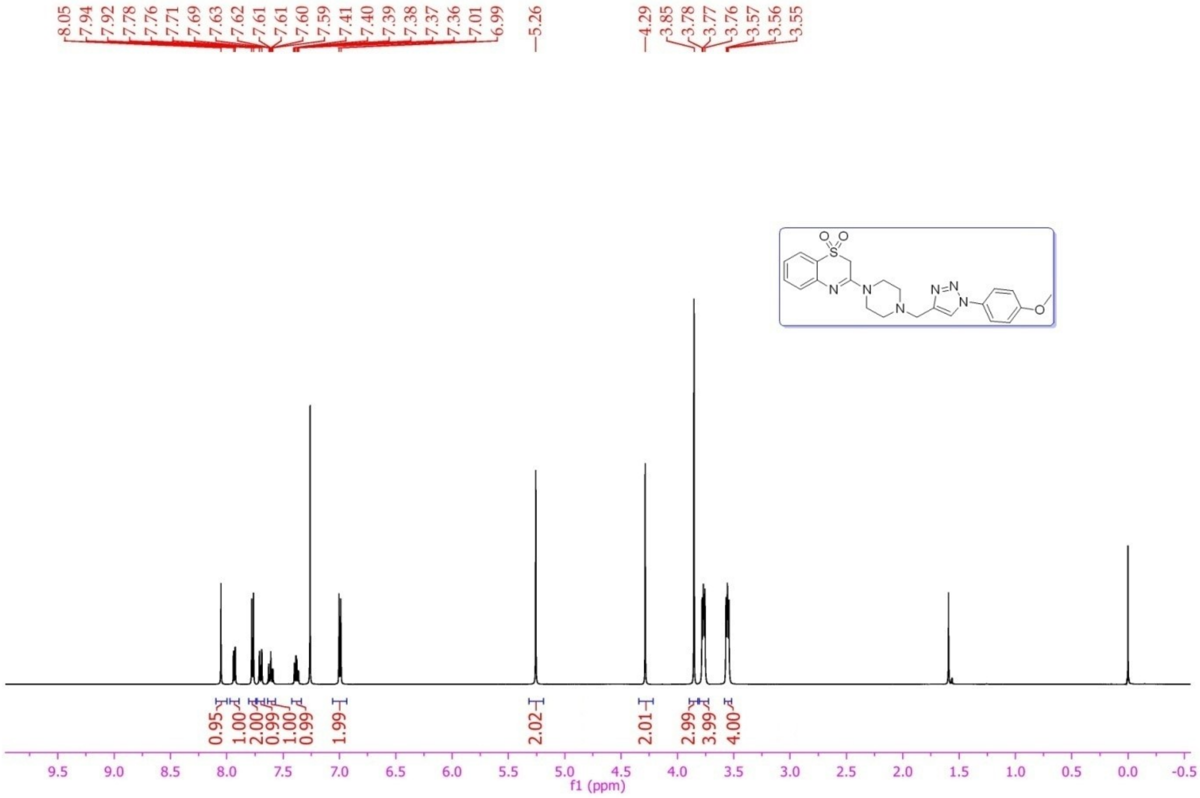
8.05
7.94
7.92
7.78
7.76
7.71
7.69
7.63
7.62
7.61
7.61
7.60
7.59
7.41
7.40
7.39
7.38
7.37
7.36
7.01
6.99
5.26
4.29
3.85
3.78
3.77
3.76
3.57
3.56
3.55
0.95
1.00
2.00
0.99
1.00
0.99
1.99
2.02
2.01
2.99
3.99
4.00
9.5 9.0 8.5 8.0 7.5 7.0 6.5 6.0 5.5 5.0 4.5 4.0 3.5 3.0 2.5 2.0 1.5 1.0 0.5 0.0 -0.5
f1 (ppm)

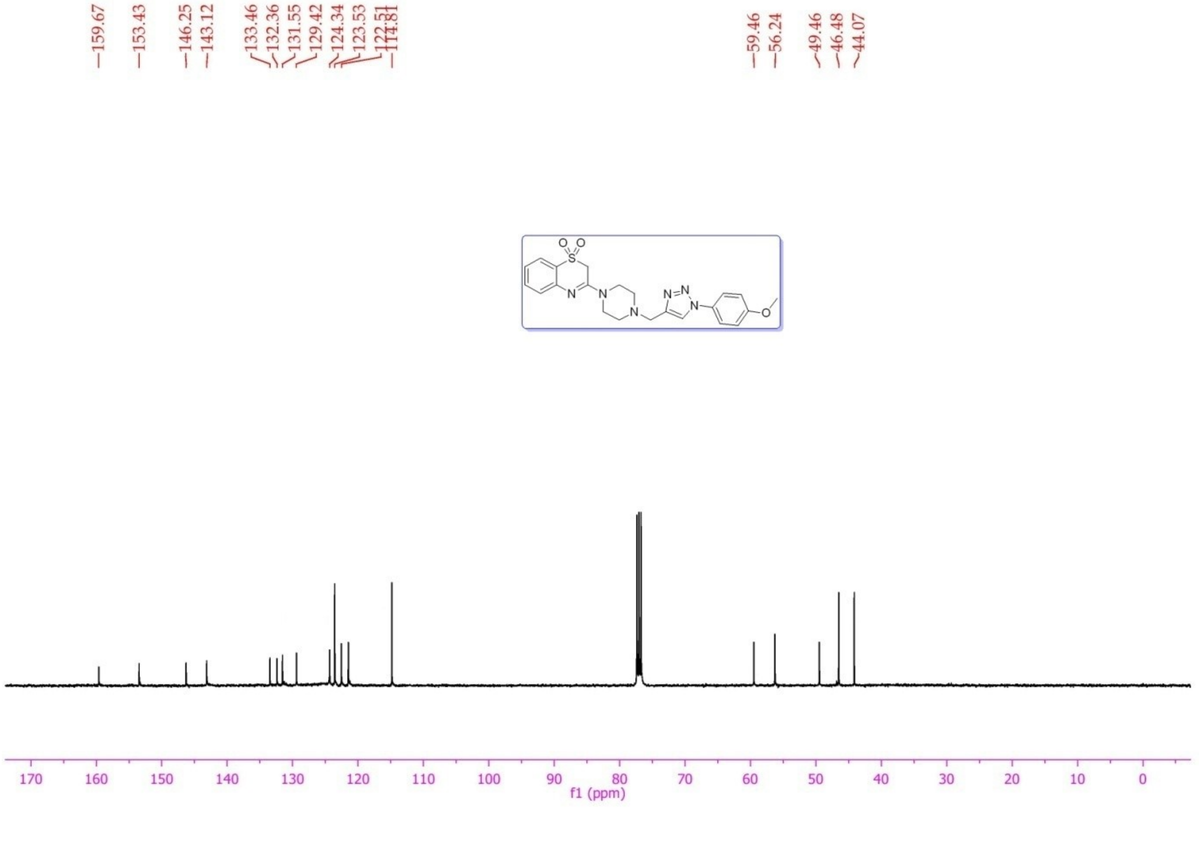
159.67
153.43
146.25
143.12
133.46
132.36
131.55
129.42
124.34
123.53
122.51
114.81
59.46
56.24
49.46
46.48
44.07
170 160 150 140 130 120 110 100 90 80 70 60 50 40 30 20 10 0
f1 (ppm)

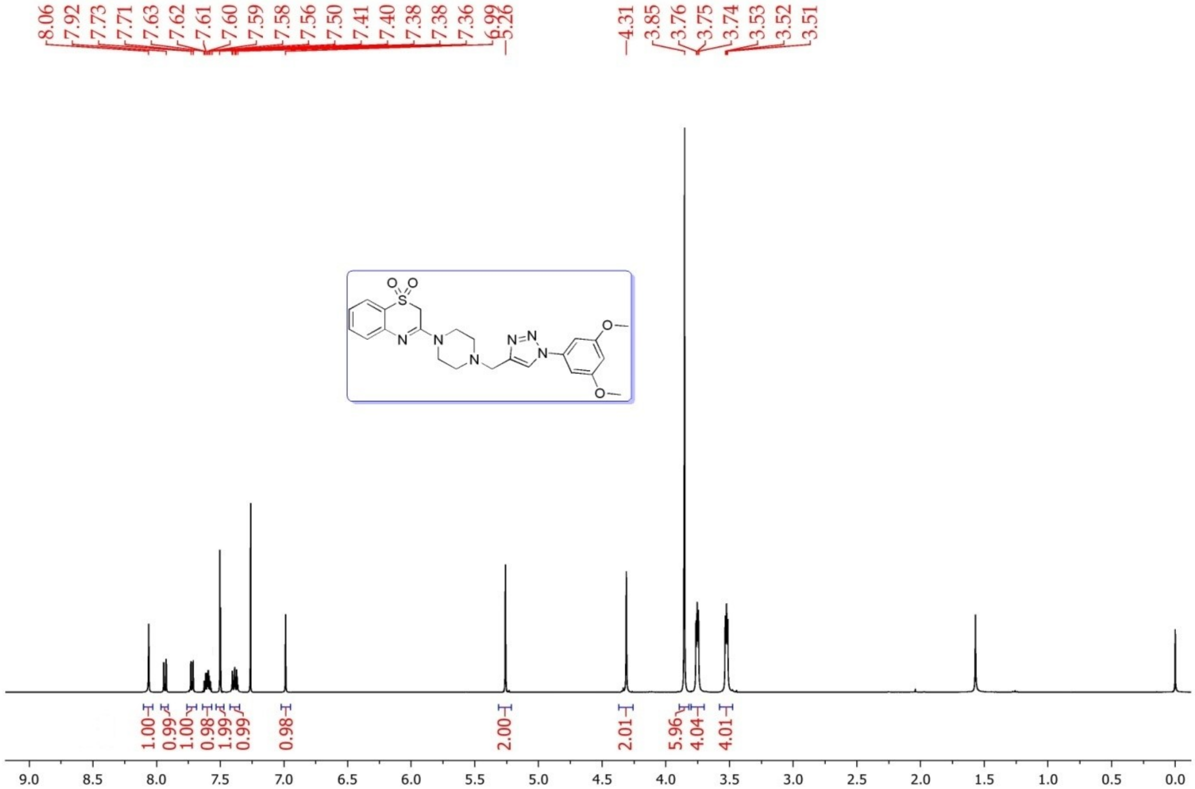
8.06 7.92 7.73 7.71 7.63 7.62 7.61 7.60 7.59 7.58 7.56 7.50 7.41 7.40 7.38 7.38 7.36 6.99 5.26
4.31 3.85 3.76 3.75 3.74 3.53 3.52 3.51
1.00 0.99 1.00 0.98 1.99 0.99 0.98 2.00 2.01 5.96 4.04 4.01
9.0 8.5 8.0 7.5 7.0 6.5 6.0 5.5 5.0 4.5 4.0 3.5 3.0 2.5 2.0 1.5 1.0 0.5 0.0

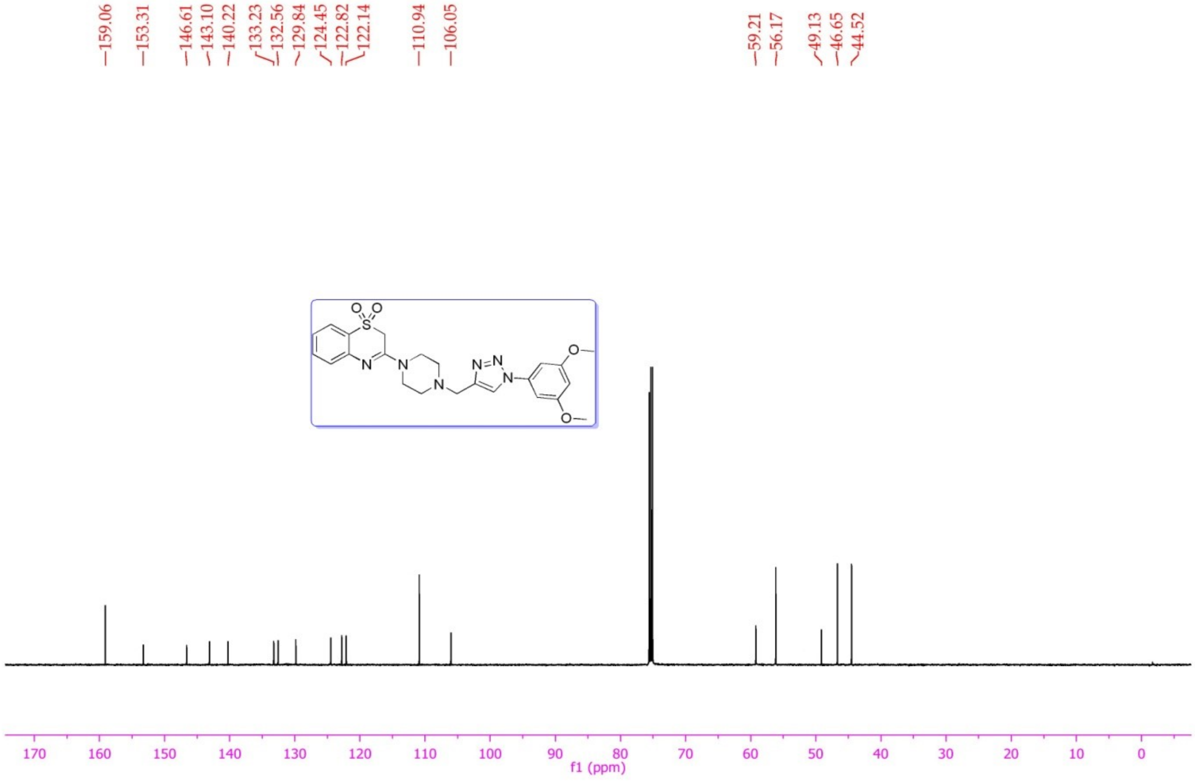
159.06 153.31 146.61 143.10 140.22 133.23 132.56 129.84 124.45 122.82 122.14 110.94 106.05
59.21 56.17 49.13 46.65 44.52
170 160 150 140 130 120 110 100 90 80 70 60 50 40 30 20 10 0
f1 (ppm)

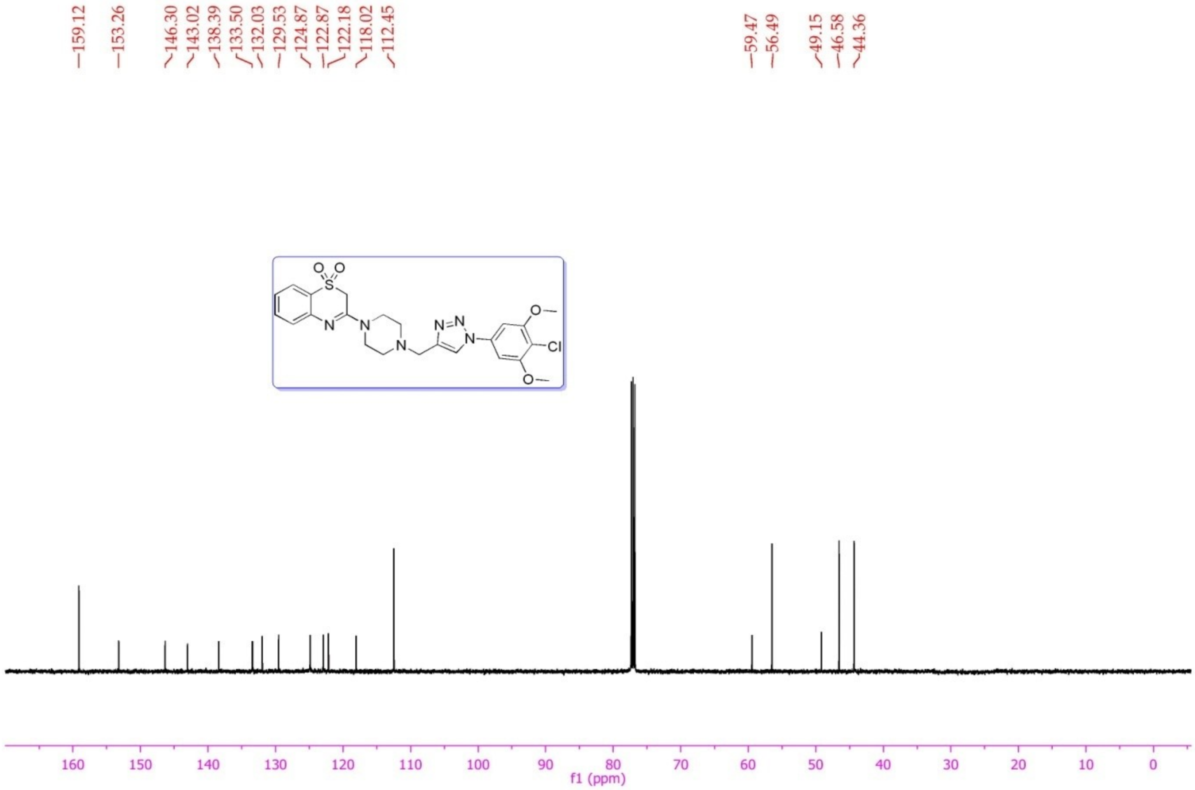
159.12
153.26
146.30
143.02
138.39
133.50
132.03
129.53
124.87
122.87
122.18
118.02
112.45
59.47
56.49
49.15
46.58
44.36
160 150 140 130 120 110 100 90 80 70 60 50 40 30 20 10 0
f1 (ppm)

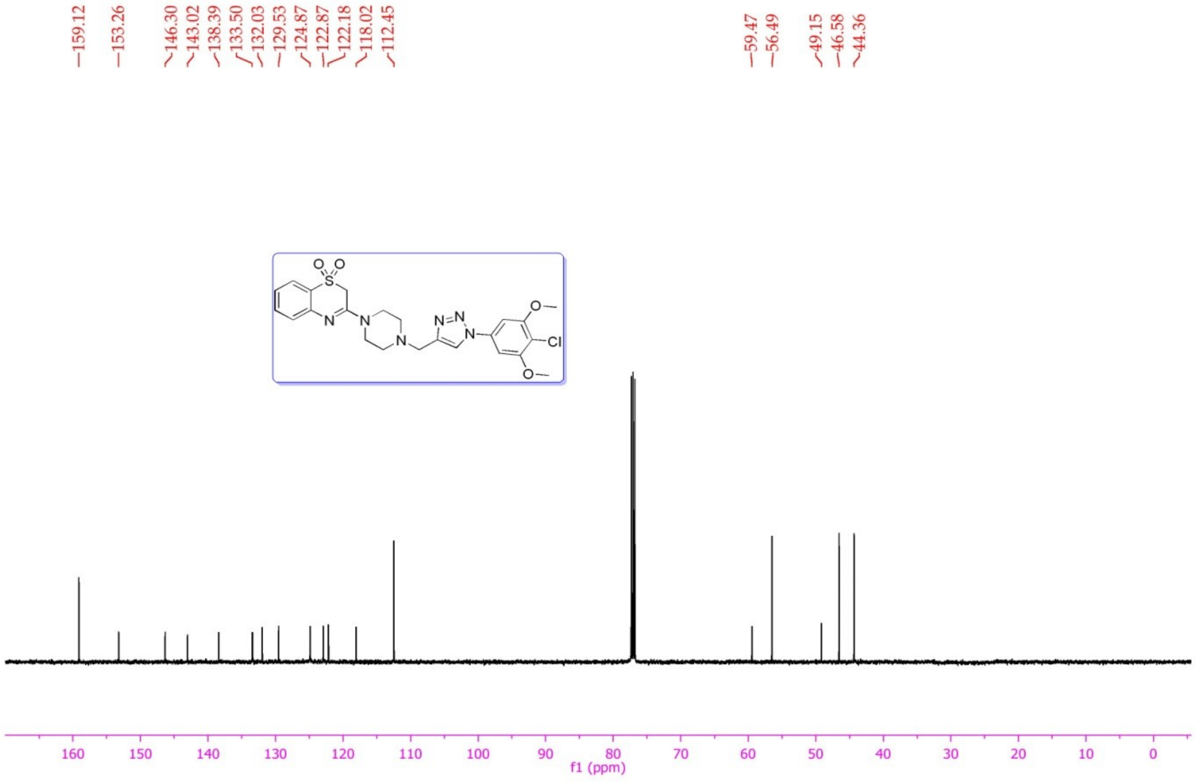
159.12
153.26
146.30
143.02
138.39
133.50
132.03
129.53
124.87
122.87
122.18
118.02
112.45
59.47
56.49
49.15
46.58
44.36
160 150 140 130 120 110 100 90 80 70 60 50 40 30 20 10 0
f1 (ppm)

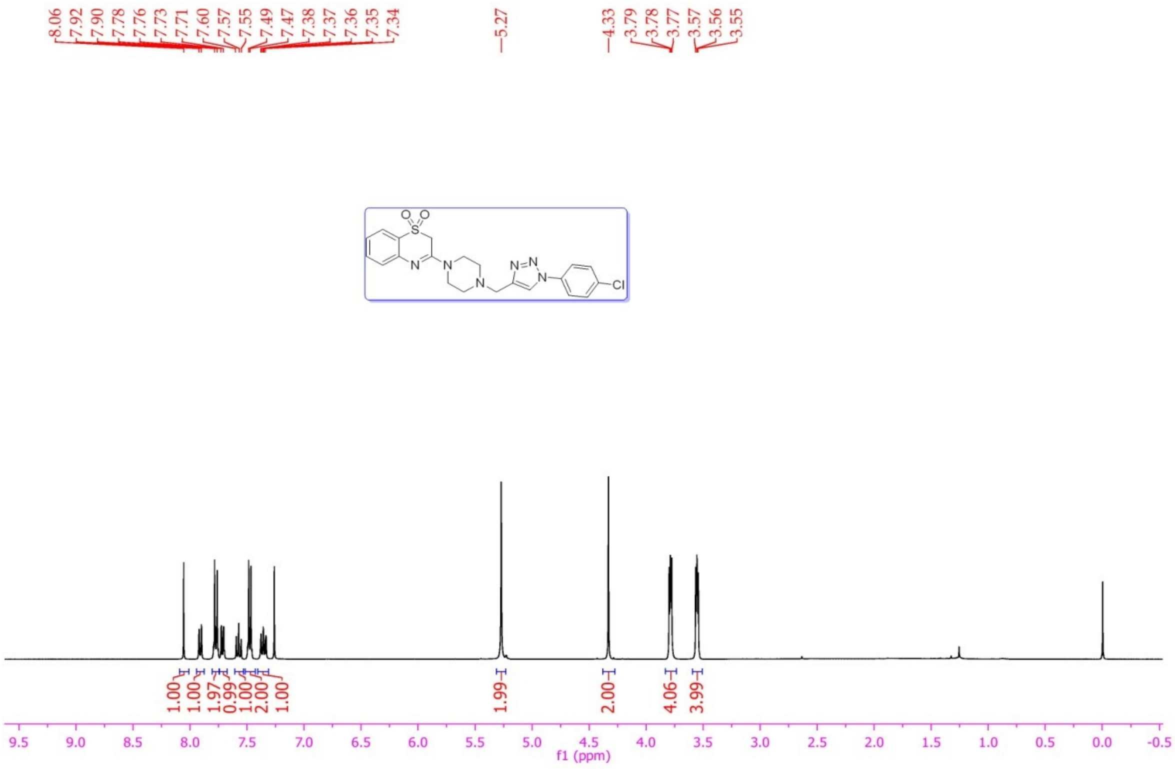
8.06
7.92
7.90
7.78
7.76
7.73
7.71
7.60
7.57
7.55
7.49
7.47
7.38
7.37
7.36
7.35
7.34
5.27
4.33
3.79
3.78
3.77
3.57
3.56
3.55
O
O
S
N
N
N
N
N
N
Cl
1.00
1.00
1.97
0.99
1.00
2.00
1.00
1.99
2.00
4.06
3.99
9.5
9.0
8.5
8.0
7.5
7.0
6.5
6.0
5.5
5.0
4.5
4.0
3.5
3.0
2.5
2.0
1.5
1.0
0.5
0.0
-0.5
f1 (ppm)

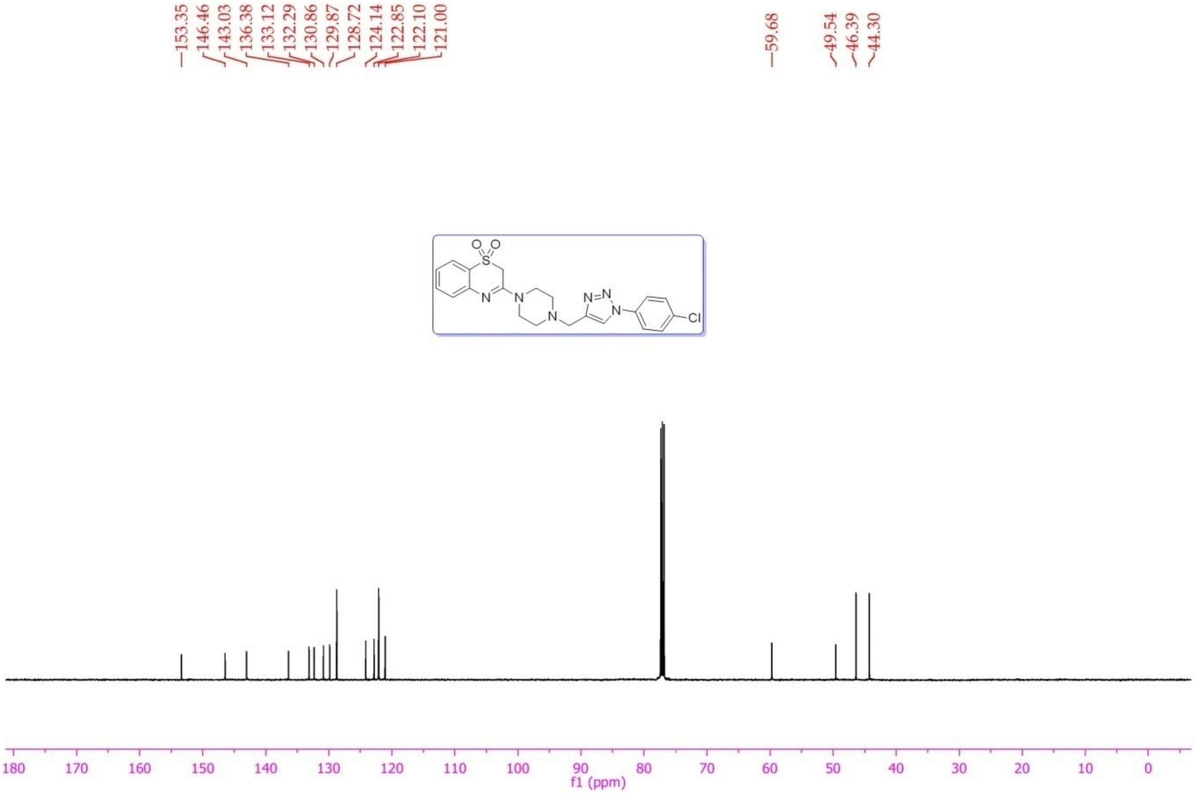
153.35
146.46
143.03
136.38
133.12
132.29
130.86
129.87
128.72
124.14
122.85
122.10
121.00
59.68
49.54
46.39
44.30
O
O
S
N
N
N
N
N
N
Cl
180
170
160
150
140
130
120
110
100
90
80
70
60
50
40
30
20
10
0
f1 (ppm)

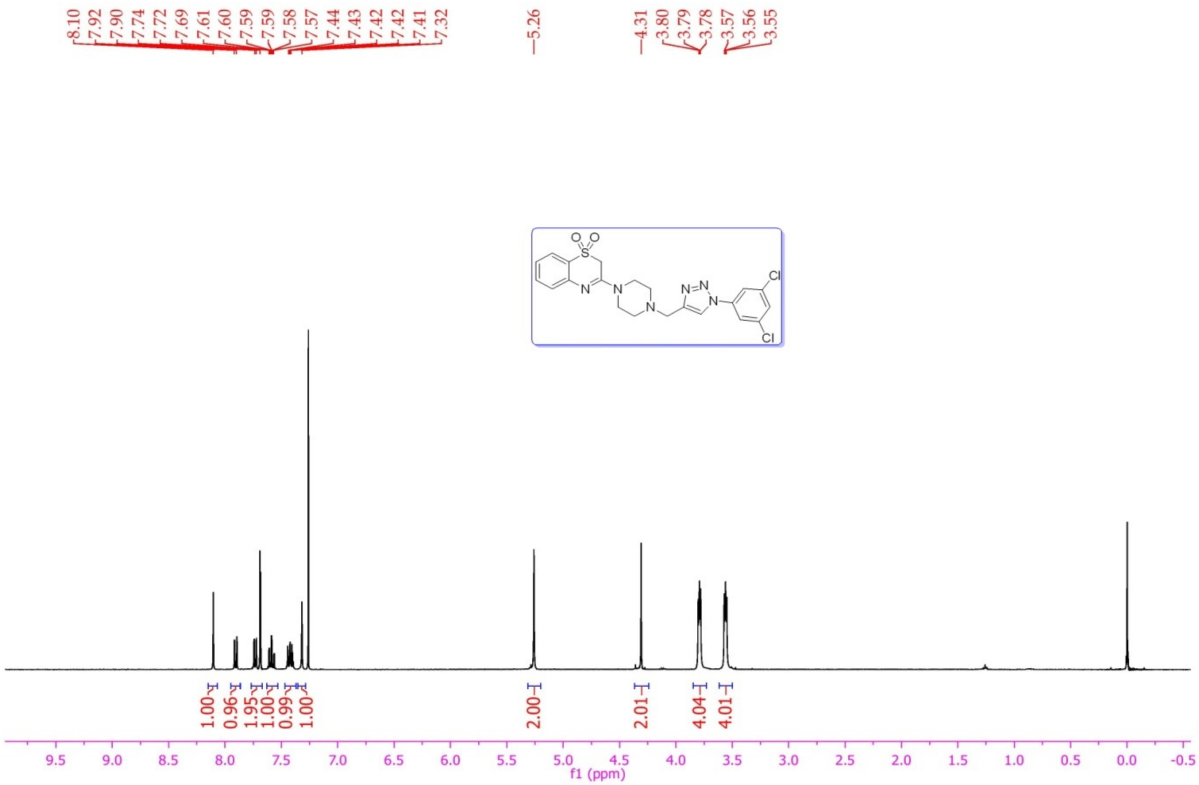
8.10 7.92 7.90 7.74 7.72 7.69 7.61 7.60 7.59 7.59 7.58 7.57 7.44 7.43 7.42 7.42 7.41 7.32
5.26
4.31 3.80 3.79 3.78 3.57 3.56 3.55
Cl
Cl
1.00 0.96 1.95 1.00 0.99 1.00
2.00
2.01
4.04 4.01
9.5 9.0 8.5 8.0 7.5 7.0 6.5 6.0 5.5 5.0 4.5 4.0 3.5 3.0 2.5 2.0 1.5 1.0 0.5 0.0 -0.5
f1 (ppm)

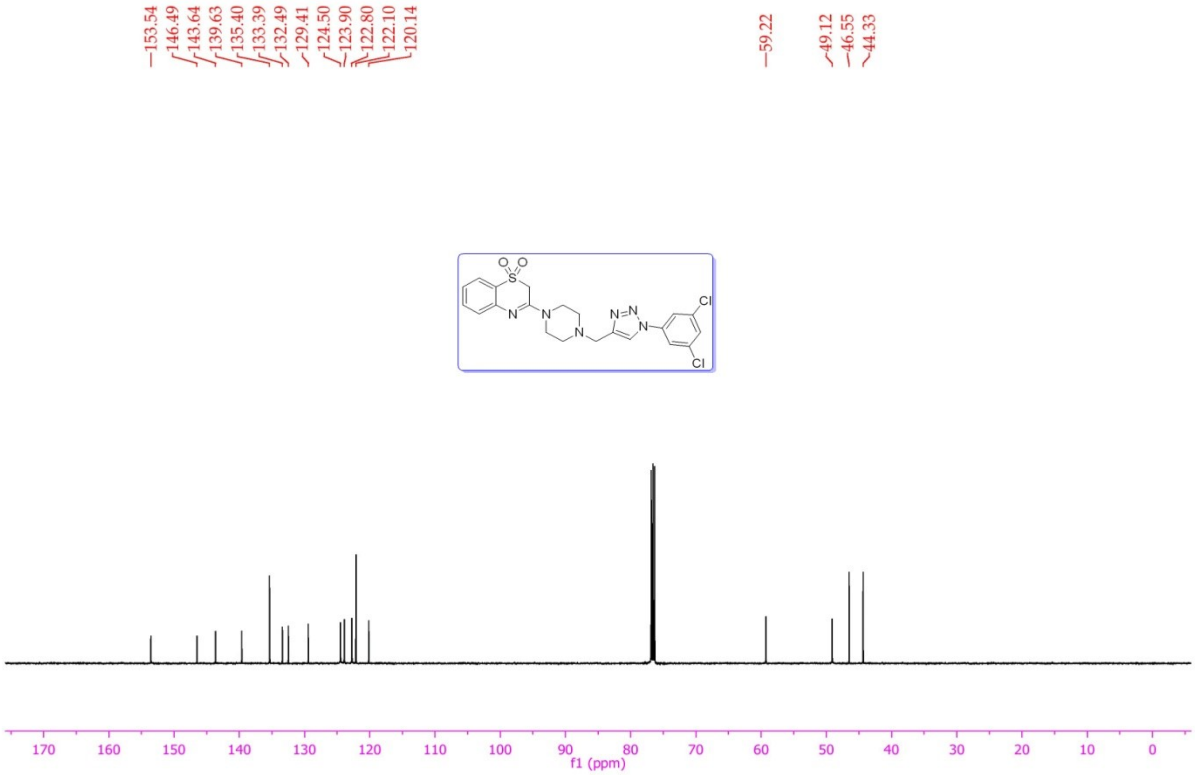
153.54 146.49 143.64 139.63 135.40 133.39 132.49 129.41 124.50 123.90 122.80 122.10 120.14
59.22
49.12 46.55 44.33
Cl
Cl
170 160 150 140 130 120 110 100 90 80 70 60 50 40 30 20 10 0
f1 (ppm)

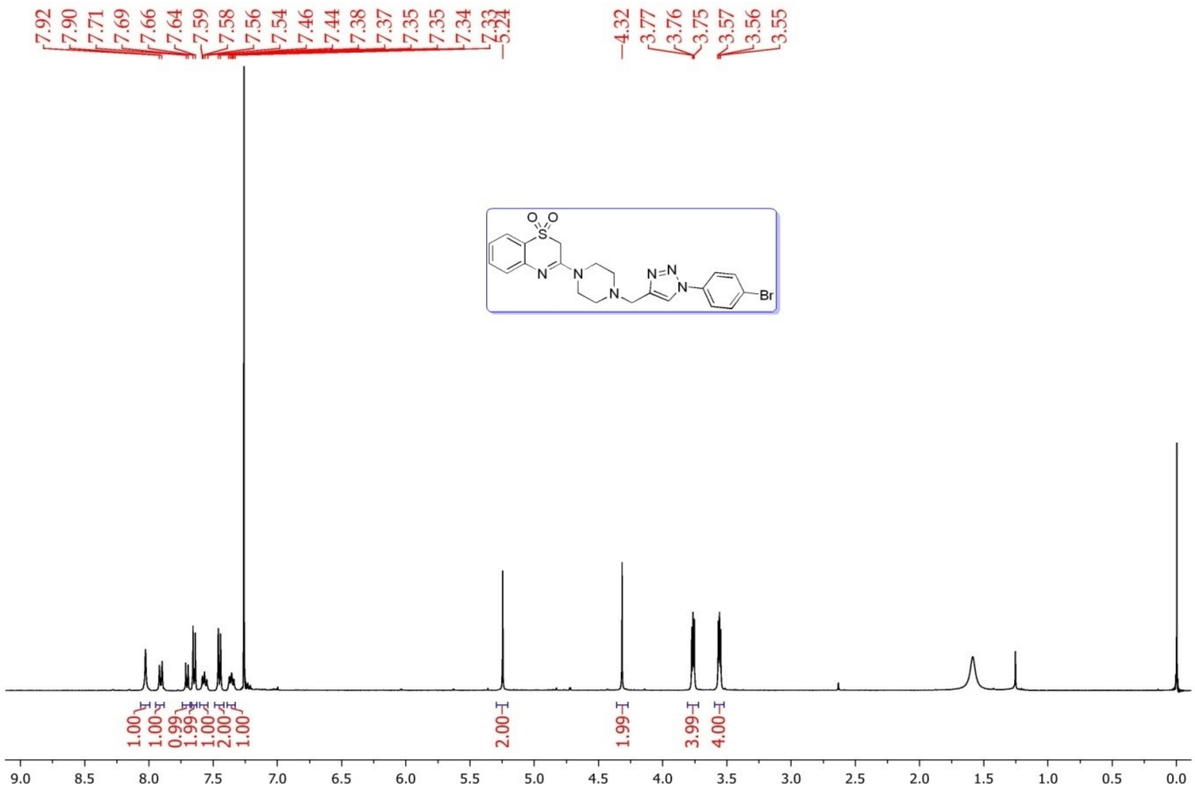
7.92
7.90
7.71
7.69
7.66
7.64
7.59
7.58
7.56
7.54
7.46
7.44
7.38
7.37
7.35
7.35
7.34
7.33
5.24
4.32
3.77
3.76
3.75
3.57
3.56
3.55
1.00
1.00
0.99
1.99
1.00
2.00
1.00
2.00
1.99
3.99
4.00
9.0 8.5 8.0 7.5 7.0 6.5 6.0 5.5 5.0 4.5 4.0 3.5 3.0 2.5 2.0 1.5 1.0 0.5 0.0

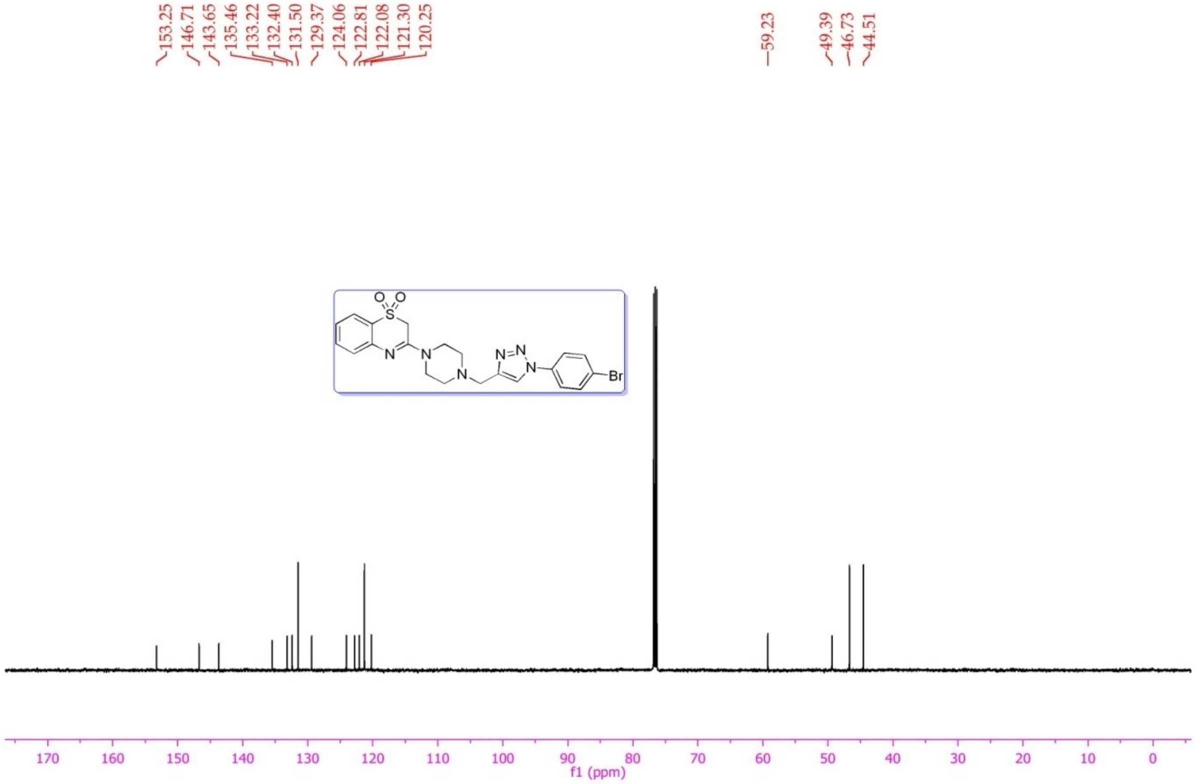
153.25
146.71
143.65
135.46
133.22
132.40
131.50
129.37
124.06
122.81
122.08
121.30
120.25
59.23
49.39
46.73
44.51
170 160 150 140 130 120 110 100 90 80 70 60 50 40 30 20 10 0
f1 (ppm)

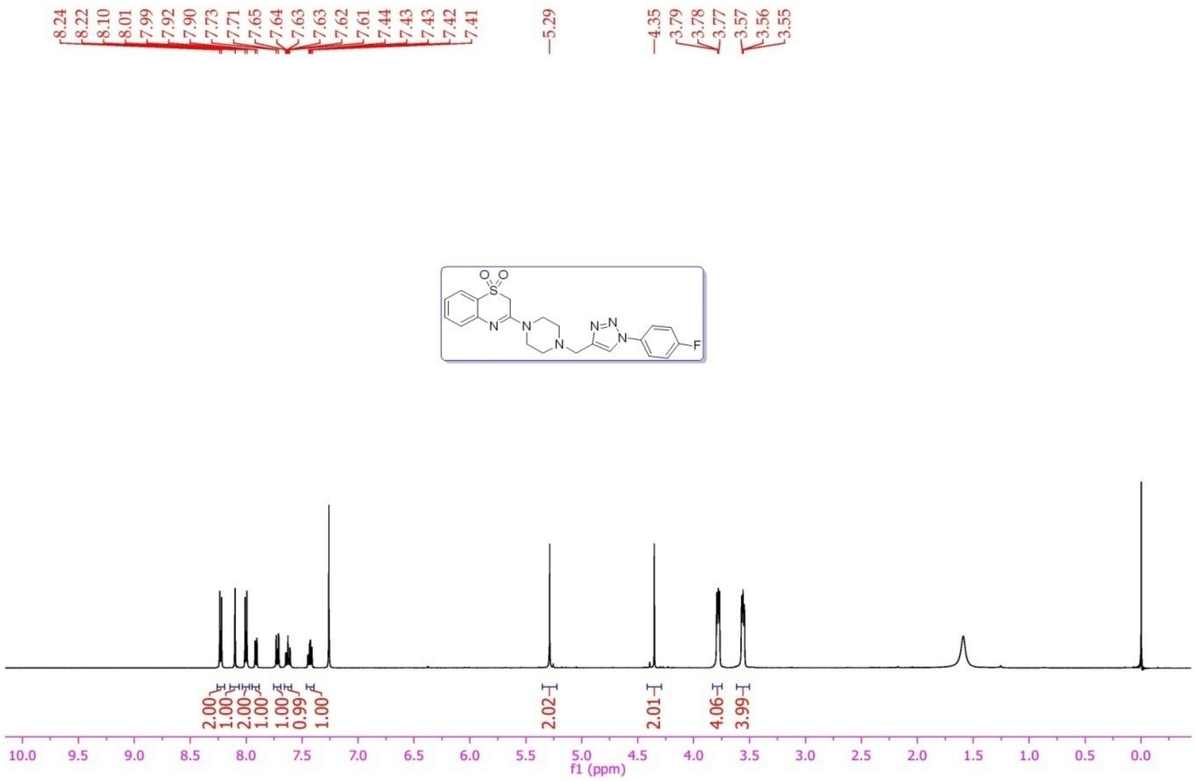
8.24 8.22 8.10 8.01 7.99 7.92 7.90 7.73 7.71 7.65 7.64 7.63 7.63 7.62 7.61 7.44 7.43 7.43 7.42 7.41
5.29
4.35 3.79 3.78 3.77 3.57 3.56 3.55
2.00 1.00 2.00 1.00 1.00 0.99 1.00
2.02
2.01
4.06 3.99
f1 (ppm)

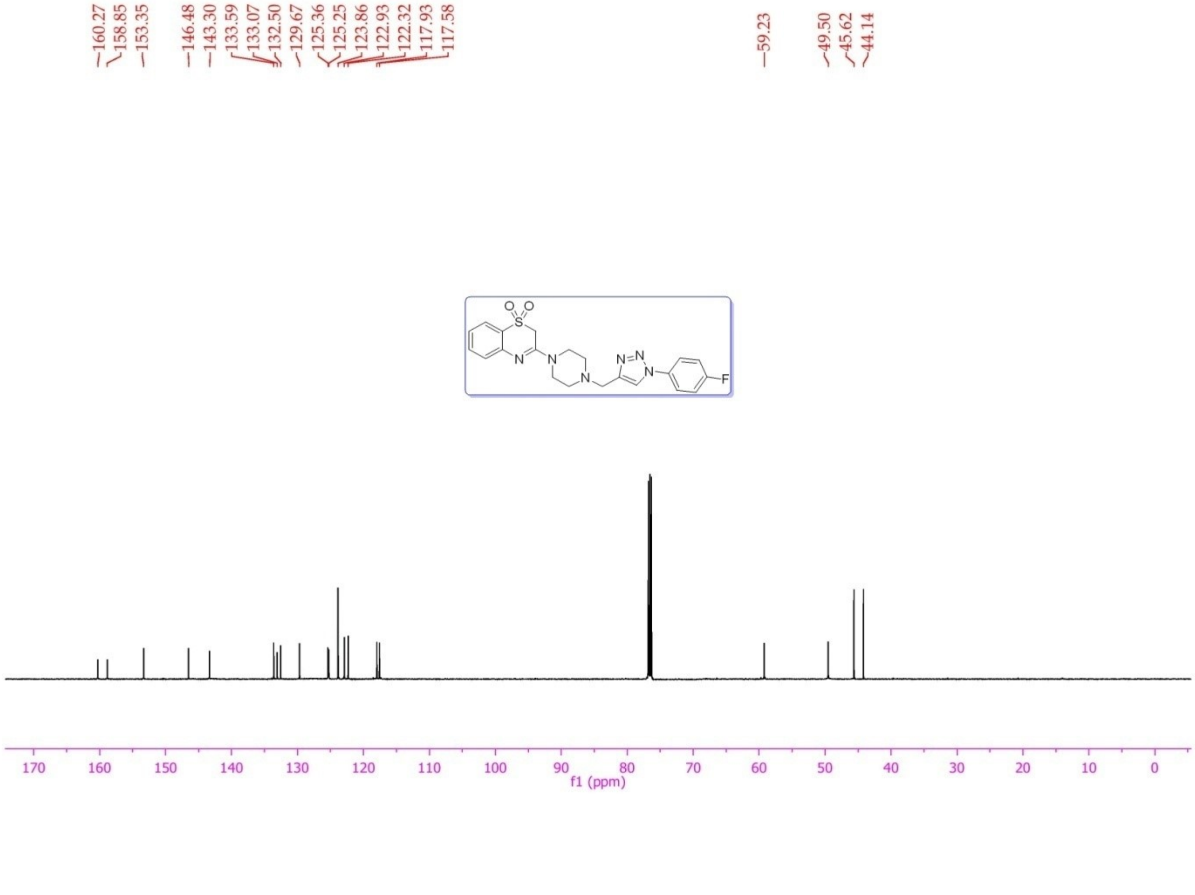
160.27 158.85 153.35 146.48 143.30 133.59 133.07 132.50 129.67 125.36 125.25 123.86 122.93 122.32 117.93 117.58
59.23
49.50 45.62 44.14
f1 (ppm)

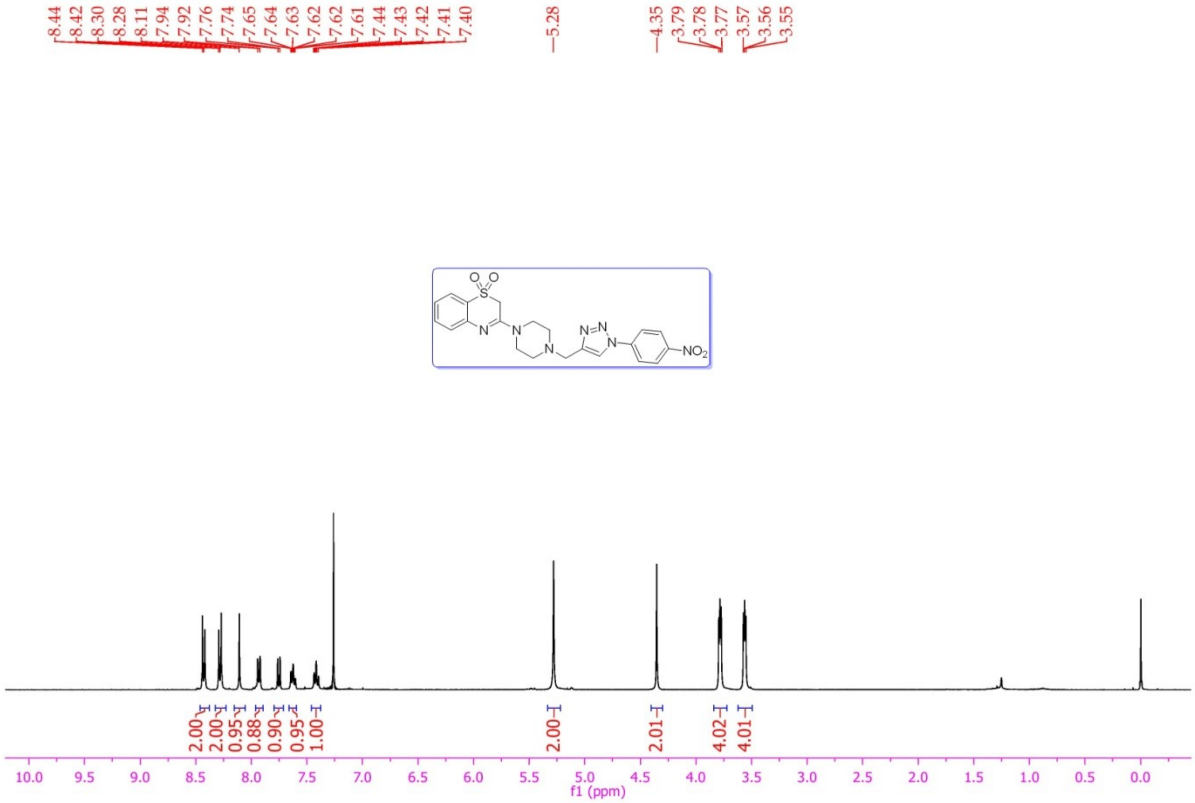
8.44 8.42 8.30 8.28 8.11 7.94 7.92 7.76 7.74 7.65 7.64 7.63 7.62 7.62 7.61 7.44 7.43 7.42 7.41 7.40
5.28
4.35 3.79 3.78 3.77 3.57 3.56 3.55
NO2
2.00 2.00 0.95 0.88 0.90 0.95 1.00
2.00
2.01
4.02 4.01
10.0 9.5 9.0 8.5 8.0 7.5 7.0 6.5 6.0 5.5 5.0 4.5 4.0 3.5 3.0 2.5 2.0 1.5 1.0 0.5 0.0
f1 (ppm)

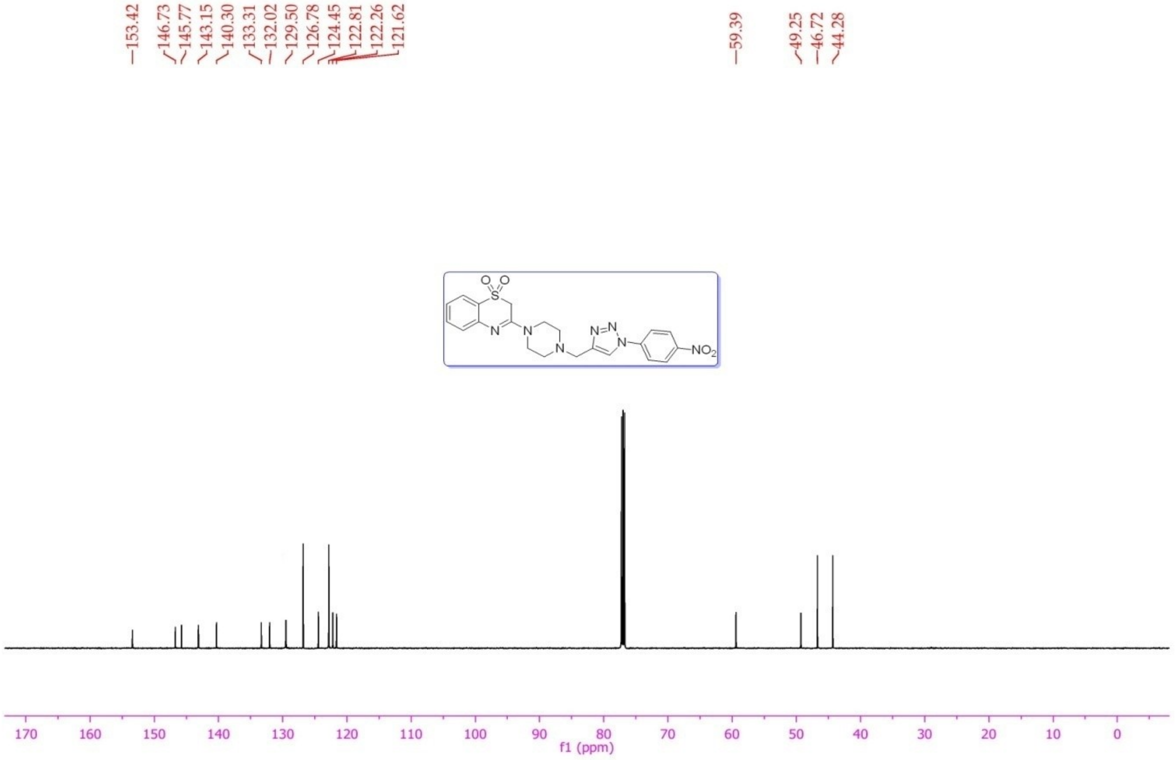
153.42 146.73 145.77 143.15 140.30 133.31 132.02 129.50 126.78 124.45 122.81 122.26 121.62
59.39
49.25 46.72 44.28
NO2
170 160 150 140 130 120 110 100 90 80 70 60 50 40 30 20 10 0
f1 (ppm)

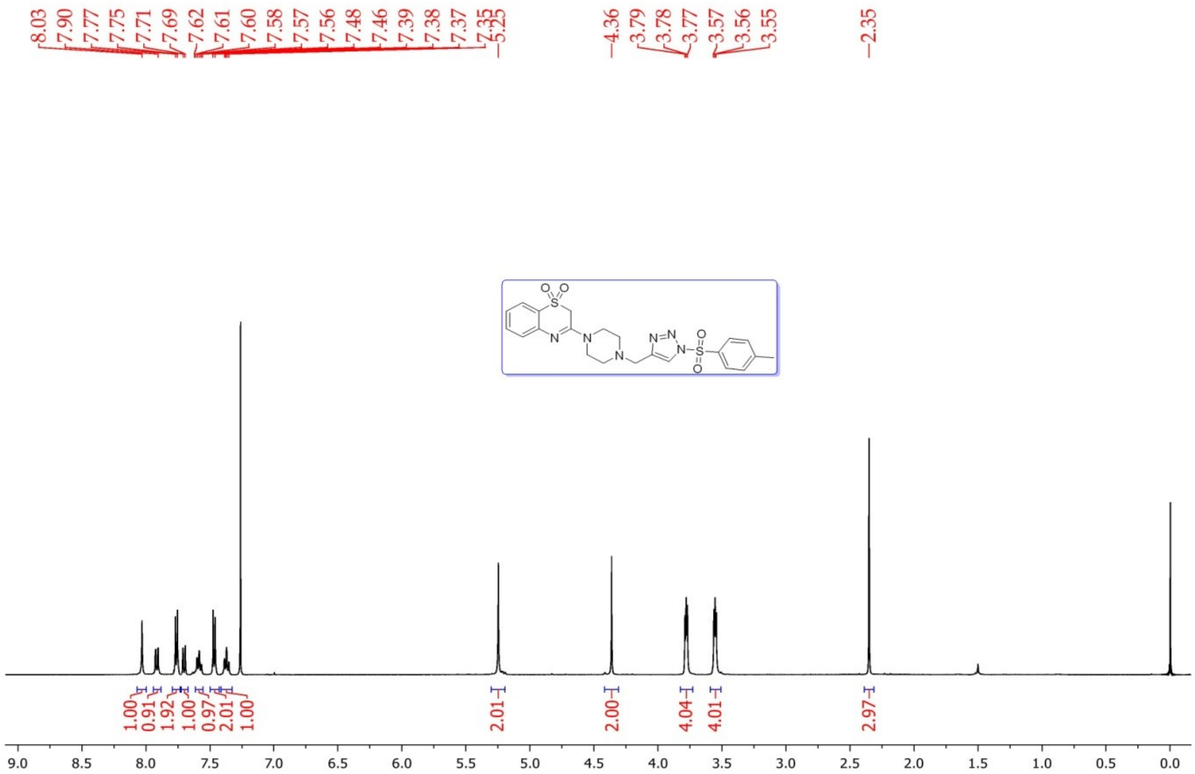
8.03
7.90
7.77
7.75
7.71
7.69
7.62
7.61
7.60
7.58
7.57
7.56
7.48
7.46
7.39
7.38
7.37
7.35
5.25
4.36
3.79
3.78
3.77
3.57
3.56
3.55
2.35
1.00
0.91
1.92
1.00
0.97
2.01
1.00
2.01
2.00
4.04
4.01
2.97
9.0 8.5 8.0 7.5 7.0 6.5 6.0 5.5 5.0 4.5 4.0 3.5 3.0 2.5 2.0 1.5 1.0 0.5 0.0

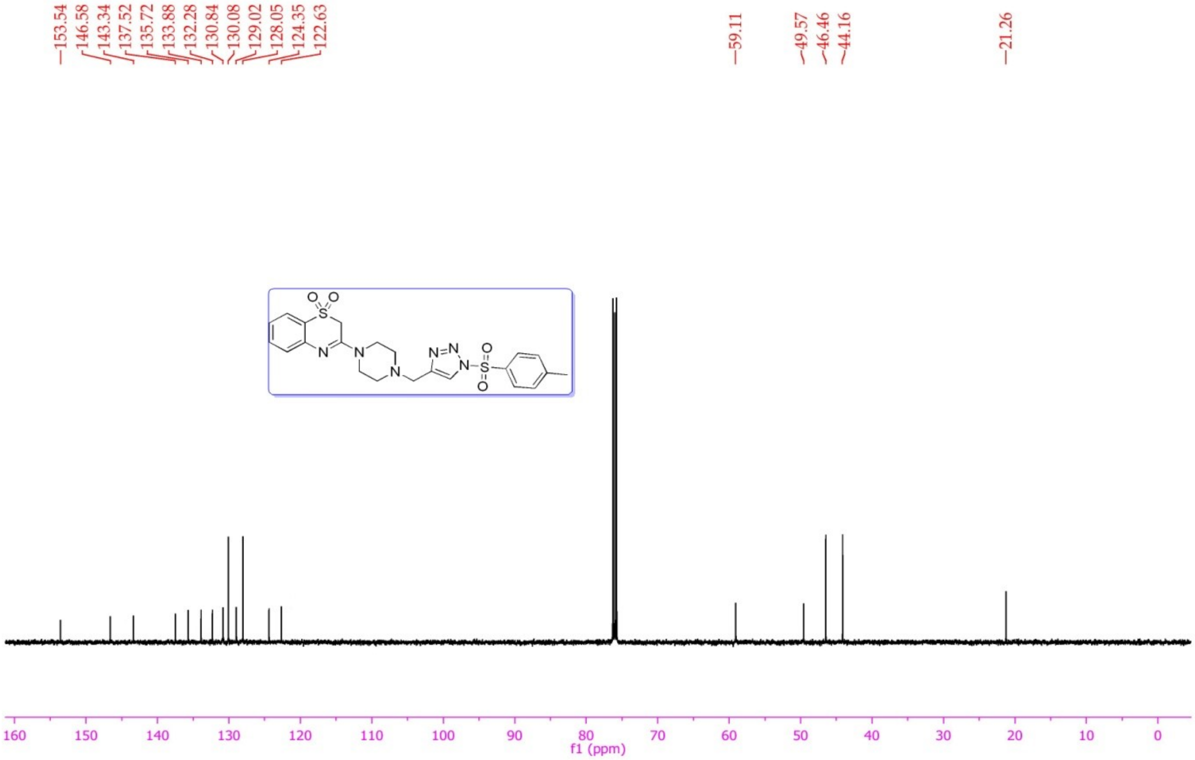
153.54
146.58
143.34
137.52
135.72
133.88
132.28
130.84
130.08
129.02
128.05
124.35
122.63
59.11
49.57
46.46
44.16
21.26
160 150 140 130 120 110 100 90 80 70 60 50 40 30 20 10 0
f1 (ppm)

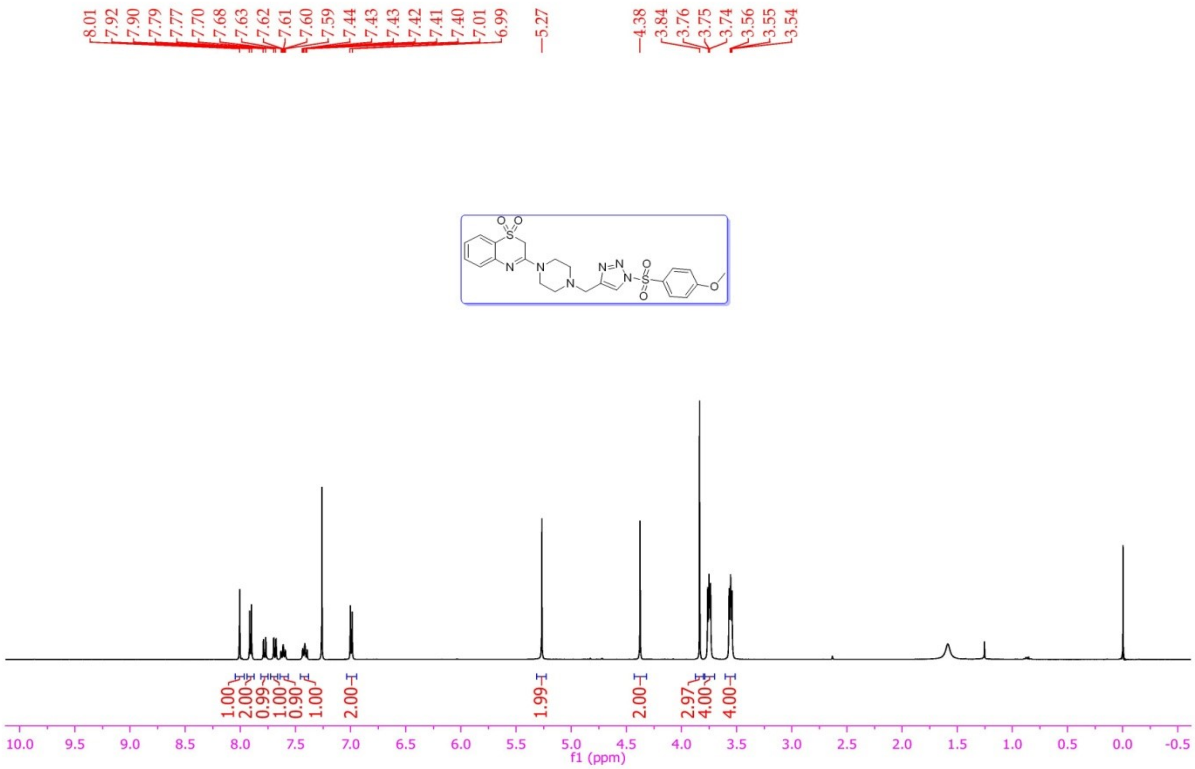
8.01 7.92 7.90 7.79 7.77 7.70 7.68 7.63 7.62 7.61 7.60 7.59 7.44 7.43 7.43 7.42 7.41 7.40 7.01 6.99
5.27
4.38 3.84 3.76 3.75 3.74 3.56 3.55 3.54
1.00 2.00 0.99 1.00 0.90 1.00 2.00 1.99 2.00 2.97 4.00 4.00
10.0 9.5 9.0 8.5 8.0 7.5 7.0 6.5 6.0 5.5 5.0 4.5 4.0 3.5 3.0 2.5 2.0 1.5 1.0 0.5 0.0 -0.5
f1 (ppm)

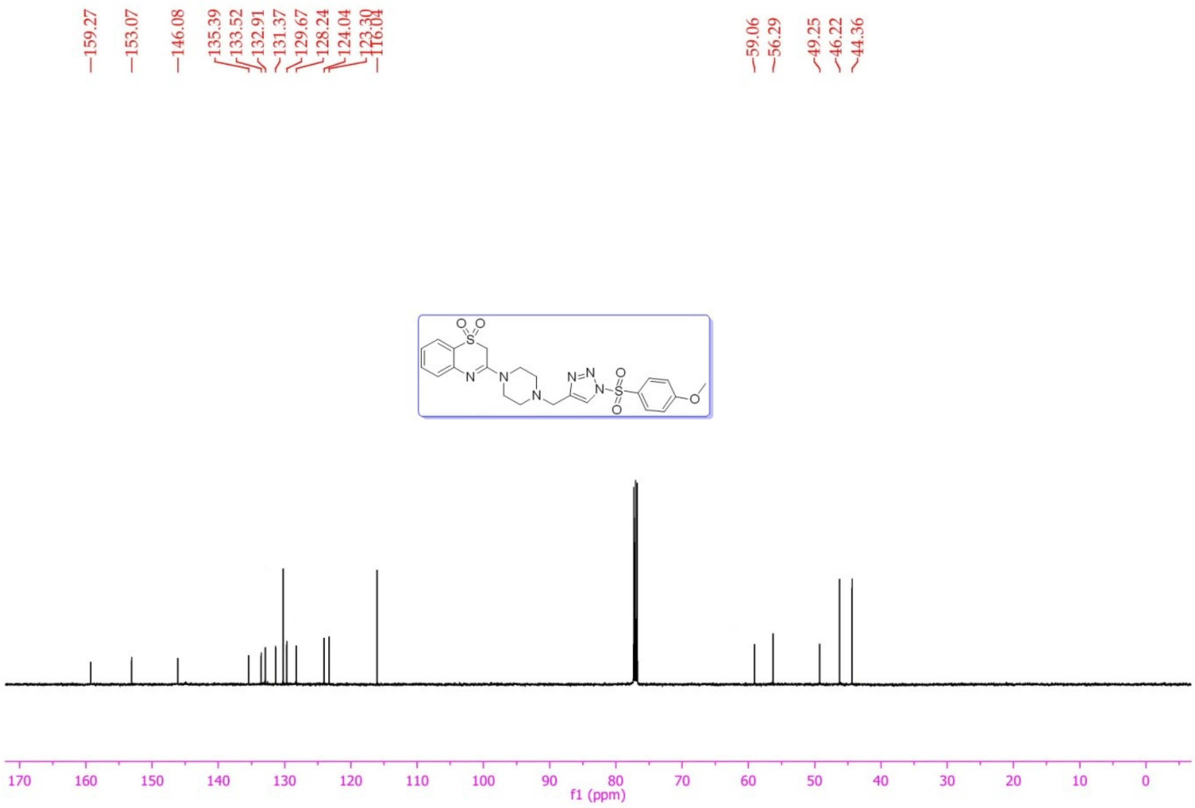
159.27 153.07 146.08 135.39 133.52 132.91 131.37 129.67 128.24 124.04 123.30 116.04
59.06 56.29 49.25 46.22 44.36
170 160 150 140 130 120 110 100 90 80 70 60 50 40 30 20 10 0
f1 (ppm)

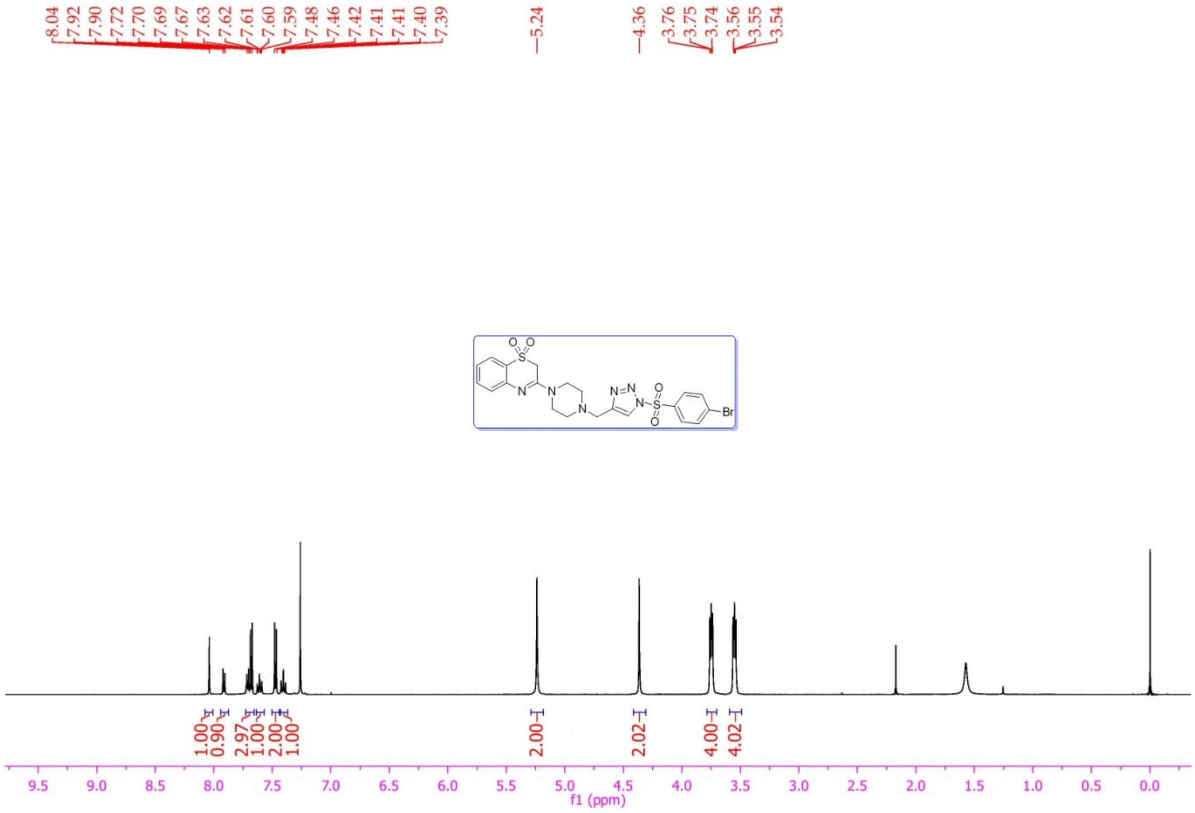
8.04 7.92 7.90 7.72 7.70 7.69 7.67 7.63 7.62 7.61 7.60 7.59 7.48 7.46 7.42 7.41 7.41 7.40 7.39
5.24
4.36
3.76 3.75 3.74 3.56 3.55 3.54
1.00 0.90 2.97 1.00 2.00 1.00
2.00
2.02
4.00
4.02
9.5 9.0 8.5 8.0 7.5 7.0 6.5 6.0 5.5 5.0 4.5 4.0 3.5 3.0 2.5 2.0 1.5 1.0 0.5 0.0
f1 (ppm)

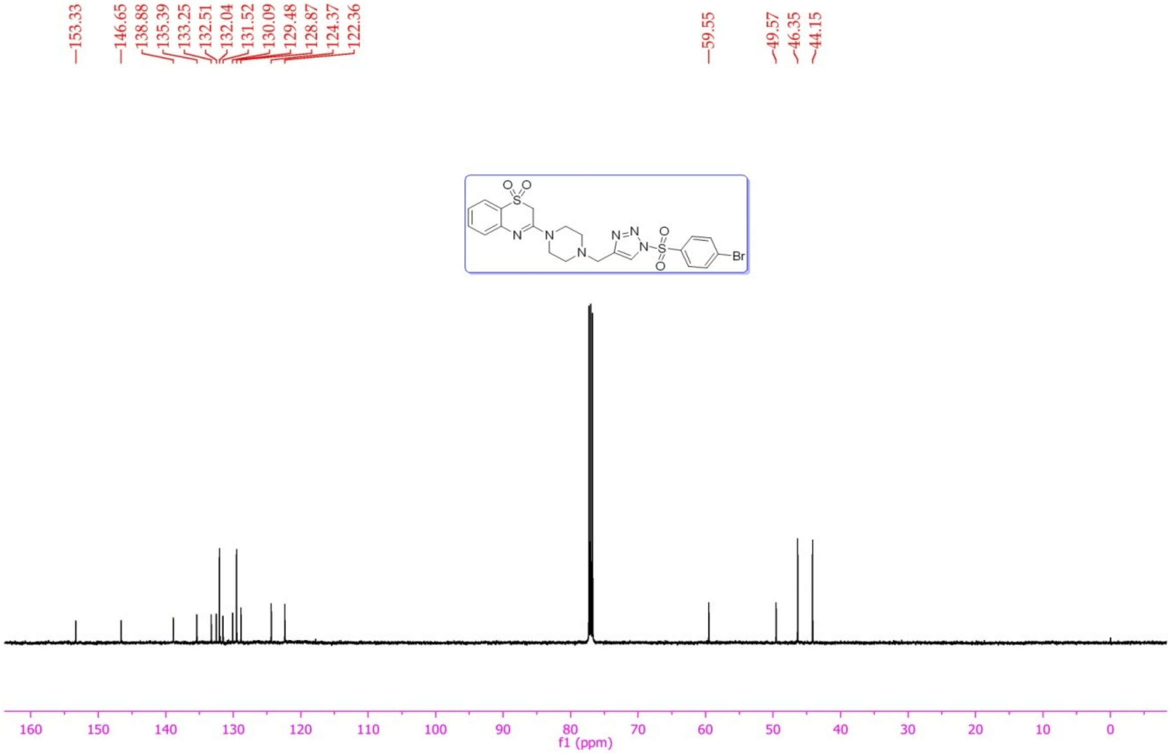
153.33
146.65 138.88 135.39 133.25 132.51 132.04 131.52 130.09 129.48 128.87 124.37 122.36
59.55
49.57 46.35 44.15
160 150 140 130 120 110 100 90 80 70 60 50 40 30 20 10 0
f1 (ppm)

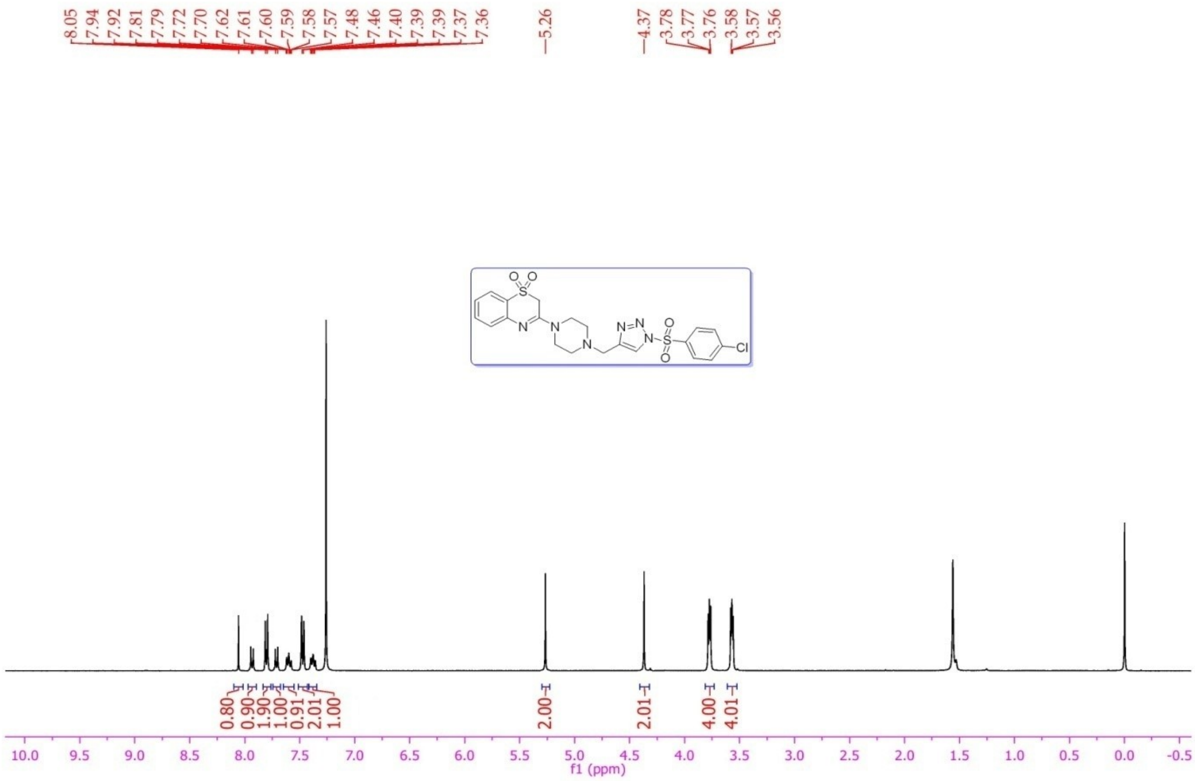
8.05 7.94 7.92 7.81 7.79 7.72 7.70 7.62 7.61 7.60 7.59 7.58 7.57 7.48 7.46 7.40 7.39 7.39 7.37 7.36
5.26
4.37 3.78 3.77 3.76 3.58 3.57 3.56
0.80 0.90 1.90 1.00 0.91 2.01 1.00
2.00
2.01
4.00 4.01
f1 (ppm)

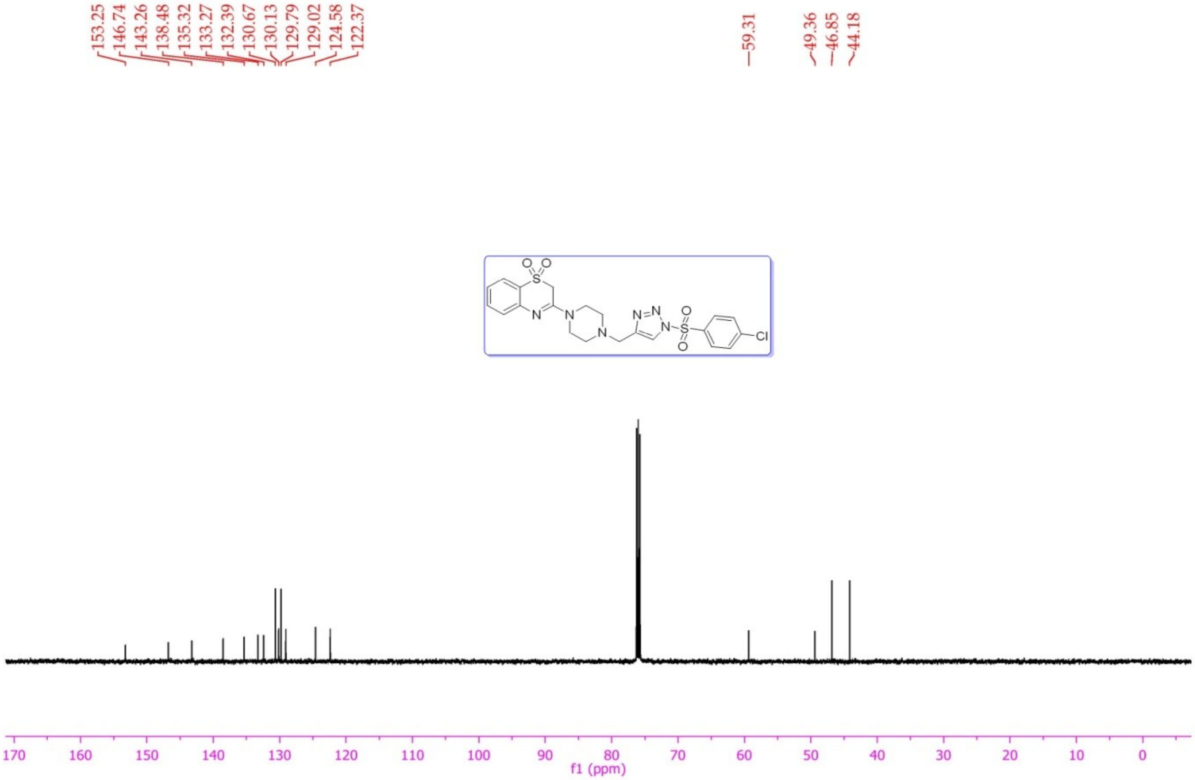
153.25 146.74 143.26 138.48 135.32 133.27 132.39 130.67 130.13 129.79 129.02 124.58 122.37
59.31
49.36 46.85 44.18
f1 (ppm)

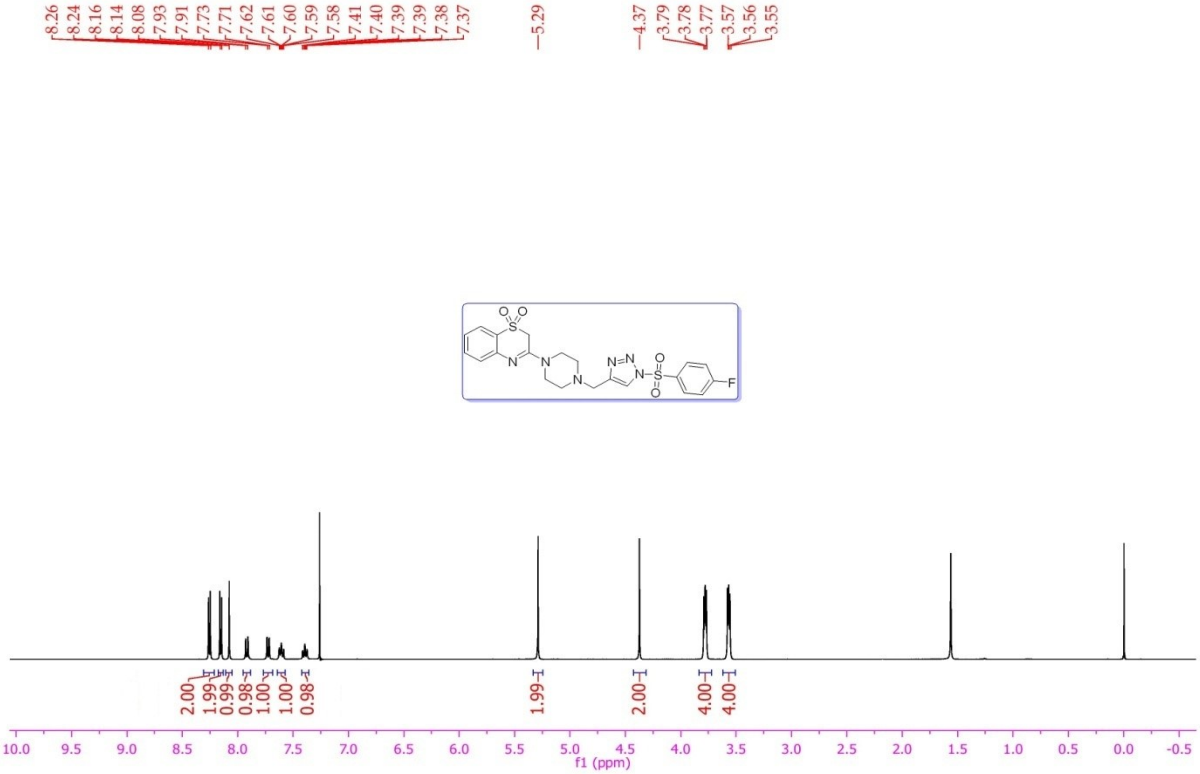

8.26
8.24
8.16
8.14
8.08
7.93
7.91
7.73
7.71
7.62
7.61
7.60
7.59
7.58
7.41
7.40
7.39
7.38
7.37
5.29
4.37
3.79
3.78
3.77
3.57
3.56
3.55
2.00
1.99
0.99
0.98
1.00
1.00
0.98
1.99
2.00
4.00
4.00
10.0 9.5 9.0 8.5 8.0 7.5 7.0 6.5 6.0 5.5 5.0 4.5 4.0 3.5 3.0 2.5 2.0 1.5 1.0 0.5 0.0 -0.5
f1 (ppm)

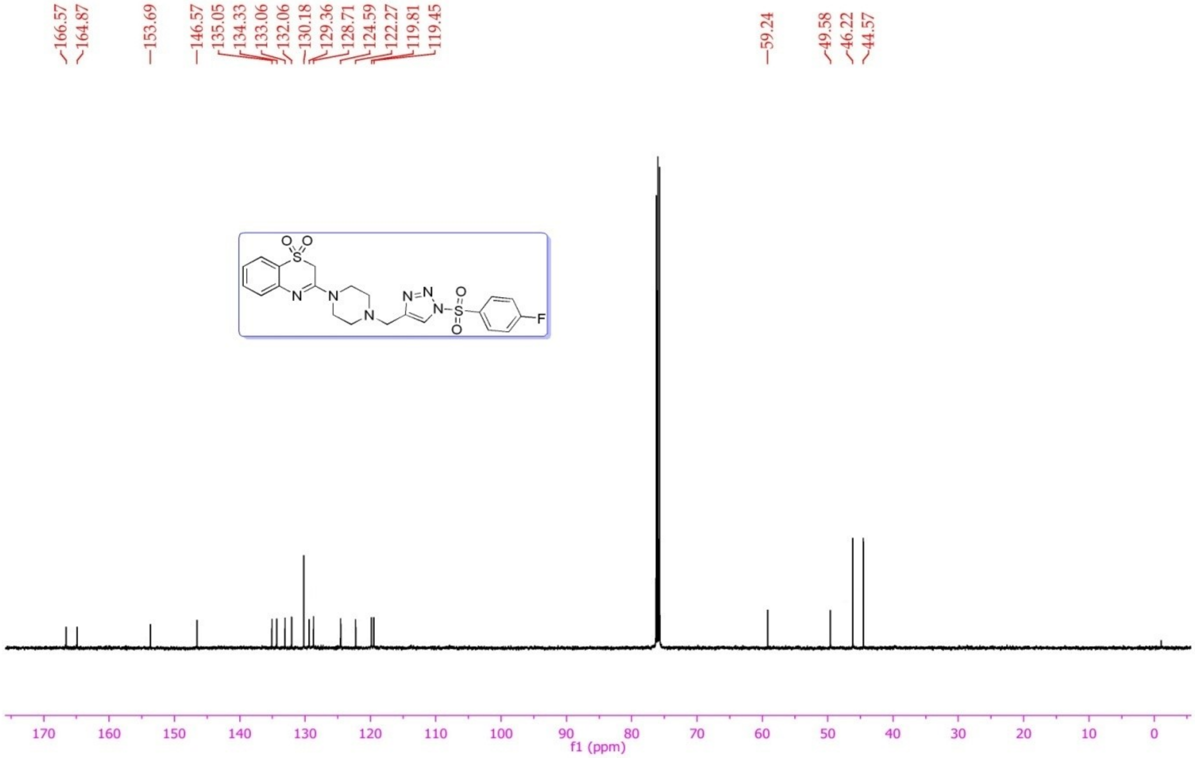

166.57
164.87
153.69
146.57
135.05
134.33
133.06
132.06
130.18
129.36
128.71
124.59
122.27
119.81
119.45
59.24
49.58
46.22
44.57
170 160 150 140 130 120 110 100 90 80 70 60 50 40 30 20 10 0
f1 (ppm)

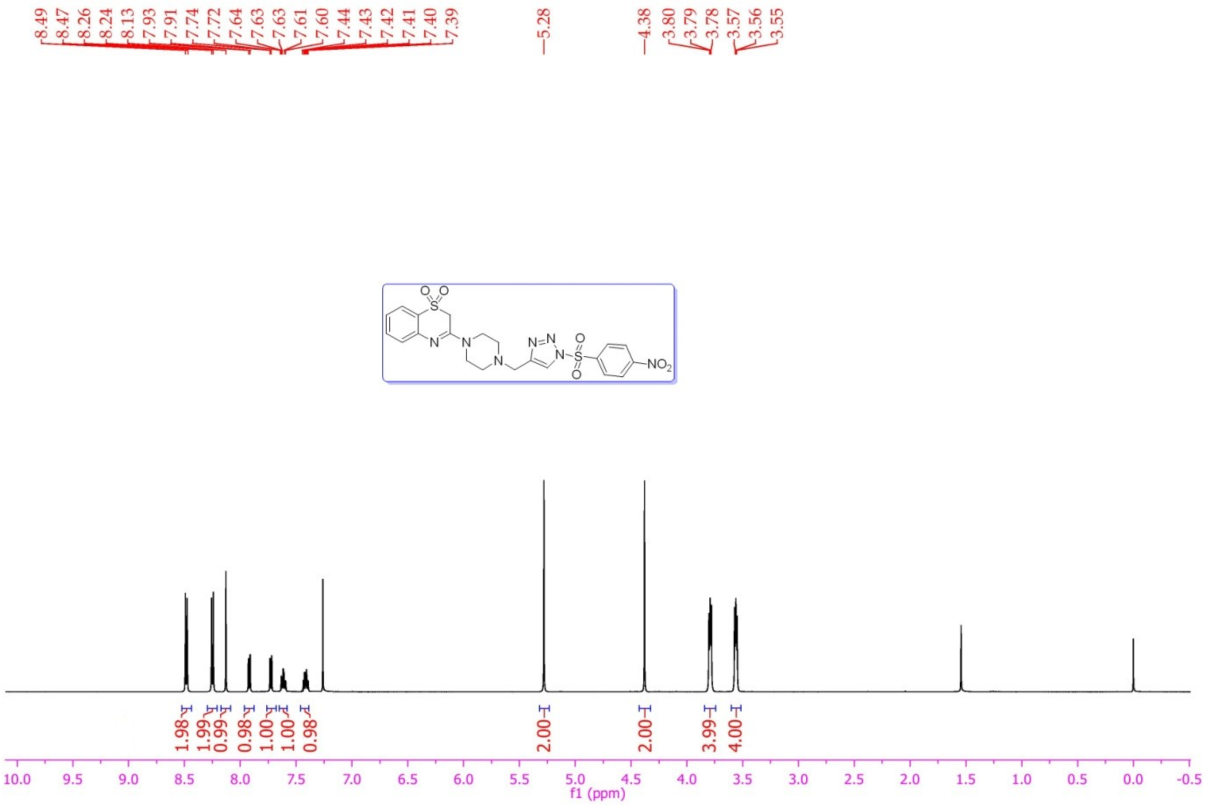
8.49 8.47 8.26 8.24 8.13 7.93 7.91 7.74 7.72 7.64 7.63 7.63 7.61 7.60 7.44 7.43 7.42 7.41 7.40 7.39
5.28
4.38 3.80 3.79 3.78 3.57 3.56 3.55
NO2
1.98 1.99 0.99 0.98 1.00 1.00 0.98
2.00
2.00
3.99 4.00
10.0 9.5 9.0 8.5 8.0 7.5 7.0 6.5 6.0 5.5 5.0 4.5 4.0 3.5 3.0 2.5 2.0 1.5 1.0 0.5 0.0 -0.5
f1 (ppm)

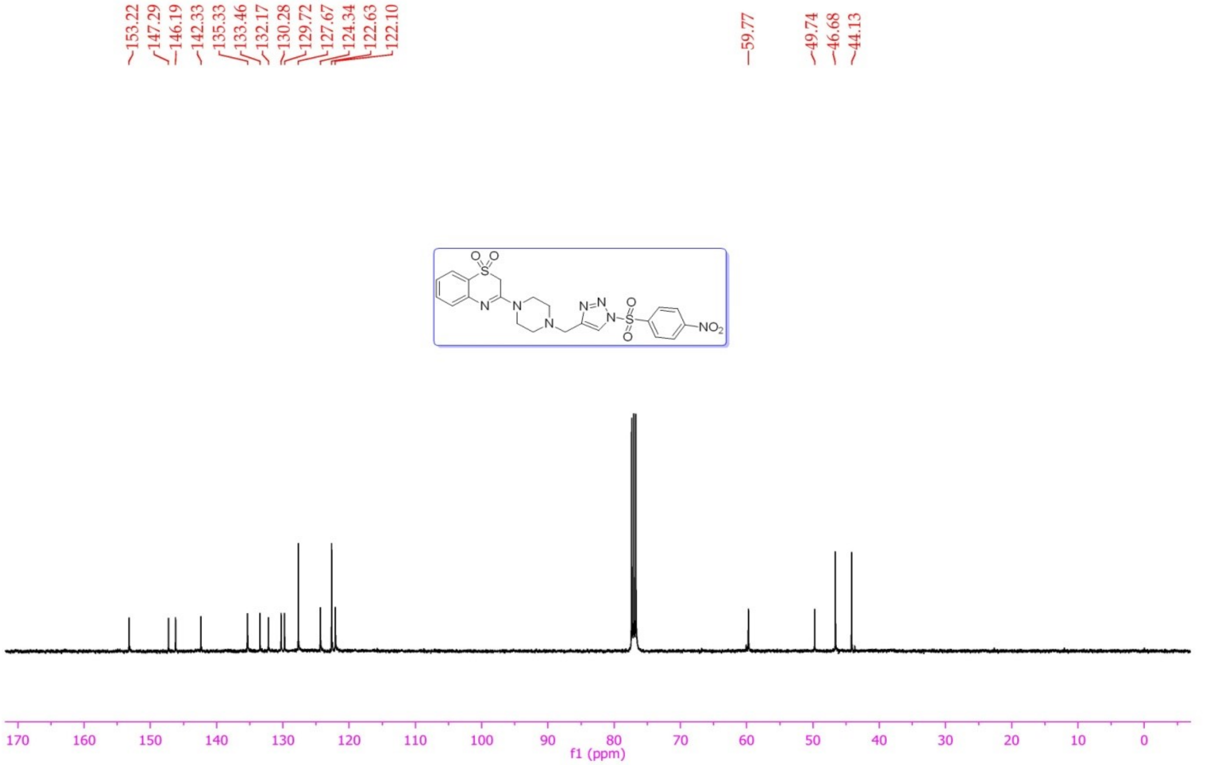
153.22 147.29 146.19 142.33 135.33 133.46 132.17 130.28 129.72 127.67 124.34 122.63 122.10
59.77
49.74 46.68 44.13
NO2
170 160 150 140 130 120 110 100 90 80 70 60 50 40 30 20 10 0
f1 (ppm)

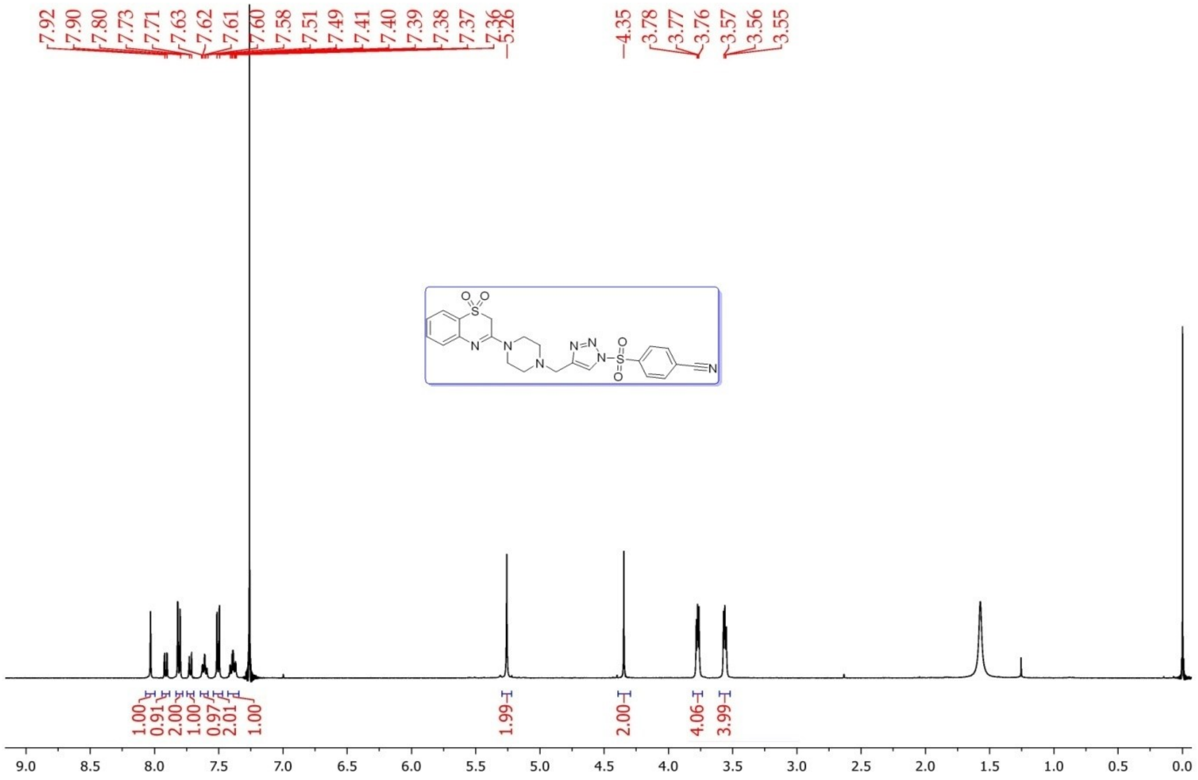

7.92
7.90
7.80
7.73
7.71
7.63
7.62
7.61
7.60
7.58
7.51
7.49
7.41
7.40
7.39
7.38
7.37
7.36
5.26
4.35
3.78
3.77
3.76
3.57
3.56
3.55
1.00
0.91
2.00
1.00
0.97
2.01
1.00
1.99
2.00
4.06
3.99
9.0 8.5 8.0 7.5 7.0 6.5 6.0 5.5 5.0 4.5 4.0 3.5 3.0 2.5 2.0 1.5 1.0 0.5 0.0

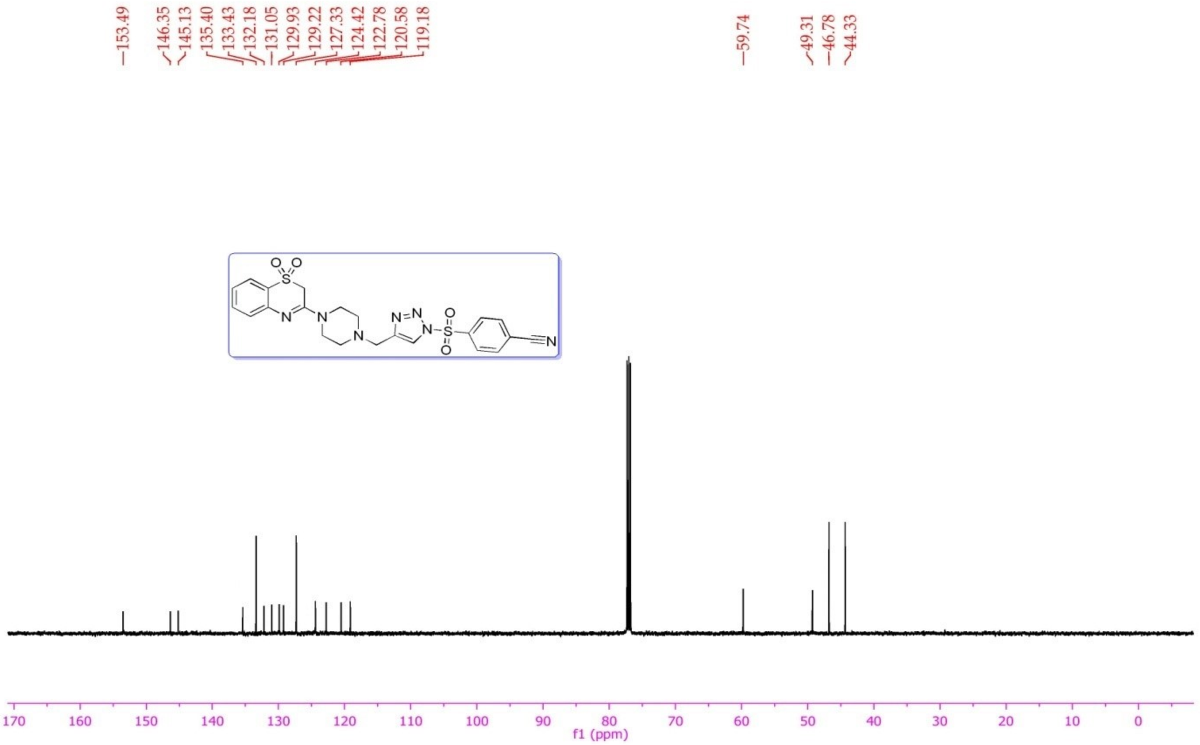

153.49
146.35
145.13
135.40
133.43
132.18
131.05
129.93
129.22
127.33
124.42
122.78
120.58
119.18
59.74
49.31
46.78
44.33
170 160 150 140 130 120 110 100 90 80 70 60 50 40 30 20 10 0
f1 (ppm)

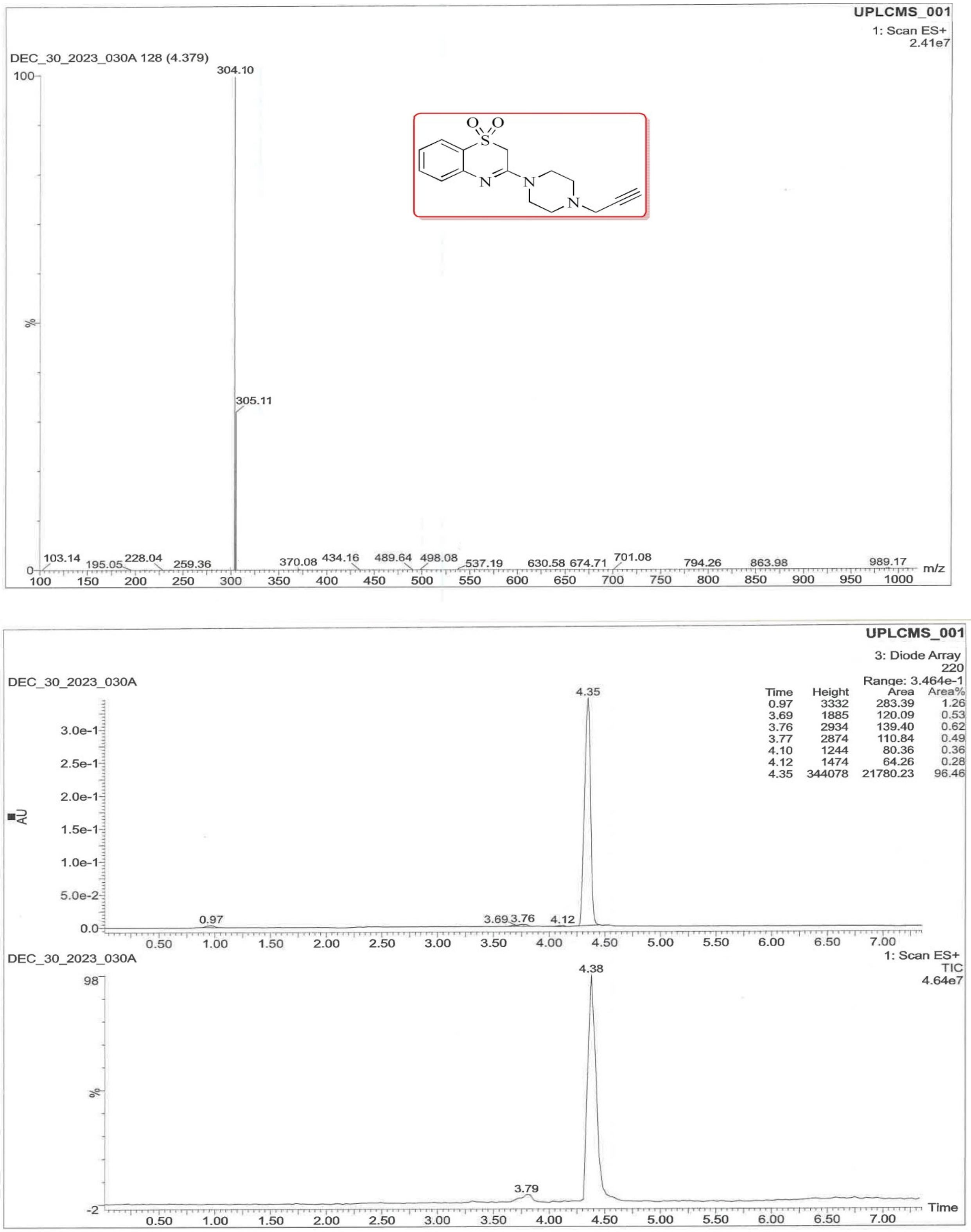

LCMS of compound 4

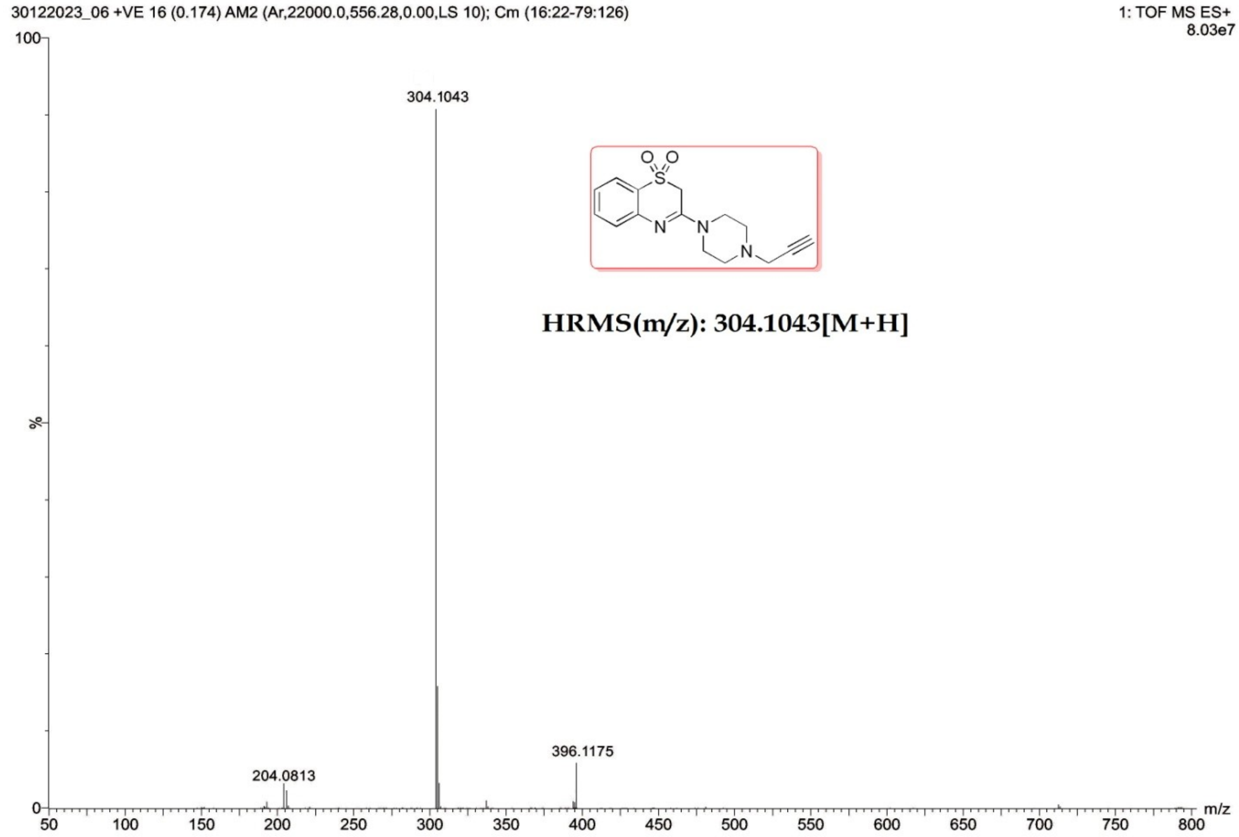
30122023_06 +VE 16 (0.174) AM2 (Ar,22000.0,556.28,0.00,LS 10); Cm (16:22-79:126)
1: TOF MS ES+
8.03e7
304.1043
396.1175
204.0813
%
m/z
HRMS(m/z): 304.1043[M+H]

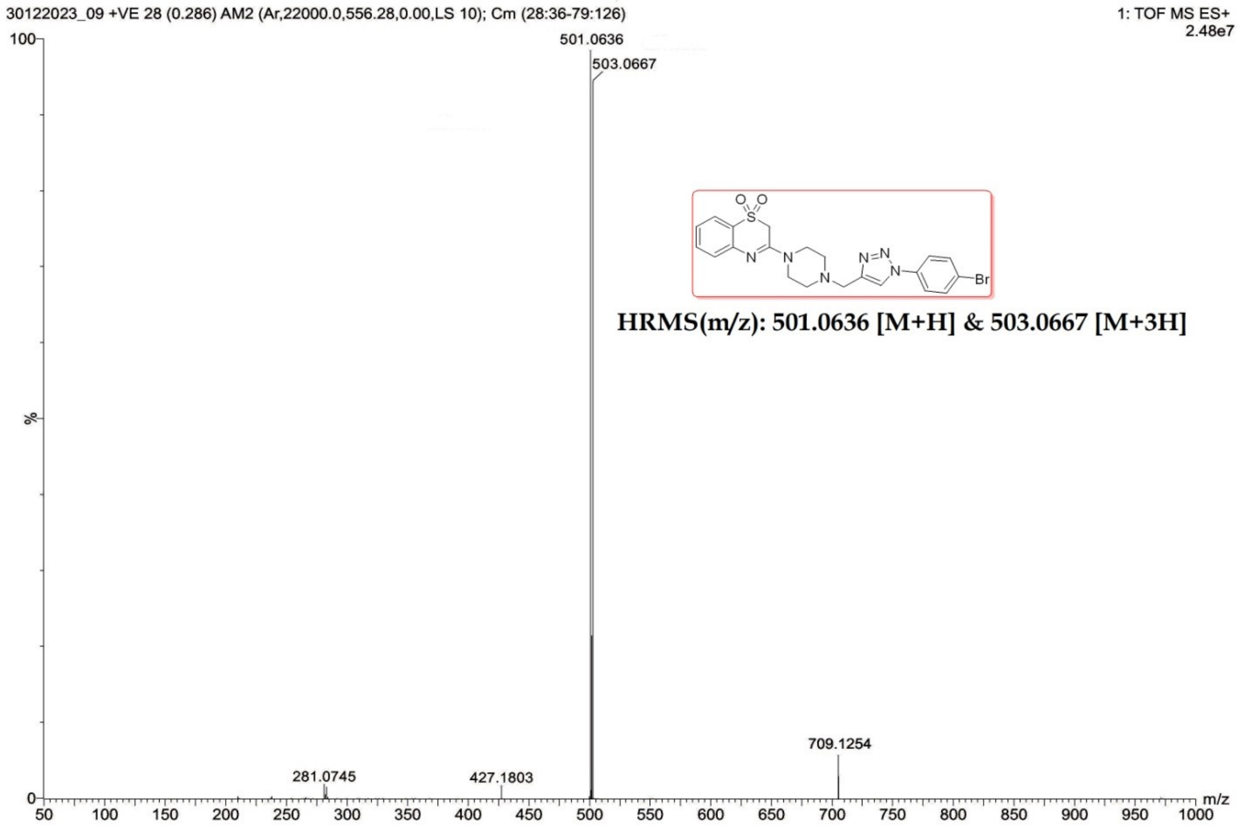
30122023_09 +VE 28 (0.286) AM2 (Ar,22000.0,556.28,0.00,LS 10); Cm (28:36-79:126)
1: TOF MS ES+
2.48e7
501.0636
503.0667
709.1254
281.0745
427.1803
%
m/z
HRMS(m/z): 501.0636 [M+H] & 503.0667 [M+3H]

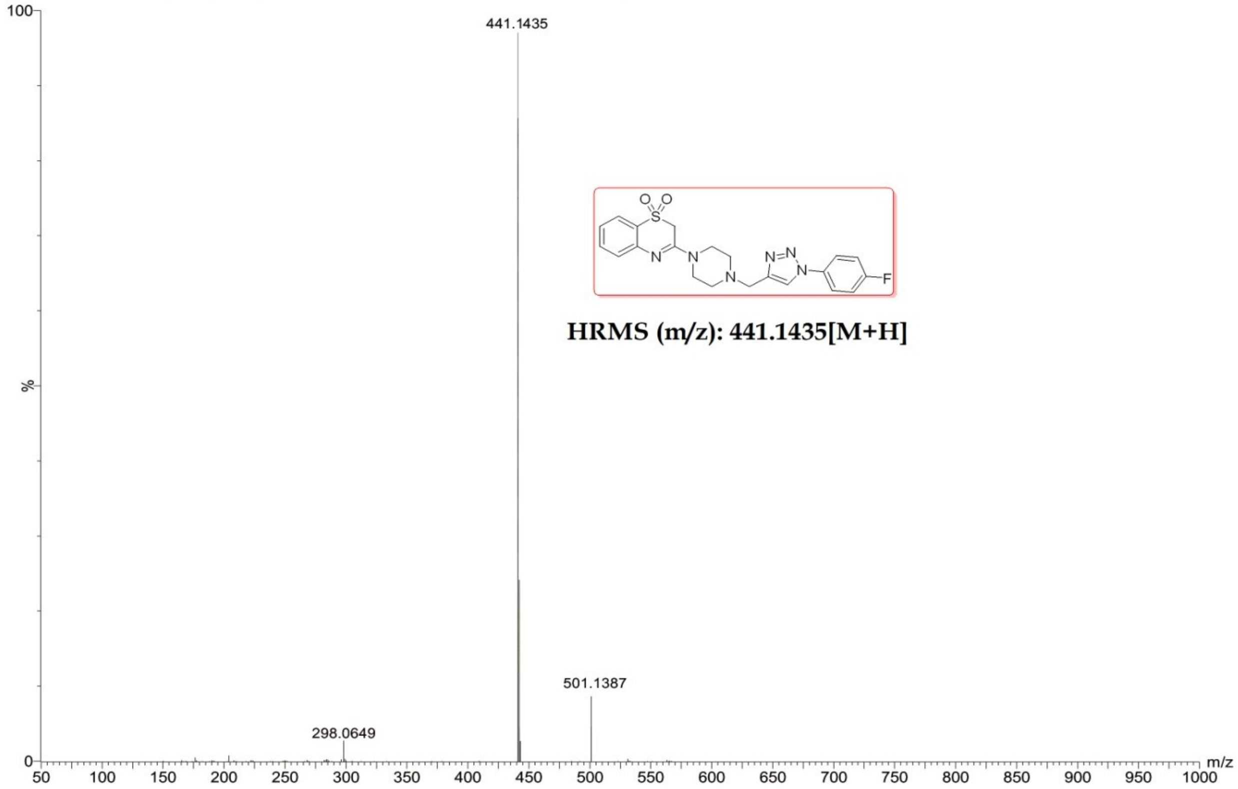
441.1435
501.1387
298.0649
HRMS (m/z): 441.1435[M+H]
100
%
0
m/z
50
100
150
200
250
300
350
400
450
500
550
600
650
700
750
800
850
900
950
1000

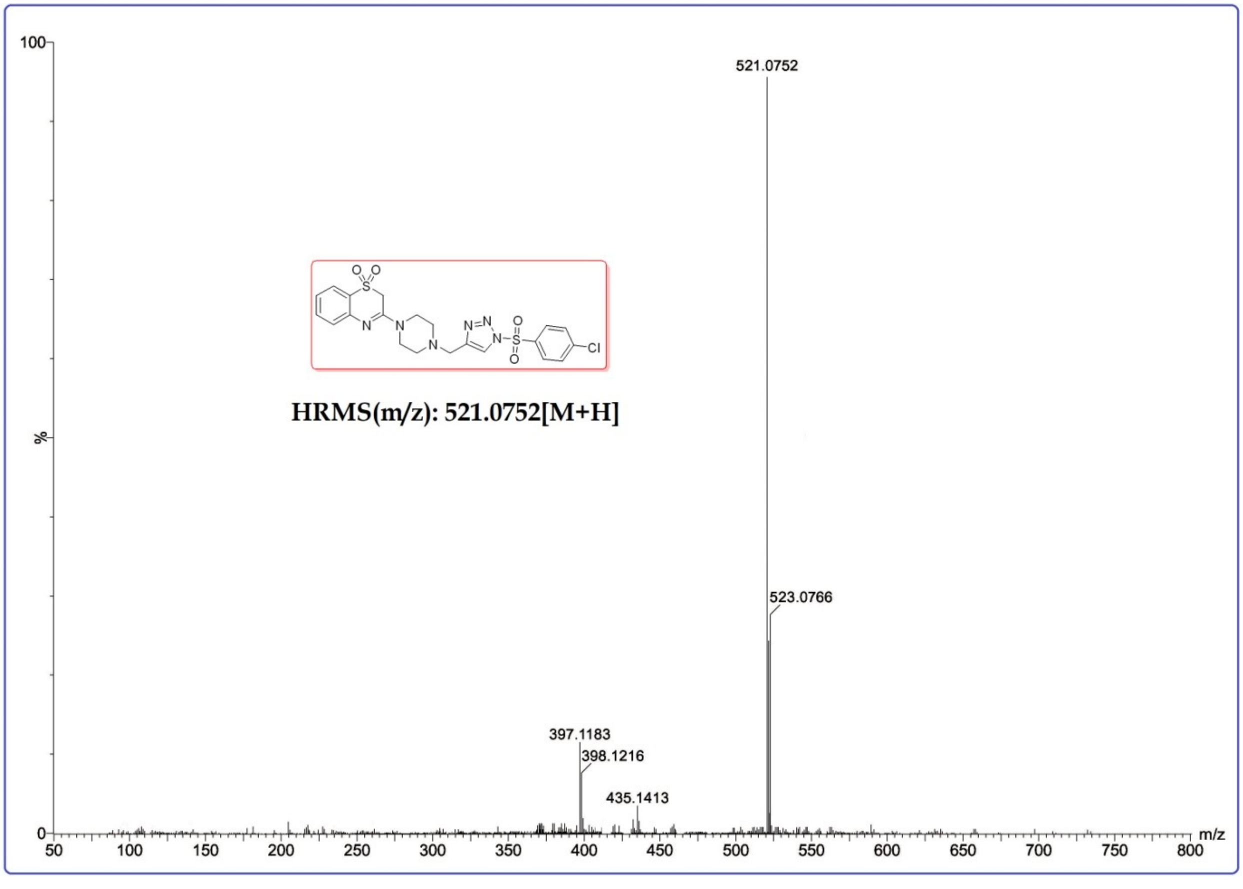
521.0752
523.0766
397.1183
398.1216
435.1413
HRMS(m/z): 521.0752[M+H]
100
%
0
m/z
50
100
150
200
250
300
350
400
450
500
550
600
650
700
750
800

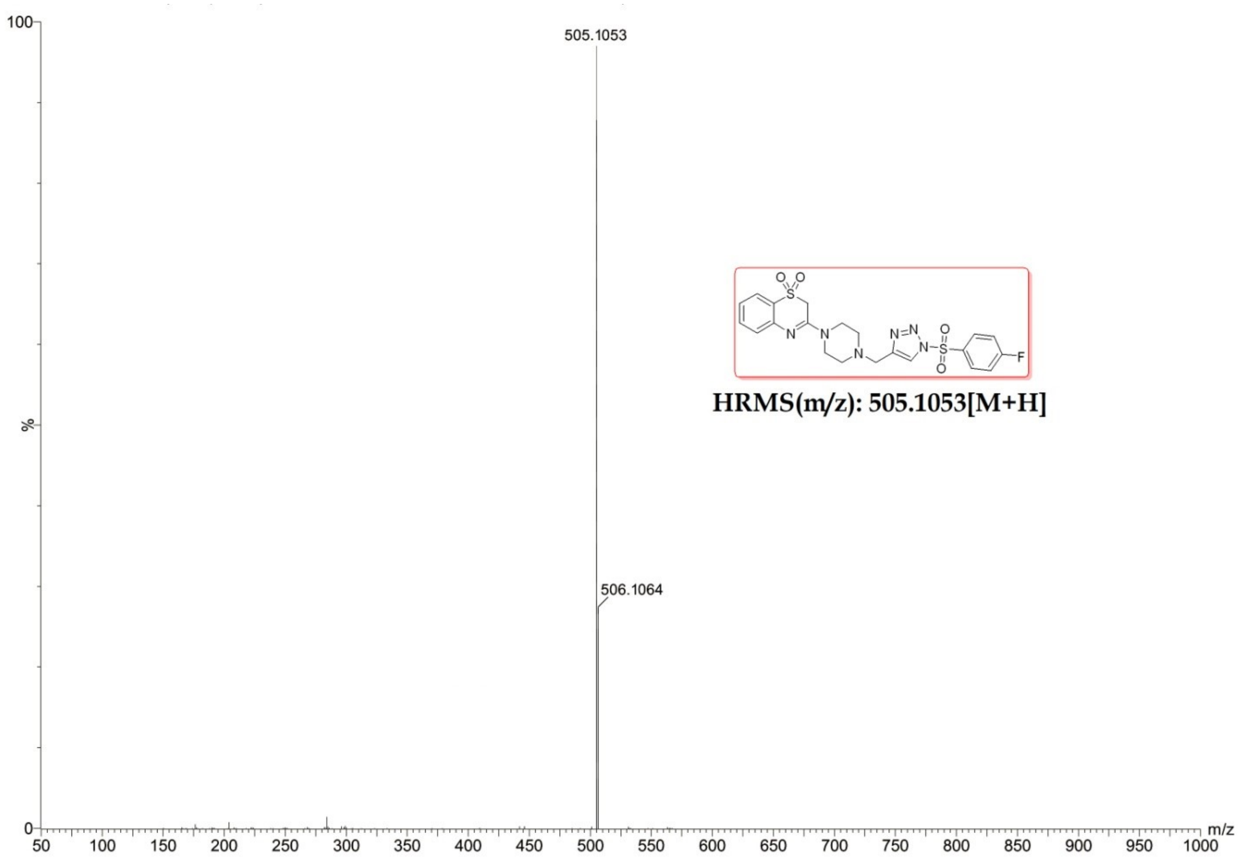
100
505.1053
%
506.1064
0
m/z
50
100
150
200
250
300
350
400
450
500
550
600
650
700
750
800
850
900
950
1000
HRMS(m/z): 505.1053[M+H]
